# ﻿Revision of the Oriental and Australasian diving beetle genus *Sandracottus* Sharp, 1882 (Coleoptera, Dytiscidae, Dytiscinae)

**DOI:** 10.3897/zookeys.1223.138220

**Published:** 2025-01-06

**Authors:** Lars Hendrich, Michel Brancucci

**Affiliations:** 1 SNSB - Zoologische Staatssammlung München, Münchhausenstraße 21, D – 81247 München, Germany; 2 Naturhistorisches Museum Basel, Basel, Switzerland; † Deceased

**Keywords:** Australasia, conservation, Indomalayan region, lectotype designation, neotype designation, new status, new synonymies, systematics, zoogeography

## Abstract

A comprehensive revision is presented of the Oriental and Australasian diving beetle genus *Sandracottus* Sharp, 1882 (Coleoptera: Dytiscidae: Dytiscinae: Aciliini) and seven junior subjective synonyms are proposed. *Sandracottusguerini* Balfour-Browne, 1939, **syn. nov.** is a junior subjective synonym of *S.femoralis* Heller, 1934; *S.manipurensis* Vazirani, 1969, **syn. nov.** of *S.hunteri* (Crotch, 1872); *S.mixtus* Blanchard, 1843, **syn. nov.** of *S.chevrolati* (Aubé, 1838); and *S.angulifer* Heller, 1934, **syn. nov.**, *S.nauticus* Sharp, 1882, **syn. nov.**, and *S.palawanensis* Satô, 1978, **syn. nov.** of *S.maculatus* (Wehncke, 1876). Finally, *S.vijayakumari* Anand et al., 2021, **syn. nov.** is a new synonym of *S.dejeanii* (Aubé, 1838). New status is assigned to *S.bakewelliiguttatus* (Sharp, 1882), **stat. nov.** as well as *S.hunteri* (Crotch, 1872), **stat. rev.** Lectotypes are designated for the following taxa: *Dytiscusflavocinctus* Guérin-Méneville, 1830, *Hydaticuschevrolati* Aubé, 1838, *Hydaticusinsignis* Wehncke, 1876, *Sandracottusbaeri* Régimbart, 1899, *Sandracottusbizonatus* Régimbart, 1899, and *Sandracottusornatus* Sharp, 1882. A neotype is designated for *Hydaticusmaculatus* Wehncke, 1876. In total, three Oriental species, two of which also occur in the East Palaearctic, six Southeast Asian species, one species from New Guinea and the Moluccas, and one from Australia with an endemic subspecies in Central Australia (*S.bakewelliiguttatus*) are recognised. Each taxon is presented with a diagnosis, habitat preferences, conservation assessments, distribution data, and a comprehensive bibliography. Important characters (habitus, dorsal colouration, median lobes and parameres) are illustrated. All currently valid taxa are redescribed. *Sandracottusjaechi* Wewalka & Vazirani, 1975 from Sri Lanka, *S.bizonatus* from Borneo, *S.insignis* from the Philippines and *S.rotundus* Sharp, 1882 from Sulawesi are recommended to be listed in the next IUCN red data book. A key to all species is provided.

## ﻿Introduction

The genus *Sandracottus* Sharp, 1882 belonging to the subfamily Dytiscinae and tribe Aciliini is revised. Most of the species occur in the Indian and Indomalayan regions where the genus represents, together with some members of the genus *Hydaticus*, the most colourful larger predaceous water beetles of the local fauna. To date, 17 species and one subspecies have been recognised from Asia and Australia (see Anand et al. 2021; Nilsson and Hájek 2024).

*Sandracottus* has not been subjected previously to a comprehensive revision. In this paper each species is presented with a diagnosis and redescription, habitat preferences, conservation assessment, distribution data and a comprehensive bibliography. Important species characters [habitus, dorsal colouration, median lobes and parameres] are illustrated, and finally a key to all species is provided.

The material dealt with in this paper was mainly collected during several field trips between the years 1990 and 2020 by Lars Hendrich and Michael Balke (Munich, Germany), Michel Brancucci † (Basel, Switzerland), Jan Haft (Dorfen, Germany), Andre Skale (Hof/Saale, Germany), Manfred Jäch, Günther Wewalka, Helena Shaverdo, Harald Schillhammer (Vienna, Austria), Wisrutta Atthakor (Bangkok, Thailand), Hendrik Freitag (Manila, Philippines), the staff of the Zoological Reference Collection in Singapore (e.g., Yang Chan Man), Paolo Mazzoldi (Brescia, Italy), and Jaroslav Šťastný and Jiří Hájek (Czech Republic) to Iran, India, China, Southeast Asia, New Guinea, and Australia. Furthermore, all historical specimens of *Sandracottus* deposited in the relevant museum and private collections of the world, including most of the type material, were studied for this revision.

The species of *Sandracottus* are lentic and inhabit temporary or semipermanent pools and ponds, often rich in decaying leaves and twigs. Particularly the Indomalayan and New Guinean species are restricted to shaded forest pools or shallow rest pools of intermittent forest streams and springs (e.g., Hendrich 1995; Hendrich and Balke 1995; Balke et al. 2004; Suprayitno et al. 2017; Atthakor et al. 2018; Alarie et al. 2023).

In freshwater management world-wide, the family Dytiscidae proved to be an important biomonitoring group. Many *Sandracottus* species, especially in Southeast Asia, are restricted to lentic habitats in primary or at least old second growth forests. Due to their habitat requirements and limited number of species they represent an ideal target group for environmental impact assessments (EIAs), conservation assessments and biodiversity studies in a wider sense, especially of freshwater habitats at rainforest sites.

## ﻿Materials and methods

### ﻿Material

This study is based on the examination on the adult stage of 1649 specimens, deposited in institutional and private collections listed below. Type specimens were re-examined for most species. New synonyms were based on comparisons of types.

### ﻿Descriptions

Beetles were studied with a Leica MZ 12.5 dissecting microscope at 10–100×. Male genitalia were studied and figured in dry condition. The terminology to denote the orientation of the genitalia follows Miller and Nilsson (2003). Abbreviations used in the text are **TL** = total length, measurements of length from head to apex of elytra; **TL-h** = total length minus head length, measurement of length from anterior margin of pronotum to apex of elytra; and **TW** = maximum width of body measured at right angle to TL. Label data of type material are cited between quotation marks.

### ﻿Photographs and illustrations

Images were taken with a Canon EOS 550D camera fitted with either a 65 mm or MPE65 macro lens, attached to a Stackmaster macrorail (Stonemaster: www.stonemaster-onlineshop.de). Illumination was with two Canon Speedlite 430EX III-RT flashlights and translucent paper diffusors or 3 Stonemaster LED-Segments SN-1. Image stacks were assembled using Helicon Focus software (method A) and cleaned using Adobe Photoshop CS6 software.

Coordinates are given in decimal notation unless cited verbatim from labels. Besides various Australian road maps, we also used Google Earth (http://earth.google.com) to locate several localities; our maps are based on “MICROSOFT ENCARTA World-Atlas 2000”.

#### ﻿Codens

The material used for this study is deposited in the following 34 institutional and private collections:

**AM**Australian Museum, Sydney, New South Wales, Australia (C. Reid)

**ANIC**Australian National Insect Collection, Canberra, Australia (T. Weir)

**NHMUK**Natural History Museum, London, England (M. Barclay)

**CAS** Collection André Skale, Gera, Germany

**CGW** Collection Günther Wewalka, Vienna, Austria

**CHH** Collection Hans Hebauer, Rain/Ndb., Germany

**CHF** Collection Hans Fery, Berlin, Germany (property of NMW)

**CJS** Collection Jaroslav Šťastný, Liberec, Czech Republic

**CLH** Collection Lars Hendrich, Berlin, Germany (property of the NMW)

**CLJW** Collection Liang-Jong Wang Taipei, Taiwan

**CLW** Collection Leopold Wendlandt, Greifswald, Germany

**CPM** Collection Paolo Mazzoldi, Brescia, Italy

**CSUT** Collection Srinakharinwirot University, Thailand (W. Atthakor)

**DEI**Deutsches Entomologisches Institut, Müncheberg, Germany (V. Ferreira)

**HMUG**Hunterian Museum, University of Glasgow, Scotland (G. Hancock)

**NMPC**Národní muzeum, Praha, Czech Republic (J. Hájek)

**MNHN**Muséum national d´Histoire naturelle, Paris, France (H. Perrin)

**MZB**Museum Zoologicum Bogoriense, Cibinong, Indonesia

**NTM**Northern Territory Museum, Darwin, Northern Territories, Australia (G. Dally)

**NMB**Naturhistorisches Museum Basel, Schweiz (M. Borer)

**NMST**National Museum of Nature and Science, Tokyo, Japan (S. Nomura)

**NMV**Museum of Victoria, Melbourne, Victoria, Australia (K. Walker)

**NMW**Naturhistorisches Museum Wien, Vienna, Austria (M.A. Jäch)

**QDPIB**Queensland Department of Primary Industries, Brisbane, Queensland, Australia (C. Burwell)

**RMNH**Nationaal Natuurhistorisch Museum, Leiden, The Netherlands (M. Diekman)

**QM**Queensland Museum, Brisbane, Queensland, Australia (G. Monteith)

**SAMA**South Australian Museum, Adelaide, South Australia, Australia (C.H.S. Watts)

**SMTD** Staatliches Museum für Tierkunde, Dresden, Germany (O. Jäger)

**USNM**United States National Museum of Natural History, Smithsonian Institution, Washington, USA (W.E. Steiner)

**TDMB** Természettudományi Múzeum, Budapest, Hungary (O. Merkl †)

**WADA**Department of Agriculture, Western Australia, Perth, Australia (A. Szito)

**ZHMB**Museum für Naturkunde, Berlin, Germany (J. Frisch)

**ZSI**Zoological Survey of India, Kolkata, India (S. Sheela)

**ZSM**Zoologische Staatssammlung, München, Munich, Germany (M. Balke)

**ZRC**Zoological Reference Collection, Lee Kong Chian Natural History Museum National University of Singapore, Singapore (W.F. Hwang)

### ﻿Collecting procedures

Most of the *Sandracottus* obtained by the authors were collected using heavy aquatic dip nets or kitchen strainers. Diameters of meshes varied from 500 to 1000 mm. Leaf litter, rotten twigs or wood, and aquatic vegetation were swept heavily; the material obtained was then placed on a white nylon sheet (2 m^2^) or in a white plastic box. Specimens were sorted by hand. Referring to the label data, a few specimens of *Sandracottusbakewelliibakewellii* (Clark, 1864), *S.festivus* (Illiger, 1802), *S.femoralis* Heller, 1934, *S.hunteri* (Crotch, 1872) and *S.rotundus* Sharp, 1882 were obtained by operating light traps.

## ﻿Taxonomy and systematics

### ﻿Genus Sandracottus

Taxon classificationAnimaliaColeopteraDytiscidae

Sharp, 1882

07DCA20E-1E62-5BB3-B9BA-EF0F112A2B6F

#### Notes.

Medium-sized (11.9–16.6 mm) oval beetles, black or with contrasting black and yellow markings. Outer margin of metaventral wings arcuate not straight as in *Hydaticus* Leach, 1817. Metacoxal lines obliterated so that no supra-articular border present; mesofemur with longer ventral setae, at least some as long as half width of mesofemur. Spur on metatibia blunt, minutely emarginate at apex. A small genus of 11 species and one subspecies distributed in the Oriental and Australasian realms. The larvae of *S.dejeanii* (Aubé, 1898) was described by Vazirani (1971). All larval instars of *S.hunteri* and *S.femoralis* were described in detail by Alarie et al. (2023).

##### ﻿World check list of *Sandracottus*

***Sandracottusbakewelliibakewellii* (Clark, 1864)** Northern and coastal eastern Australia

***Sandracottusbakewelliiguttatus* (Sharp, 1882)** Central Australia

= Sandracottusrotundusab.reductus Zimmermann, 1926

***Sandracottusbizonatus* Régimbart, 1899** Malaysia: Sabah, Sarawak, Indonesia: Kalimantan

***Sandracottuschevrolati* (Aubé, 1838)** Indonesia: Lesser Sunda Islands, Timor, Tanimbar, Central and Southern Sulawesi

= *Sandracottusmixtus* (Blanchard, 1853) (syn. nov.)

***Sandracottusdejeanii* (Aubé, 1838)** India, Nepal, Pakistan, Myanmar, E Iran

= *Sandracottusvijayakumari*Anand et al. 2021 (syn. nov.)

***Sandracottusfemoralis* Heller, 1934** Indonesia: Moluccas, Irian Jaya; Papua New Guinea, Solomon Islands

= *Sandracottusflavocinctus* (Guérin-Méneville, 1830)

= *Sandracottusguerini* Balfour-Browne, 1939 (syn. nov.)

***Sandracottusfestivus* (Illiger, 1802)** China (?), India, Bhutan, Sri Lanka, Pakistan

***Sandracottushunteri* (Crotch, 1872)** India, Nepal, China, Korea, Vietnam, Laos, Myanmar, Thailand, Cambodia, West Malaysia, Indonesia: Sumatra, Java, Bali

= *Sandracottusfasciatus* (Fabricius, 1775)

= Sandracottusfasciatusvar.crucialis Régimbart, 1899

= *Sandracottusmanipurensis* Vazirani, 1969 (syn. nov.)

***Sandracottusinsignis* (Wehncke, 1876)** Philippines: Palawan, Luzon, Mindanao, Malaysia: Sabah (?)

= *Sandracottusbaeri* (Régimbart, 1877)

= *Sandracottusinsignisornatus* Sharp, 1882) (syn. nov.)

***Sandracottusjaechi* Wewalka & Vazirani, 1975** Sri Lanka: Nuwara Eliya

***Sandracottusmaculatus* (Wehncke, 1876)** Thailand, Vietnam, Laos, Cambodia, Malaysia: Sabah; Indonesia: Sumatra, Java, Kalimantan; Philippines: Mindanao

= *Sandracottuswehnckei* J. Balfour-Browne, 1944

= *Sandracottusangulifer* Heller, 1934 (syn. nov.)

= *Sandracottuspalawanensis* Sato, 1978 (syn. nov.)

= *Sandracottusnauticus* Sharp, 1882 (syn. nov.)

***Sandracottusrotundus* Sharp, 1882** Indonesia: Sulawesi

##### ﻿Descriptions

### ﻿ Sandracottus bakewellii bakewellii

Taxon classificationAnimaliaColeopteraDytiscidae

(Clark, 1864)

80843648-A793-5EAD-897D-CA2CD3DDFEC6

**Figures 1–3.**
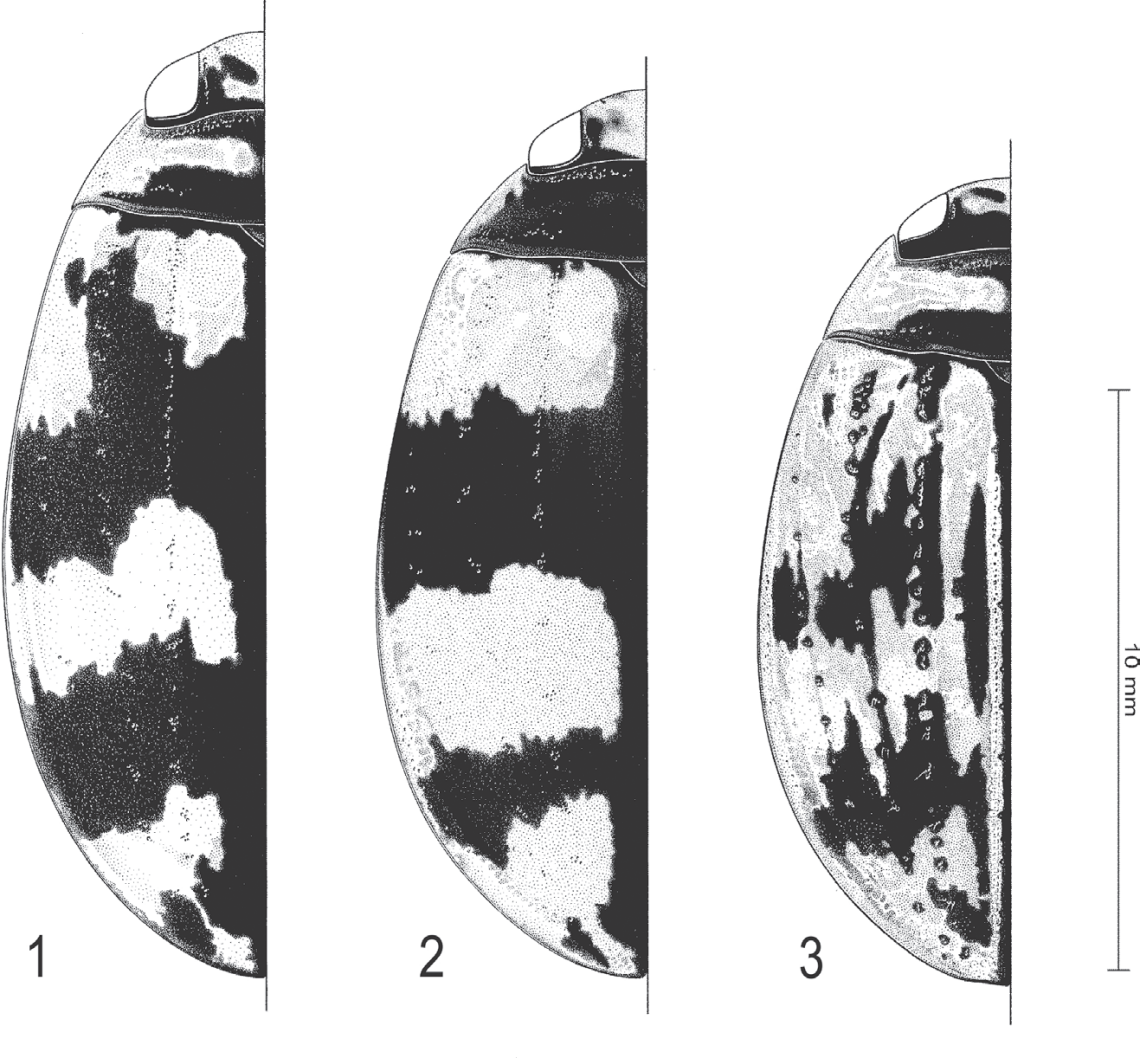
Habitus of **1***Sandracottusbakewelliibakewellii* (Northern Territory, Australia) **2***S.bizonatus* (Borneo, Sabah, Kinabalu) **3***S.chevrolati* (Sumba Island, Indonesia).

Figs 1 , 12 , 23 , 31 , 32 , 46

Hydaticus bakewellii Clark, 1864: 210 (type locality Moreton Bay, Queensland, Australia).
Sandracottus bakewellii (Clark, 1864): Sharp 1882: 687 (descr.); Régimbart 1899: 336 (descr.); Zimmermann 1920: 234 (cat.); Watts 1978: 148 (descr.); Watts 1985: 26 (cat.); Lawrence et al. 1987: 356 (cat.); Larson 1993: 59 (faun., ecol.); Larson 1997: 273 (faun., ecol.); Hendrich et al. 2019: 46 (faun., ecol., tax.); Hájek and Nilsson 2024: 91 (cat.).

#### Type material.

***Lectotype***: Male, “Lectotype”, “Type, 6756”, “Bakewelliii”, “Hydaticusbakewellii Clk. Det. C. Watts 1979” (NHMUK). ***Paralectotypes***: 1 female, “Moreton Bay”, “Sandracottusbakewellii Clk”, “Bakewellii Clark Moreton Bay”, “Syntype” (NHMUK); 1 male, “Moreton Bay”, “Bowring 6347*”, “Syntype” (NHMUK); 1 male, “Nova Holland.”, “Syntype” (NHMUK). Examined.

#### Additional material.

**(183 specimens)**: **Australia.** • **Northern Territory**: 1 ex., “N. Queensland Bloomfield River” (ZHMB); 2 ex., “Moreton Bay” (NHMUK) 1 ex., “Darwin, N.T., 1930” (MNHN); 1 ex., “Northern Austr.” (MNHN); 1 ex., “Northern Territory, F.E.Wilson coll.” (without further data) (VIC); 1 ex., “Tindal, 7.XI.1967, W.J.M. Vestjens leg.” (ANIC); 1 ex., “Northern Territory, A.H. Elston collection” (AM); 1 ex., “Burrells Creek, 17 miles S Adelaide River, 7.IV.1971, T. Weir leg.” (NTM); 1 ex., “Edith Falls, in pool, 23.VIII.1982, G. Allen & B. Russell leg.” (NTM); 1 ex., “Adelaide River Hills, 24 km N Robin Falls turn off, in pool, 17.X.1982, G. Husband leg.” (NTM); 3 exs., “Australia, Northern Territory, Katherine Gorge, Butterfly Gorge Walk, 150 m, 4.VII.1999, Hendrich leg., Loc. 33/133” (CLH); 1 ex., “Northern Territory, Burnside Stn., Brocks Crk., 25.VIII.1932, 25-018562, S 13.46.667 E 131.41.67, T.G. Campbell leg.” (ANIC); 1 ex., “Australia NT, 23 km S Adelaide River, permanent creek at Scenic Route, 137 m, 22.VIII.2006, 13.26.593S 131.11.012E, L. & E. Hendrich leg.” (NT 9) (ZSM). • **Queensland**: 1 ex., “Toowong District N. Queensland OE Janson sons” (USNM); 1 ex., “Rockhampton Sub. Distr. S.F.R. 5 Cpt. L.A. 17.VIII.1976 R.A. Yule Dept. For. Qld/ Acc. 1241/15”, “QFIC specimen incorporated into QDPC March 2010” (QDPIB); 1 ex., “Gayndah QLD 17.II.1963 H.A.Rose” (QM); 1 ex., “Mt. Glorious Q. 7.III.1959 K. Korboot” (QM); 1 ex., “Brisbane 6.X.1963 I.R. Bock” (QM); 7 exs., “Moolyamba Creek 9.V.1948 J.L.Wassell” (QM); 1 ex., “N Queensland, Cooktown, Eichhorn” (MNHN); 4 exs., “Rockhampton (Australie), Thoret 1870” (MNHN); 2 exs., “Dawson district, Barnard coll.” (MNHN, NMB); 2 exs., “Coll. French Queensland“ (MNHN, RMNH); 2 exs., “Hydaticusbakewellii Clk Type mihi D.S. [David Sharp] Queensland 983“ (NHMUK); 1 ex., “Bagot Creek” [SE Queensland, West of Dalby] (AUS); 1 ex., “Nova Holland Moreton Bay” (NHMUK); 1 ex., “Brisbane” (AM); 1 ex., “Brisbane Mt. Cootha 14.XI.1939 D 714” (QDPIB); 1 ex., “Brisbane” (NHMUK); 1 ex., “Rockhampton” (NHMUK); 2 exs., “Townsville” (MNHN); 2 exs., “Carnavon Gorge 26.I.1982 J.Sedlacek leg.” (NMB); 1 ex., “North Queensland Mutchilba A.D. Selby leg., F.E. Wilson coll.” (VIC); 1 ex., “Emu Creek pot hole 19.X.1940 J.Dewaney leg.” (VIC); 2 exs., “Rollingstone S.R.E. Brock Collection” (ANIC); 12 exs., “Bouldercome, 14.V.1966”, “A.N. C.G.L.Gooding Collection donated to ANIC 1979” (ANIC); 6 exs., “NW Queensland 28 N by E of Musselbrook Mining Camp Amphitheatre Springs S 18.21 E 138.11 12.V.1995 T. Weir leg.” (ANIC); 1 ex., “North Queensland Eungella NP Broken River 31.VIII.-8.IX.1998 Neave Edwards Powell Sutrisno & Hebbard leg.” (ANIC); 1 ex., “North Queensland 45 km N Aurukun 25.II.1981 M.Robinson leg.” (AM); 1 ex., “Mt. Carbine T.W.Gamble leg.” (AUS); 1 ex., “Queensland Carmila North coast Lancel. 1928 N. McArthur leg.” (AM); 2 exs., “Blackall River F. Witteron leg.” (QDPIB); 1 ex., “Highlands 4 km S Emmet, S 24°57'E 144°26´, 19.IV.2002, ex spring, sandstone gully, R.J.Felsham leg. (8951)” (QM); 4 exs., “N Queensland W. Cape York Peninsula Brown Creek pond VI.1982 P.Saenger leg.” (QM); 1 ex., “near Mt. Molloy, Rifle Creek 3.I.1990 ANZSES Expedition” (QM); 1 ex., “ME Queensland, Blackdown Tableland Stoney Creek via Dingo at light 17–19.XII.1985 S.Hamlet leg.” (QM); 1 ex., “N Queensland, White Mts. NP 2 km NE of RGSQ/AG Base Camp S 20.26 E 144.51 5–7.IV.2000, deep rocky pool, cloudy, sandy bottom, vegetation, half shade, T.Weir leg.” (ANIC); 2 exs., “N Queensland White Mts. NP, 53 km NE Prairie IX.1995, D.J.Cook leg.” (QM); 2 exs., “C Queensland Mt. Abbott summit area S 20°06´, E 147°45´, 750–1000 m, 8–10.XII.1996, G.Monteith & D.J.Cook leg.” (QM); 2 exs., “Watsonville 10 km W Herberton 9.XII.1990 D.J. Larson leg.” (ANIC, QM); 2 exs., “Brisbane 30.IV.1912 H. Hacker” (QM); 3 exs., “Sutton Collection, donation December 1964” (QM); 1 ex., “Queensland”, “coll. Felsche Geschenk 1907”, “Sandracottusbakewellii det. Gschwendtner” (SMTD); 1 ex., “near Collins, Catherine Creek, 20.XI.1990, T.Weir leg.” (ANIC); 1 ex., “Lake Mitchell 40 km N Mareeba 21.IX.1990 D.J.Larson leg.” (ANIC); 1 ex., “SE Mt. Carbine Saddle Bag Creek 15.XI.1990 D.J.Larson leg.” (ANIC); 3 exs., “Australia Queensland Atherton Tableland 30 km NNW Mareeba near Mitchell lake 9.XI.1996 Hendrich leg.” (CLH); 1 ex., “Julatten 11.IV.1984 at light, K.& E.Carnaby leg.” (ANIC); 1 ex., “North Queensland Julatten 2.IV.1977 Walford-Huggins leg.” (CLH); 1 ex., “Queensland Brisbane I. 1931” (CLH); 1 ex., “Mareeba Road, Clohesy River Road, I.1974, A. & M. Walford-Huggins” (CLH); 1 ex., “Hell Hole Gorge NP, S 25.34 E 144.11, X.1997, at light in open forest T.Weir leg.” (ANIC); 1 ex., “N Queensland, Coen River 1 km E of Rokeby Mungkan Kandju River, at light, S 13.39 E 142.41, 22.VII.1998, A.A.Calder leg.” (ANIC); 9 exs., “North Queensland, Cape Tribulation 26.-27.XII.1969 leg. G. Hangay” (CLH, TDMB); 1 ex., “Queensland”, “Coll. Franklin Müller”, “Sandracottusbakewellii det. A. Zimmermann” (DEI); 1 ex., “Queensland, Wallaroo, 17.I.1968, G. Hangay” (CLH); 12 exs., “Northern Queensland, Silver Valley, Samml. A. Zimmermann (ZSM); 1 ex., “Silver Valley N. Queensl. III”, “Coll. Gärtner”, “Sandracottusbakewellii Clark” (DEI); 2 exs., “Queensland”, “Coll. Franklin Müller” (ZSM); 1 ex., “Queensland Archer River 28.VII.1992 S 13.55 E 143.05 Zborowski, P. & Nielsen, E.S.” (ANIC); 1 ex., “Queensland, Brisbane (general), I.1931, S 25.01.856 E 27.46.667,153.0333, A Misko, S. leg.” (ANIC); 2 exs., “Queensland, Mary Creek, S 16.33 E 145.12.5 at light 4.XII.1968 E.B.Britton & S.Misko leg.” (ANIC); 1 ex., “Queensland Calliope River 23 km SE of Gladstone [approx. 23 km SW of Gladstone] 23.I.1970, at light, S 23.83.333 E 151.21.67 S.Misko leg.” (ANIC); 5 exs., “S QLD, 40 km E Bundaberg, Tusky Creek, 9 m, 26.IX.2006, 24.39.139S 152.01.477E, L. & E. Hendrich leg. (QLD 52)”; 5 exs., “S QLD, Winfield, Winfield Road, forest pool, 21 m, 26.IX.2006, 24.34.084S 152.00.513E, L. & E. Hendrich leg. (QLD 54)” (ZSM, CLH); 3 exs., “NH [= Neuholland] Coll. Plason” (NMW); 2 historical specimens leg. Bauer! [beginning of 19th century, without any detailed locality data, Jäch pers. comm. 2010]. • **Western Australia**: 1 ex., “Kununurra, Cave Springs, ex. pool 9.X.1966” (WADA); 3 exs., “Kimberely Research Station, 16.II.1959, K.T.Richards leg.” (WADA); 3 exs., “130 miles SE of Broome, 15.IX.1924, A.S.Cudmore leg.” (VIC); 2 exs., “Kimberley district N.V. Mjöberg”, “Samml. A. Zimmermann” (ZSM); 1 ex., “Australia occ.” [= Western Australia] Lea leg.”, “Sandracottusbakewellii Clark Dr. F. Guignot det.” (TDMB); 3 exs., “Western Australia, 163 km SE by E Broome 5.VIII.1976 Common I.F.B. leg.” (ANIC); 2 exs., “Western Australia, King Sd” [King Sound, area around Derby] (ANIC). • **Unknown states**: 1 ex., “Australia”, “Coll´n J.D. Sherman Jr. 1926” (USNM); 2 exs., “Australie” (MNHN); 1 ex., “Australia 7642” (NHMUK); 2 exs., “Australia Blackburns Collection”, “Sandracottusguttatus identified by L.A. Lea” (AUS); 4 exs., “Mootwingie 15.IX.195” (AUS). • **Doubtfull records**: 1 ex., “Victoria” (MNHN); 1 ex., “S. West Australia” (MNHN); 2 exs., “Museum Paris Tasmanie Verreaux 3-47” (MNHN).

#### Redescription.

Body broad oval, shiny testaceous with dark brown markings. Ventral side completely ferrugineus brown except testaceous fore and mid legs.

Head testaceous with posterior part broadly black; black part prolonged along eyes and protruding on frons. Surface shiny, very superficially shagreened. Punctation consisting of dense punctures, irregular in size and of larger and much sparser ones; these more numerous on frons. Clypeal grooves and punctures alongside eyes present, punctures medium-sized and coalescent. Antennae testaceous; antennomeres slender, antennomere V 5× as long as broad.

Pronotum testaceous with median black marking reaching from posterior to anterior margin; long and broad on posterior, distinctly shorter and narrower on anterior margin and strongly constricted in middle. Surface very slightly and superficially shagreened, with very dense punctation; punctures medium-sized not constant in size, distance 2–3× that of their diameter. Anterior rows of punctures interrupted in middle, punctures large and coalescent. Posterior row of punctures medially with large and coarsely impressed punctures in middle.

Elytra shiny, black, with larger basal, subbasal and apical elytral markings (Fig. 1). Epipleura ferrugineus brown. Surface distinctly shagreened and covered with small and dense punctures and with larger and much sparser ones. Puncture lines with groups of medium-sized punctures mostly grouped by five or six punctures; discal row almost complete. Sutural puncture line marked only by few punctures.

Ventral side dark brown. Fore and mid legs testaceous, hind legs ferrugineus brown to dark brown. Prosternal process short and broad, 1.6× longer than broad, finely but distinctly sculptured; posterior margin broadly rounded. Metatibia with sparse medium-sized to large punctures on outer proximal part. Ventrites II–VI very superficially shagreened, distinctly longitudinally wrinkled on whole lateral parts, densely covered with very small punctures and larger and sparser ones. Posterior margins rounded, deeply bordered with a row of large and coalescent punctures in middle of each side alongside margin.

**Figures 4–7.**
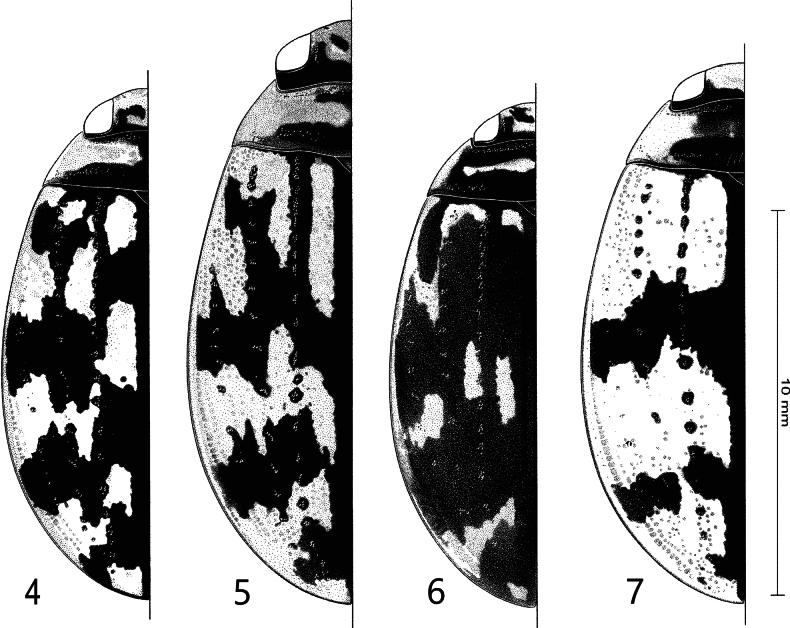
Habitus of **4***Sandracottusdejeanii* (South India) **5***S.festivus* (Sri Lanka) **6***S.femoralis* (Papua New Guinea, Rigo) **7***S.hunteri* (Thailand).

Measurements: TL = 13.5–14.5 mm, TL-h = 12.5–13.5 mm, TW = 8.0–9.0 mm.

♂. Protarsomeres I–III strongly enlarged with three larger suckers and numerous smaller one. Mesotarsomeres I–III with two rows of small suckers. Median lobe of aedeagus in ventral view broadened in apical third, then tapering towards apex (Fig. 12a). Parameres elongate and pointed at apex (Fig. 12b).

♀. Similar to male. Tarsomeres not enlarged. Microsculpture on ventrite VI as in male.

#### Differential diagnosis.

Colour of dorsal surface and shape of the median lobe distinguish *S.bakewellii* from all other species of the genus. It is the only species which occurs in Australia. The subspecies *S.bakewelliibakewellii* can be separated from *S.bakewelliiguttatus* by the broader and not interrupted yellowish anteromedian, posteromedian and preapical markings (Figs 46, 47).

#### Distribution.

Northern Australia, east coast from Northern Queensland south to Brisbane (Fig. 23). Specimens were collected from near sea level to 1000 m a.s.l.

**Figures 8–11.**
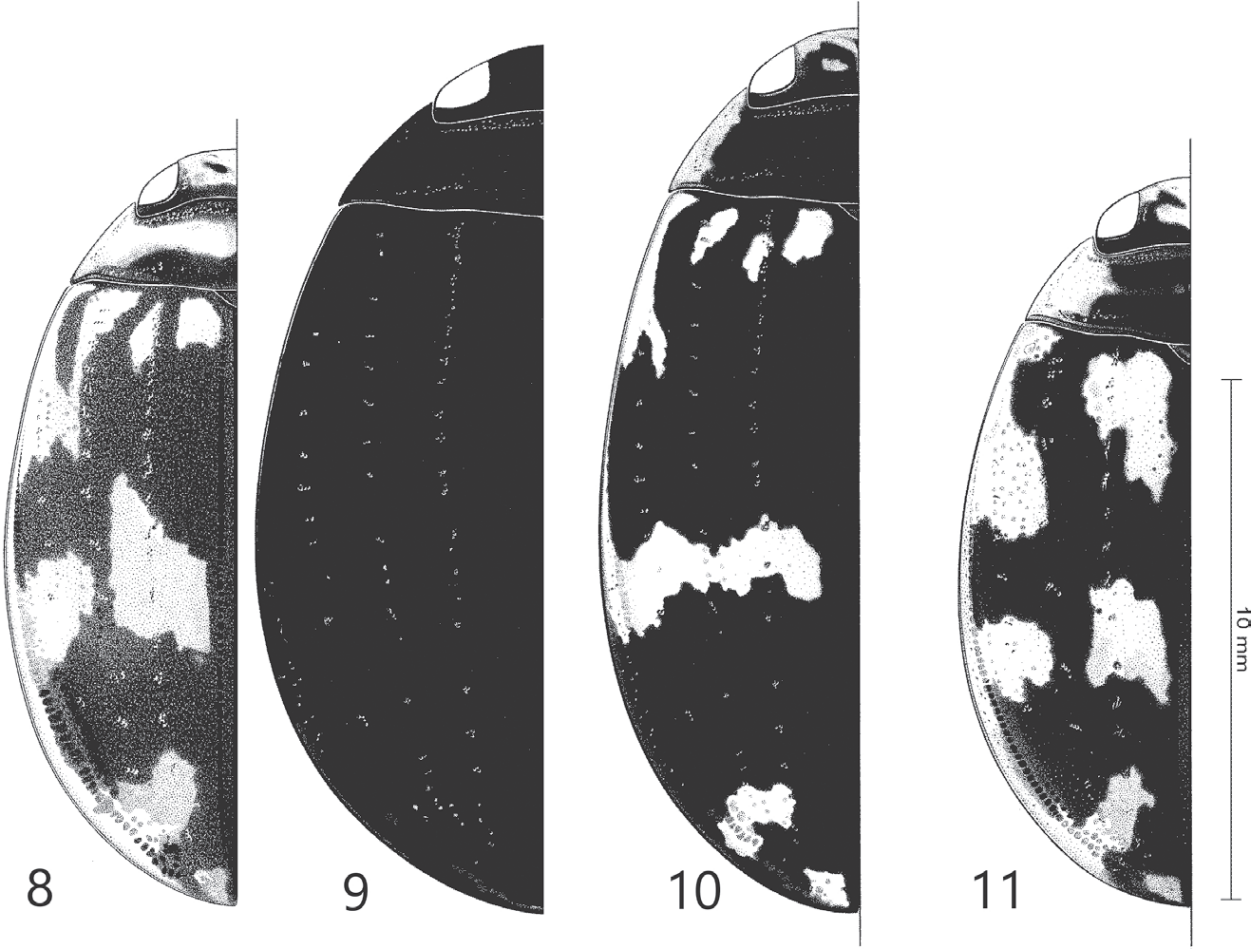
Habitus of **8***Sandracottusinsignis* (Luzon, Philippines) **9***S.jaechi* (paratype from Nuwara Eliya, Sri Lanka) **10***S.maculatus* (Thailand) **11***S.rotundus* (Sulawesi, Togian Islands).

#### Habitat.

*Sandracottusbakewellii* inhabits large (0.5 m depths or more) pools of seasonal streams and creeks, spring fed pools often with dark or gloomy water. The adults are generally found amongst tangles of roots and in places where the water is shaded. Habitats are often enriched with dead leaves and twigs (Figs 31, 32).

**Figures 12–15.**
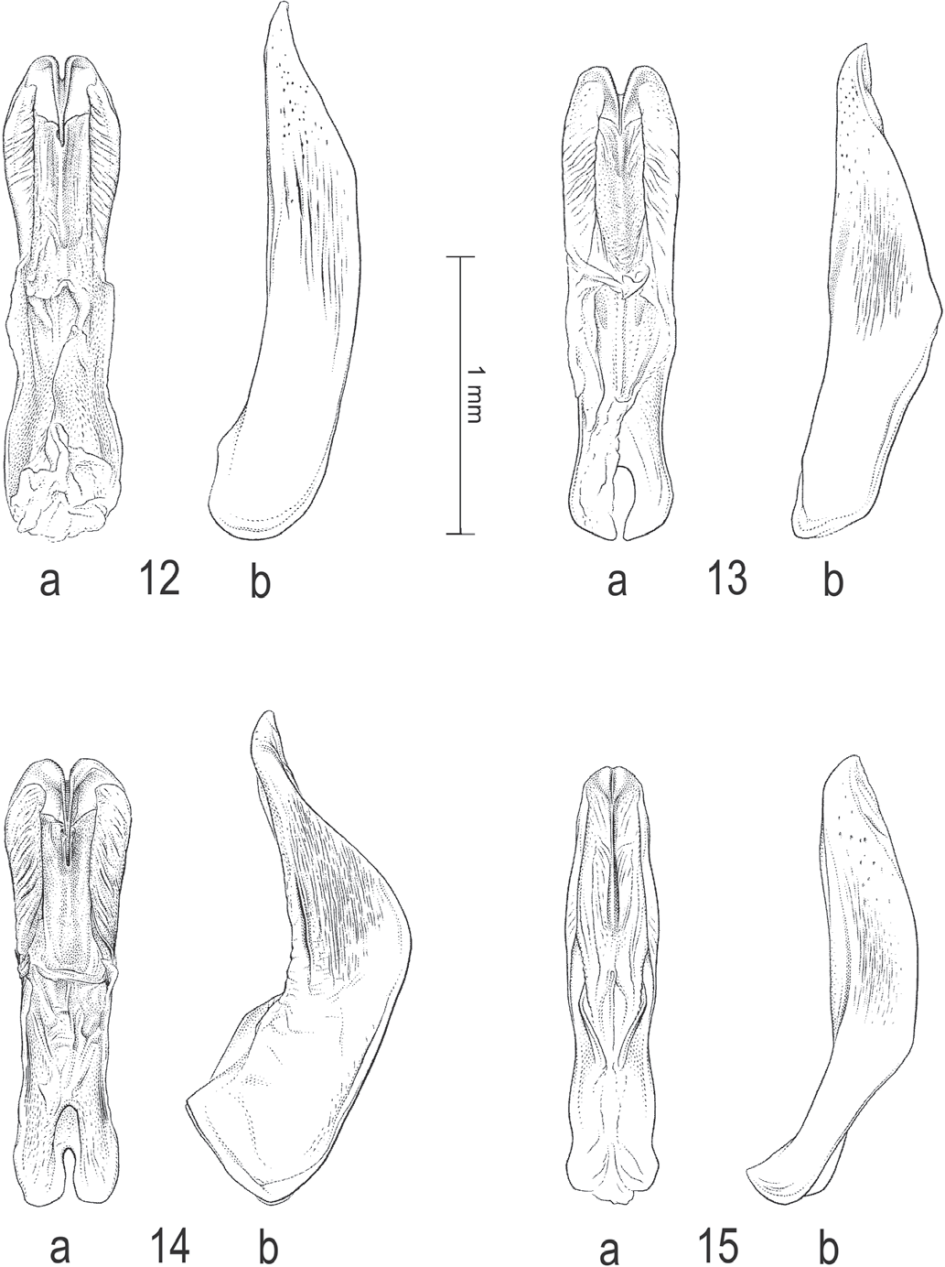
Median lobe of aedeagus in ventral view (a), and right paramere in lateral view (b) **12***Sandracottusbakewelliibakewellii***13***S.bizonatus***14***S.chevrolati* and **15***S.dejeanii*.

### ﻿ Sandracottus bakewellii guttatus

Taxon classificationAnimaliaColeopteraDytiscidae

Sharp, 1882 stat. nov.

DDD59582-F951-5799-9813-838156C6EE25

Figs 23 , 33 , 47

Sandracottus guttatus Sharp, 1882: 688 (type locality: Adelaide (?) [most probably mislabelled], South Australia, Australia); Régimbart 1899: 337 (descr.); Zimmermann 1920: 234 (cat.); Watts 1978: 148 (syn.); Hájek and Nilsson 2024: 91 (cat.).
Sandracottus rotundus ab. reductus Zimmermann, 1926: 97 (misidentification, infrasubspecific name).

#### Type material.

***Lectotype***: Male, “Australia ? Adelaide 984 guttatus”, “Lectotype”, “Sharp Coll. 1905-313.”, “Sandracottusguttatus Sharp Det. C.Watts 1979” (NHMUK). ***Paralectotype***: Female, “Carpentaria 984”, “Paralectotype”, “Sharp Coll. 1905-313.”, “Sandracottusguttatus Sharp Det. C.Watts 1979” (NHMUK). Examined.

Sandracottusrotundusab.reductus Zimmermann, 1926: 1 female, “Burg Station I 10.II.-16.III.1921, L.J.Toxopeus”, “Type”, “ab. reductus Zimmerm” [handwritten by Zimmermann] (ZSM). This specimen clearly belongs to *S.bakewelliiguttatus*. Toxopeus has collected it at a cattle station [Burg Station], somewhere in Central Australia. Examined.

#### Additional material.

**(63 specimens): Australia.** • **Northern Territory**: 1 ex., “N. Territory, S. Aust.”, “Coll. Kraatz” “Zimmermann det.” (DEI); 2 exs., “Moreton Bay” [doubtful record] (NHMUK); 1 exs., “Moreton Bay”, “guttatus Shp” [handwritten label by Régimbart but doubtful record] (MNHN); 2 exs., “Ormiston Gorge, X.1972, M. Baehr leg.” (ZSM, CLH); 6 exs., “Tallipatta Gorge, 20.VII.1947, C.W. Brazenov leg.” (VIC); 2 exs., “Hart Range C. Barrett leg., F.E. Wilson coll.” (VIC); 3 exs., “Central Australia Collection Horn Expedition, VII.1897” (VIC); 3 exs., “Illamurta Springs Conservation Reserve, small temporary rock pool, sandy bottom, S 24.19 E 132.41, 16.III.1995, T. Weir leg.” (ANIC); 1 ex., “Finke Gorge NP, temporary pools above old Ranger Station, 12.III.1995, T. Weir leg.” (ANIC); 2 exs., “Finke Gorge NP, gorge W of Finke River, permanent and temporary rock pools, algal growth and detritus, S 24.08 E 132.51, 15.III.1995, T. Weir leg.” (ANIC); 2 exs., “Finke Gorge NP, Palm Valley, small temp. pools, rocky, some sandy base, algal growth, detritus, S 24.03 E 132.43, 14.III.1995, T.Weir leg.” (ANIC); 3 exs., “45 km W of Alice Springs, Standley Chasm S 23.43 E 133.28 5.XI.1979, T.Weir leg.” (ANIC); 6 exs., “38 km SSE of Alice Springs S 24.01 E 134.01 7.XI.1979, T.Weir leg.” (ANIC); 1 ex., “60 km S of Alice Springs Ooraminna rockhole S 24.05 E 134.00 9.IV.1981 M. Malipatil & J. Hawkins leg.” (NTM); 1 ex., “60 km S of Alice Springs, Ooraminna rockhole, 24.05 S 134.00 E, 25.VII.1976, G. Griffin leg.” (NTM); 2 exs., “80 E of Alice Springs, Standley Chasm 26.III.1979 G. Griffin leg.” (NTM); 5 exs., “Alice Springs Old Huckitta Homestead, 20.VII.1970, D. Nelson leg.” (NTM); 2 exs., “Alice Springs, Valley of the Eagles, 14.II.1971 N.T.M.B. D. Nelson leg.” (NTM); 1 ex., “Tallaputta Gorge, 7.IX.1958” (NTM); 1 ex., “Kings Canyon George Gill Range 25.-26.III.1983 at light I. Archibald leg.” (NTM); 3 exs., “Kings Canyon, George Gill Range 24.V.2006 C.H.S. Watts leg.” (ZSM, SAMA); 2 exs., “Northern Territory, nr. Reedy Rockhole Amadeus Basin 12.IX.1962 25-018565, -24.33333,131.5833, P. Ranford leg.” (ANIC); 5 exs., “Northern Territory Standley Chasm 43 km W by S of Alice Springs, 11.X.1972, 25-018569,-23.71667, 133.4667 M.S. Upton leg.” (ANIC). • **Western Australia**: 12 exs., “Rawlinson Range 22.VII.1967 K.J. Richards leg.” (WADA).

**Figures 16–19.**
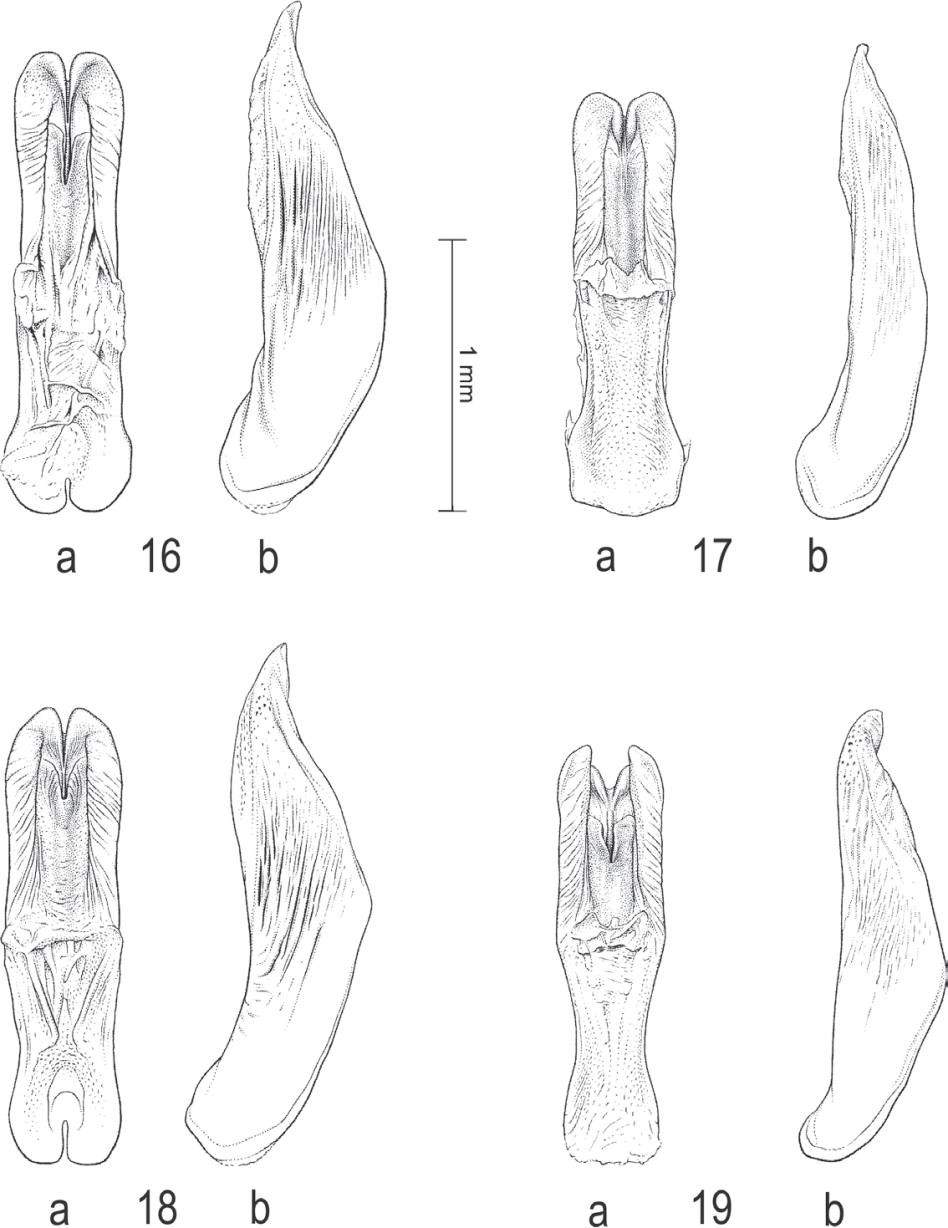
Median lobe of aedeagus in ventral view (a), and right paramere in lateral view (b): **16***Sandracottusfestivus***17***S.femoralis***18***S.hunteri* and **19***S.insignis*.

#### Comments on classification.

Specimens with reduced yellow basal, subbasal and apical elytral markings or almost completely black elytra and pronotum (Fig. 47) have been described as *Sandracottusguttatus* Sharp, 1882 but were later synonymised with *S.bakewellii* by Watts (1978). Despite the fact that they are genetically (cox 1) and morphologically identical with specimens from coastal northern and eastern Australia, they have a very restricted distribution in the ranges and gorges of Central Australia, and no intermediate forms are known so far. We propose subspecific rank for the population from Central Australia. The form described by Zimmermann as “ab. reductus” also refers to such a dark specimen with reduced yellow elytral markings.

#### Distribution.

Central Australia (e.g., Macdonnell Ranges, Finke Gorge, Rawlinson Range) (Fig. 23).

#### Differential diagnosis.

The subspecies *S.bakewelliiguttatus* can be separated from *S.bakewelliibakewellii* by less expanded and interrupted yellowish antemedian, postmedian and preapical yellow markings on elytra (Figs 46, 47).

#### Habitat.

*Sandracottusbakewelliiguttatus* inhabit more or less permanent pools of seasonal streams and creeks, and spring fed pools. The adults are generally found in places where the water is shaded. Habitats are often enriched with dead leaves and twigs (Fig. 33).

#### Conservation.

An isolated subspecies with a very small range which needs special conservation attention as surface water in this area is very limited.

### ﻿ Sandracottus bizonatus

Taxon classificationAnimaliaColeopteraDytiscidae

Régimbart, 1899

A88F9289-A45B-52BE-B532-6BFDB80550D2

Figs 2 , 13 , 24 , 48

Sandracottus bizonatus Régimbart, 1899a: 336 (type locality Malaysia, Sabah); Zimmermann 1920: 234 (cat.); Hájek and Nilsson 2024: 91 (cat.).

#### Type material.

***Lectotype*** (herewith designated): Male, “Borneo Sandakan Windrath” [white printed label], “bizonatus Reg.” [handwritten label by Régimbart], “Lectotype Sandracottusbizonatus Régimbart Hendrich & Brancucci des.” [red printed label] (MNHN). Examined.

**Figures 20–22.**
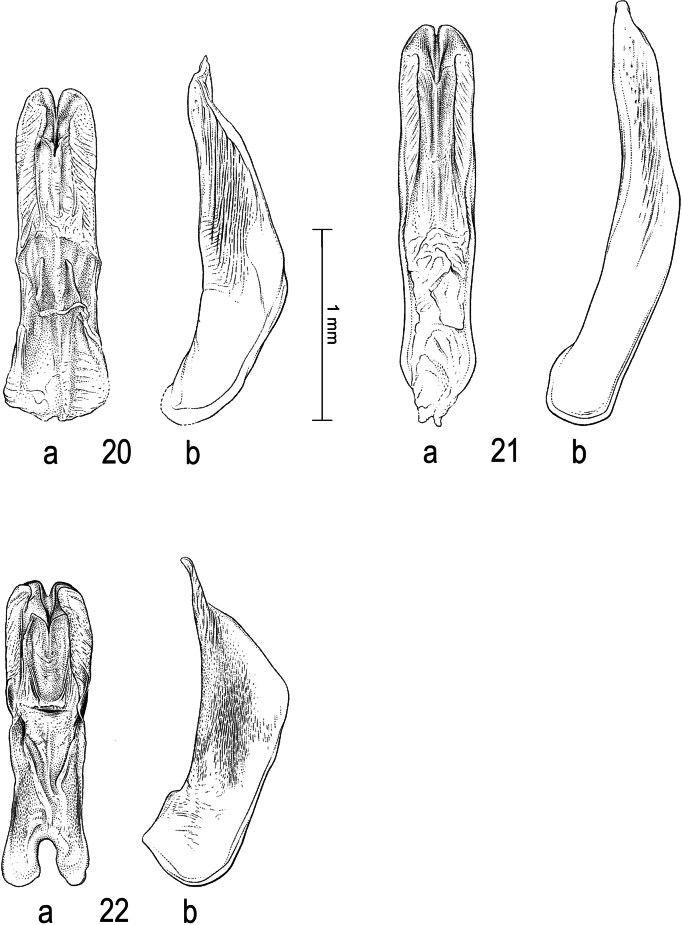
Median lobe of aedeagus in ventral view (a), and right paramere in lateral view (b): **20***Sandracottusjaechi***21***S.maculatus* and **22***S.rotundus*.

#### Additional material.

**(45 specimens)**: **Malaysia.** • **Sabah**: 1 ex., “Borneo, Sabah Ranau, 4.v.2006 Steven Chew NHMUK 2006-36” (NHMUK); 1 ex., “E. Malaysia, Sabah Borneo, Mt. Trus March-April 2010 Local leg.”, “coll. A. Skale Hof/Germany” (CAS); 5 exs., “Nord Borneo Mt. Kina-Balu 5.VIII.1903 John Waterstradth” (MNHN); 2 ex., “Kinabalu Borneo” (MNHN); 1 ex., “N. Borneo, Kinabalu” (MNHN); 8 exs., “v.d. Does de Beye, Malakka” (RMNH); 1 ex., “Borneo, H.E. Andrewes Bequest. B.M. 1922-221” (NHMUK); 1 ex., “Kina Balu” (MNHN); 2 exs., ”N. Borneo Mt. Kina Balu 5.VIII.1903 J.Waterstradt leg.” (MNHN); 1 ex., “Borneo, Sabah Mt. Kinabalu 3000f, 21.IV.1929 ex H.M. Pendlbury Collection” (CGW); 1 ex., “Borneo Kinabalu 1500 m H.Bolle Berlin SW11” (MNHN); 1 ex., “Borneo, Sabah, Tibow, 45 km NE of Sapulut, 600-900 m, 7.-15.IV.2000, Bolm leg.” (NMB); 1 ex., “Borneo, Sabah, Kampung Pisang, Pisang env., tributary of Kuamut river, 29.VI.1998, J.Kodada & F.Ciampor leg.” (NMW); 1 ex., “Sabah, Borneo env. Keningau V.1993” (CLH); 2 exs., “Nord Borneo”, “Samml. A. Zimmermann” (ZSM); 2 exs., “Kinabalu Nord Borneo”, “Samml. A. Zimmermann” (ZSM); 5 exs., “Kinabalu Borneo 1500 m”, “Samml. A. Zimmermann” (ZSM); 1 ex., “Nordost Borneo Gebrüder W. Müller Vermächtnis 1909” (SMTD); 1 ex., without locality label, “*Sandracottusbizonatus* Gschwendtner det.” (SMTD). • **Sarawak**: 1 ex., “Sarawak, Kapit distr., Rumah Ugap vill., Sut river, 3.-9.III.1994, J.Horák leg.” (NMW); 4 exs., “Sarawak, Bario env., Pa Ukat, 24.VI. 2003, J. Šťastný lgt.” (CJS); 2 exs., “Sarawak, Kelabit, Bario env. 21.-25.VI.2003, J. Šťastný lgt.” (CJS).

#### Redescription.

Body broad oval, shiny, reddish brown with broad black markings on elytra (Figs 2, 48). Ventral side completely dark brown to black, legs testaceous, hind legs somewhat darker.

Head ferrugineus with posterior part broadly black, shiny. Black band protruding forwards to frons. Surface almost smooth consisting of dense and very numerous punctures of different sizes and of larger, much sparser ones, particularly numerous on frons. Clypeal grooves, punctures alongside eyes and transverse depressions beside eyes distinctly impressed, punctures large and coalescent. Antennae ferrugineus; antennomeres slender, fifth 4× as long as broad.

Pronotum ferrugineus with large median black marking reaching from posterior to anterior margins (Figs 2, 48). Surface shagreened, with dense punctation; punctures medium-sized mixed with smaller ones. Anterior and lateral puncture rows dense and coalescent, punctures becoming sparse towards middle and lacking in very middle of anterior margin. Posterior row of punctures with coarse and coalescent punctures in middle of each side, distinctly smaller and spaced on disc.

Elytra ferrugineus brown with black and broad markings consisting of three transverse bands (Figs 2, 48); an antemedian one, a postmedian one and a preapical one. Epipleura ferrugineus brown. Surface of elytra very slightly and superficially shagreened and with double punctation, a smaller and dense punctation as well as a larger one much more sparsely distributed. Row of punctures with groups of medium-sized punctures mostly grouped in five or six punctures, groups closer together on discal row.

**Figure 23.**
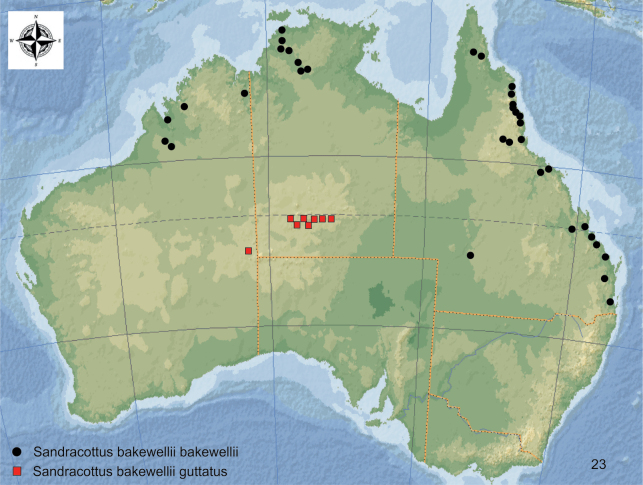
Distribution of *Sandracottusbakewelliibakewellii* [black dots] and *S.bakewelliiguttatus* [red squares] in Australia.

Ventral side dark brown. Fore and mid legs particularly testaceous, hind legs ferrugineus brown to dark brown. Prosternal process short and broad, 1.5× longer than broad, flattened finely but distinctly sculptured; posterior margin broadly rounded. Whole surface very superficially shagreened and finely punctured. Metatibia with sparse medium-sized punctures on outer half. Ventrites II–VI very superficially shagreened, slightly and longitudinally wrinkled on lateral parts, on whole surface densely covered with very small punctures and larger and sparser ones. Posterior margins rounded, bordered with some large and coalescent punctures in middle of each side.

Measurements: TL = 12.5–13.0 mm, TL-h = 11.4–12.2 mm, TW = 8.5–9.0 mm.

♂. Protarsomeres I–III strongly enlarged with three larger suckers and numerous smaller one. Mesotarsomeres I–III with two rows of small suckers. Median lobe of aedeagus, in ventral view, broad, parallel-sided up to apex where it is slightly broadened and broadly rounded (Fig. 13a). Parameres broad, same length as median lobe, and pointed at apex (Fig. 13b).

♀. Similar to male, tarsi not enlarged. Microsculpture on ventrite VI as in male.

#### Differential diagnosis.

The combination of dorsal colour pattern (Figs 2, 48) and shape of median lobe of aedeagus and parameres (Fig. 13a, b) separates *S.bizonatus* from all other species of the genus. The species is endemic to Borneo and co-occurs with *S.maculatus*.

#### Distribution.

**Figure 24.**
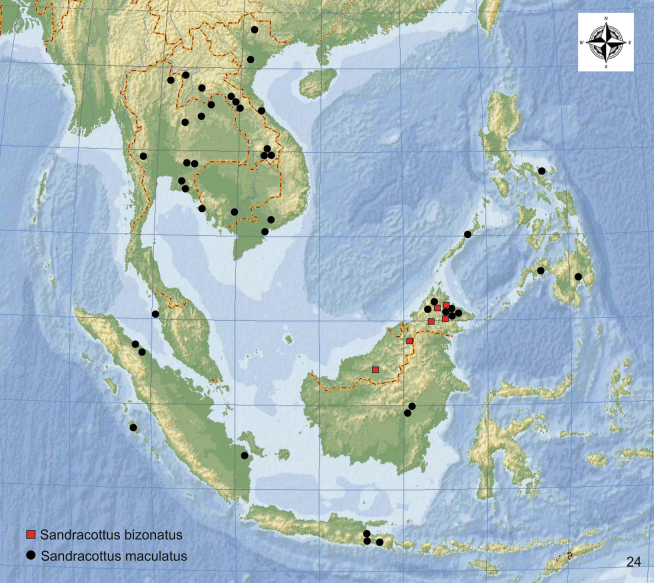
Distribution of *Sandracottusbizonatus* [red squares] and *S.maculatus* [black dots] in Southeast Asia.

Malaysia (Sabah, Sarawak) (Fig. 24). The eight specimens deposited in RMNH from Malacca are most probably mislabelled. According to a photo on a website from an insect dealer in Indonesia, the species was also collected in Kalimantan (https://www.giradis-insect.com/ 27.9.2024). Specimens were collected between 300 and 1.500 m a.s.l.

#### Habitat.

Muddy and shaded forest pools, rich in rotten leaves and more permanent side pools of larger forest streams in primary rainforests, partly shaded and enriched with rotten leaves and twigs (J. Kodada and J. Šťastný, pers. comm. March 2013).

#### Conservation.

A rare and highly endemic species of Borneo, probably associated with the declining primary lowland and hilly rainforests on the island (see Southeast Asian species *S.femoralis*, *S.insignis*, *S.maculatus* and *S.rotundus*). Most records are from the end of the 19^th^ and the beginning of the 20^th^ century. According to present knowledge it is an endangered species. It is recommended to be listed in the next IUCN red list.

### ﻿ Sandracottus chevrolati

Taxon classificationAnimaliaColeopteraDytiscidae

(Aubé, 1838)

E4C77047-832F-5568-A2A8-7340E0A05492

**Figure 25.**
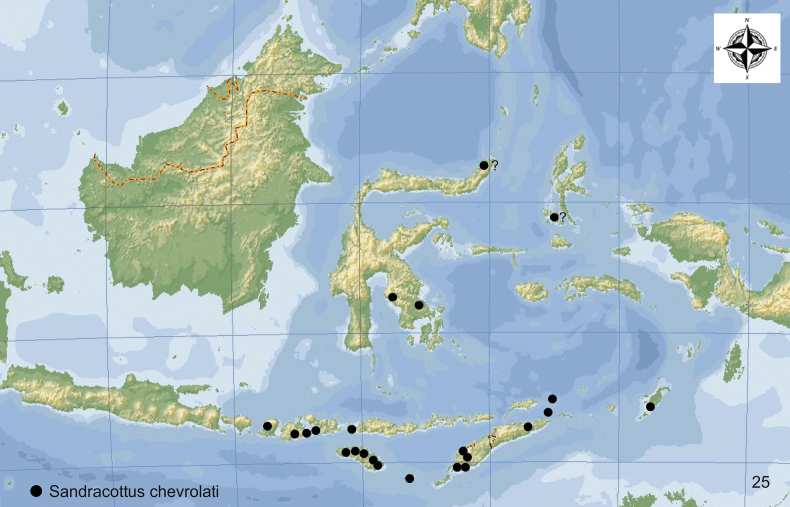
Distribution of *Sandracottuschevrolati* in Indonesia.

Figs 3 , 14 , 25 , 49

Hydaticus chevrolati Aubé, 1838: 164 (type locality Timor, Indonesia).
Sandracottus chevrolati (Aubé, 1838): Régimbart 1899: 334 (descr.); Zimmermann 1920: 234 (cat.); Hájek and Nilsson 2024: 91 (cat.).
Hydaticus mixtus Blanchard, 1843: plate 4, fig. 2 (type locality Timor, Indonesia) (syn. nov.).

#### Comments on classification.

The verbal description of *H.mixtus* was published by Blanchard (1853). However, before the publication of the text, the colour plates to this book were published separately during the years 1842–1854 (see Clark and Crosnier 2000). Therefore, the plates must be considered as the original description (ICZN 1999: Article 12.2.7). *Sandracottusmixtus* was regarded as a junior subjective synonym of *Dytiscusfasciatus* Fabricius, 1775 (= primary homonym of *Dytiscusfasciatus* DeGeer, 1774) by Vazirani (1969). However, Vazirani mentioned that he had no possibility to see the type or the original description of that species. Although we did not see the type specimen of *H.mixtus*, the figure which serves as the original description is very accurate and refers without any doubt to the species described by Aubé (1838) under the name *H.chevrolati*. In addition, the type locality of both species is the same (Timor), and we are not aware of any records of *Sandracottusfasciatus* [= *hunteri*] east of the Wallace’s line. Therefore, we consider *Hydaticusmixtus* as a junior subjective synonym of *H.chevrolati*.

#### Type material of *Hydaticuschevrolati*.

***Lectotype*** (herewith designated): Male, “Timor” [handwritten label], “typus” [handwritten label] “Lectotype Hydaticuschevrolati Aubé des. L. Hendrich & M. Brancucci 2010” [red printed label] (MNHN). ***Paralectotype***: Female, “Timor” [handwritten label], “Paralectotype Hydaticuschevrolati Aubé des. L. Hendrich & M. Brancucci 2010” [red printed label] (MNHN). Examined.

#### Type material of *S.mixtus*.

Not examined.

#### Additional material.

**(157 specimens)**: **Indonesia**: • **Lombok**: 1 ex., “Indonesia: N Lombok, Senaru waterfall, alt. m. 0470, 13.x.1991, Huijbregts, Krikken” (RMNH). • **Pantar**: 2 exs., “Indonesia, Lesser Sundas, Pantar Isl, east coast, Tanah Labnag env., 350 m, S.Jakl leg. 9.-21.iii.2006” (NMPC). • **Sumba**: 7 exs., “O. Sumba Prai Jawang Rende Wai 14.7.1949 Dr. Bühler Dr. Sutter” (NMB); 1 ex., “W. Sumba Mata Kori Waimangura 23.8.1949 Dr. Bühler Dr. Sutter” (NMB); 1 ex., “W. Sumba Baing 30.6.1949 Dr. Bühler Dr. Sutter” (NMB); 1 ex., “O. Sumba Mau Maru 18.7.1943 Dr. Bühler Dr. Sutter” (NMB); 1 ex., “O. Sumba Kodi 8.8.1949 Dr. Bühler Dr. Sutter” (NMB); 1 ex., “O. Sumba Melolo 28.Mai 1949 Dr. Bühler-Dr. Sutter” (NMB); 1 ex., “Indonesia, Lesser Sundas Sumba East, 10–20.6.2006, Luku Melolo N.R., 300–500 m, S. Jakl leg.” (CLH); 2 exs., “Sumba East, Melolo env., Luku Melcio, 300–600 m, 7.-9.II.2001, P. Votruba leg.” (CJS); 2 exs., “Sumba Island mer., Tarimbang env., 0–100 m, 2.II.-3.II.2001, S.Jákl leg.” (NMPC); 2 exs., “East Sumba 550 m, Luku Melolo N.R. 1.-10.7.2005” (CLH); 1 ex., “Sumba Island or., Mt. Wangameti, Kanangar env., 600–800 m, 10.II.2001, S. Jákl leg.” (NMPC); 11 exs., “Indonesia, East Sumba, 20 km S of Waingapu Wairinding, 300 m, 30.I.-2.II.2001, P.Votruba leg.” (CLH, NMPC). • **Sumbawa**: 33 exs., “Sumbawa, Dompu-Empang, restpools, 90 m, 15.ix.2011, 08 35.563S 118 17.454E (SUMB06)” (MZB, ZSM); 1 ex., “Sumbawa” (MNHN); 2 exs., “Savu I. [=Savu Island] Viii 96 Hvereti” (MNHN); 3 exs, “Museum Paris Coupang Hombron 1841” (MNHN); 7 exs., “Sumbawa Colffs.”, “Ex. Musaeo Van Landsberge” (MNHN); 1 ex., “Sumbawa” (MNHN); 2 exs., “v.Lansb., Sumbawa” (RMNH); 1 ex., “Sumbawa, 56993, Fry Coll. 1905-100” (NHMUK); 2 exs., “Sumbawa” (MNHN); 1 ex., “Indonesia, W-Sumbawa, Batoe Doelang, 10.-15.V.1927, B.Rensch” (CGW). • **Flores**: 2 exs., “Indonesia, Flores, Rinca, V.1990, C.H.S. Watts leg.” (CLH, SAMA). • **Tanimbar**: 6 exs., “Indonesia, Tanimbar Yamdema isl., Lorulun, 20 km NE of Saumlaki, 150 m, 26.11.-4.12.2006, M Oboril lgt.” (CLH, ZSM); 2 exs., “Indonesia, Tanimbar-Yamdena, Mams, 21 km N of Saumlaki, 27.XI.-11.XII.2005, J.Horák leg.” (NMPC); 13 exs., “Indonesia, Tanimbar isls, Lorulun, 20 km NE of Saumlaki, 150 m, 25.11.-24.12.2006, St Jakl lgt.” (CLH, ZSM). • **Timor**: 1 ex., “Indonesia, West Timor Buraen env. 28 km S Kupang 400 m, 16.-21.12.2005 St. Jakl leg.” (CLH); 1 ex., “TIMOR ISL Buraen env. 60 km SE Kupang 350 m, 10.-21.2.2006 St. Jakl leg.” (CLH); 4 exs., “Timor, Naikliu area, restpools in dry forest, 130 m, 3.x.2011, 09 58.425S 123 41.439E (TIM11)” (MZB, ZSM); 1 ex., “Timor, alte Sammlung” (ZSM); 1 ex., “Timor” (RMNH); 6 exs., “Macklot, Timor” (RMNH); 3 exs., “Dr. H. ten Kate, Timor centr.” (RMNH); 4 exs., “S.O.Celebes, J. Elbert, 1909” (RMNH); 1 ex., “W.P. de Roever, Nenas en omgeving, Moetisgebergte Timor, Sept. 1937” (RMNH); 6 exs., “Tim. [Timor] 6756” (NHMUK); 1 ex., “Tim. [Timor], Wallace, Celebis (Hydaticus) MS Clark” (NHMUK); 1 ex., “Timor 978 var., Sharp Coll. 1905-313” (NHMUK); 2 exs., “Timor, Lelogama, Mai 1911, Haniel”, “Samml. A. Zimmermann“ (ZSM); 1 ex., “Timor”, “Coll. C. Felsche Geschenk 1907” (SMTD). • **Sulawesi**: 2 exs., “Manado, Celebes [Sulawesi] 2014” (NHMUK); 1 ex., “S.O. Celebes T. Elbert 1909”, “Rambi bis Mengkoka”, “ex Museum Buitenzorg”, “Sandracottuschevrolati Aubé det. A. Zimmermann” (DEI); 2 exs., “Celebes [Sulawesi] Posso [Poso] See Drs. Sarasin” (NMB, SMTD); 1 ex., “SO Sulawesi, I. Albert”, “Samml. A. Zimmermann” (ZSM). • **Moluccas**: 1 ex., “Moluccas” (MNHN); 2 exs., “Moluccas Batchian [most probably mislabeled] J. Waterstradt 1902” (MNHN); 1 ex., “Rotti Carl Auriv.”, “Samml. A. Zimmermann” (ZSM); 2 exs., “Kisser Inseln Mai 1901” [Pulau Kisar], “Samml. A. Zimmermann” (ZSM); 2 exs., “Molukken Ins. Roma” [Pulau Romang], “Samml. A. Zimmermann” (ZSM). • **Timor-Leste**: 1 ex., “Timor-Leste, 6.ii.2011, Ossu, Mundo Perdido Mts. 945 m, 8°42'47.6"S, 12°49'29"E” (NMPC).

#### Redescription.

Body broad oval, shiny, testaceous with black markings. Ventral side completely dark brown to black, legs testaceous, hind legs somewhat darker (Figs 3, 49).

Head testaceous with posterior part and broadly so on posterior half alongside as well as two elongate spots on clypeus black, shiny (Figs 3, 49). Punctation consisting of dense and very numerous punctures of different sizes and of larger, much sparser ones, particularly numerous on frons. Clypeal grooves, punctures alongside eyes and a transverse depression beside eyes distinctly impressed, punctures large and coalescent. Antennae testaceous; antennomeres slender, antennomere V 4.5× as long as broad.

Pronotum testaceous with a median black marking reaching from posterior to anterior margins; long and broad on posterior, distinctly shorter and narrower on anterior margin and strongly constricted in middle (Figs 3, 49). Surface very superficially shagreened, almost not discernible, with dense punctation; punctures medium-sized mixed with smaller ones. Anterior and lateral puncture lines dense and coalescent, punctures becoming sparse towards middle and lacking in very middle of anterior margin. Posterior puncture line with coarse and coalescent punctures in middle of each side, distinctly smaller and spaced on disc.

Elytra testaceous to ferrugineus brown with black markings consisting of three transverse bands; particularly characterised by the presence of longitudinal testaceous subsutural spots (Figs 3, 49). Epipleura testaceous to ferrugineus brown. Surface very slightly and superficially shagreened and covered with a double punctation, a smaller and dense one as well as a larger one more sparse. Puncture lines with groups of medium-sized punctures mostly grouped in five or six punctures, groups closer together on discal row.

Ventral side dark brown. Legs, particularly fore and mid legs testaceous, hind legs ferrugineus brown to dark brown. Prosternal process short and broad, 1.5× longer than broad, flattened finely but distinctly sculptured; posterior margin broadly rounded. Whole surface very superficially shagreened and finely punctured. Metatibia with sparse medium-sized punctures on outer half. Ventrites II–VI very superficially shagreened, slightly and longitudinally wrinkled on lateral parts, whole surface densely covered with very small punctures, with sparser larger ones. Posterior margins rounded, bordered with some large and coalescent punctures on the middle of each side.

Measurements: TL = 13.0–13.5 mm, TL-h = 11.2–12.4 mm, TW = 8.0–8.5 mm.

♂. Protarsomeres I–III strongly enlarged with three larger suckers and numerous smaller one. Mesotarsomeres I–III with two rows of small suckers. Median lobe of aedeagus, in ventral view, broadened on apical third, then tapered up to apex, here broadly rounded (Fig. 14a). Parameres basally broad and pointed at apex (Fig. 14b).

♀. Similar to male, tarsi not enlarged. Microsculpture on ventrite VI as in male.

#### Differential diagnosis.

The dorsal colour pattern of the Indonesian *S.chevrolati* is near to the Indian *S.festivus* but *S.chevrolati* can be easily separated by its smaller size (TL = 10–13.5 mm, *S.festivus*: TL = 14.7–15.5 mm) (Figs 3, 49), its distributional range (Figs 25, 29), and shape of median lobe and parameres (Figs 14a, b, 16a, b).

#### Distribution.

Indonesia: Lesser Sunda Islands east of the Wallace Line (Lombok, Sumba, Sumbawa, Flores), Tanimbar, Timor, south-eastern Sulawesi, and southern Moluccas (Pulau Romang) (Fig. 25). Old records from northern Sulawesi and Bacan Island need to be confirmed. Specimens were collected between 90 and 800 m a.s.l.

#### Habitat.

The specimens from Rinca (Flores) and Sumbawa were collected in rest pools of an almost dry stream bed, partly shaded by monsoonal rainforest. The bottom consisted of rocks and coarse sand, covered with rotten leaves (C.H.S. Watts and M. Balke pers. comm. 2010).

### ﻿ Sandracottus dejeanii

Taxon classificationAnimaliaColeopteraDytiscidae

(Aubé, 1838)

CAB6115E-A178-522C-9F79-9C501485A93B

**Figure 26.**
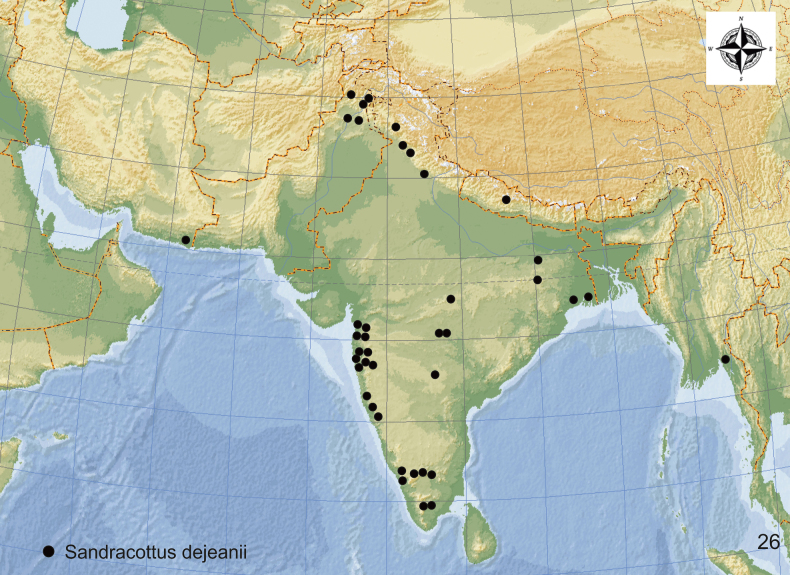
Distribution of *Sandracottusdejeanii* in Asia.

Figs 4 , 15 , 26 , 34 , 35 , 50

Hydaticus dejeanii Aubé, 1838: 165 (type locality “Indes Orientales”).
Sandracottus dejeani (Aubé, 1838) (sic.): Sharp, 1882: 686 (comb. nov.); Régimbart 1899: 335 (descr.); Zimmermann 1920: 234 (cat.); Guéorguiev 1965: 111 (faun.); Vazirani 1969: 275 (descr., cat.); Vazirani 1971: 25 (cat.); Sonali et al. 2022: 339 (faun.).
Sandracottus dejeanii (Aubé, 1838): Hájek 2006: 50 (faun.); Ghosh and Nilsson 2012: 18 (cat.); Ghosh 2015a: 81 (faun.); Ghosh 2015b: 77 (faun.); Shangar et al. 2023: 455 (faun.); Deb and Subramanian 2023: 14 (faun.); Hájek and Nilsson 2024: 91 (cat.); Shaverdo et al. 2024: 38 (check list, faun.); Sheth et al. 2024: 10, 20 (check list, faun., key).
Sandracottus vijayakumari Anand et al., 2021: 17999–18003 (type locality Western Ghats, Kerala, India) (syn. nov.).

#### Type material of *Hydaticusdejeanii*.

Not found (MNHN).

#### Type material of *Sandracottusvijayakumari*.

Not examined.

#### Additional material.

**(143 specimens)**: • **India**: 2 exs., “Mahableshnar W. Ghato, 5500ft India”, “Coll´n J.D. Sherman Jr. 1926” (USNM); 2 exs., “Aug. 43 Dehra Dun” [Prov. Uttarakand, 1943, Dehradun, Central Internment Camp for British India near Premnagar, 30°20 N 78°3 E] (ZSM); 1 exs., “Okt. 43 Dehra Dun” (ZSM); 1 ex., “Inde méridionale M. Moingeon” (NMB); 4 exs., “India, Batate, Patnitop 1600–2100 m 6.-8.8.1980 W. Heinz leg.” (NMB); 1 ex., “India Hydarabad M. Halva leg.” (NMB); 2 exs., “Chota Nagpore Nowatoli R.P.Cardon VII-IX.1888” (NMB, MNHN); 2 exs., “Puna”, “Coll. Kraatz”, “Zimmermann det.” (DEI); 23 exs., “Inde Anglaise, Kalka, ex. Coll. Oberthür” (MNHN); 32 exs., “Chota-Nagpore Nowatoli R.P. Cardon VIII-IX.1896” (MNHN); 4 exs., “Chota Nagpore, Nowafoli, R.P.Cardon, VIII-IX 1898” (RMNH); 4 exs., “Chota-Nagpore Nowatoli, R.P. Cardon IX-X.1896” (MNHN); 1 ex., “Chota-Nagpore Nowatoli R.P. Cardon IV-V.1897” (MNHN); 5 exs., “Maissour Shinoga Mai 1897” (MNHN); 3 exs., “Val de Kangra [Himachal Pradesh] Dharamsala vers 1300 m, J. Berlioz, 1937” (NMB, MNHN); 1 ex., “Calcutta [Kolgata] Ex. Museo E. Steinheil” (MNHN); 1 ex., “Ex. E. Wehncke Acqu. 1884” (MNHN); 1 ex., “India bor. 980 Dejeani, Sharp Coll. 1905-313” (NHMUK); 1 ex., “Western Ghats, Bombay [Mumbay], 2250 feet, Matheran, Charlotte Lake, 31.III.1908 & pres. 1908 by G.B.Longstaff” (NHMUK); 1 ex., “S. Bombay 1902.294.” (NHMUK); 1 ex., “Matheran II.1919 P.H.”, “Brit. Mus. 1978-16” (NHMUK); 3 exs., “India N.W. frontier, E.Y. Watson 98-142” (NHMUK); 1 ex., “India orient”, “Fry Coll. 1905-100 (NHMUK); 1 ex., “India 980 Dejeani”, “Sharp Coll. 1905-313” (NHMUK); 3 exs., “Khandesh, T.R. Bell, H.E. Andrewes Bequest.”, “B.M. 1922-221” (NHMUK); 6 exs., “Khandesh, H.E. Andrewes Bequest. B.M. 1922-221” (NHMUK); 3 exs., “Belgaum, H.E. Andrewes Bequest. B.M. 1922-221” (NHMUK); 1 ex., “India” (NHMUK); 2 exs., “Nilghiri Hills, H.L. Andrewes” (NHMUK); 1 ex., “Achmednagar, Gebauer leg.” (NMW); 2 exs., “Shimoga, Et. Myore, V.1936” (MNHN); 6 exs., “South India, Salem District, IX.1943, P.S.Nathan leg.” (MNHN); 1 ex., “India” (MNHN); 2 exs., “India Sunderbunds” (MNHN); 6 exs., “India, Sundabunds” (MNHN); 1 ex., “India, Sundabunds”, “coll. Gärtner” (DEI); 1 ex., “S-India, Salem Distr. IX.1934 Nathan leg.” (NMB); 1 ex., “Orissa, Daitari, 31.XII.1966, collected on lamp, Gy Topal leg.” (NMB); 4 exs., “Orissa, Jaipur Keonjahr, District Daitari, 29.XI.1967, netted from water, Gy Topal leg.” (NMB); 1 ex., “Dehra Dun 1883 Dr.Warth leg.” (CGW); 1 ex., “Madhya Pradesh, Jablpur Dagmaga, 18.IV.1968, V.S.Durve leg.” (CGW); 1 ex., “Ostindien“, “Samml. A. Zimmermann” (ZSM); 1 ex., “Madras, “Samml. A. Zimmermann” (ZSM); 2 exs., “India or., Behar, “Samml. A. Zimmermann” (ZSM); 2 exs., “Khandesh., 27.II.1902, in water, T.R. Bell”, “Samml. A Zimmermann” (ZSM); 2 exs., “Coorg Hallery, Fletcher leg.”, “Samml. A. Zimmermann” (ZSM); 1 ex., “India, Bombay Biro.902” (TDMB); 2 exs., “India, Orissa, Jajpur-Keonjahr, District Daitari, leg. Gy Topál, No. 975, netted from water, 29.XI.1967” (TDMB); 1 ex., “India or.” (TDMB); 1 ex., “India, Madras” (TDMB); 2 exs., “Maharashtra, Igatpuri env. 120 km NE Mumbai, 600 m, 1.-12.VIII.2002, 19°42.17'N, 73°33.06'E, P. Šípek & M. Fikáček leg.” (NMPC); 1 ex., “Tamil Nadu, 15 km SE Kotagiri, Nilgiris, Kunchappanai, 900 m, 7.-22.V.2000, 11°22'N, 76°56'E, Rolčík leg.” (NMPC); 1 ex., “India S, Tamil Nadu, Nilgiris, 15 km SE of Kotagiri, Kunchappanai, 900 m, 11° 22’ N 76° 56’ E, 7.–22.V.2000, D. Hauck leg.” (CJS); 2 exs., “Bhimtal 20.V. 1300–1500 m”, “India U.P. 8.81 M. Brancucci” (NMB); 1 ex., “Southern Madhya Pradesh Dhobighat Nala [stream] (= Clematis Point Stream), Pachmarhi Wildlife Sanctuary, Satpura Mountain Range, ca 5 km SSE Panchmarhi, Hoshangabad District, 900 m a.s.l., 22°27'31"N/78°26'41"E, 27.II.2008, M. Jäch leg. (Loc. MP 7)” (NMW); 1 ex., “N27°08'22’’ E76°20'38’’, India bor. Occ., Rajasthan state, Alwar di., Naranimata env., 460 m, 20–30.7.2002, lgt.P. Šrámek” (NMPC). • **Iran**: 1 ex., “Sistan va Baluchestan Prov., Bampur, 6.-16.VI.1997, M.Kafka leg.” (NMPC); 1 ex., “Sistan va Baluchestan Prov., Pir Sohrab env., 100 m, pool in dried up Wadi, 11.-12.IV.2000, 25°44'N 60°50'E, J. Hájek & M.Mikát leg.” (NMPC). • **Myanmar**: 1 ex., “Mulmein [= Mawlamyaing], 1871, Fieber” (NMW). • **Nepal**: 1 ex., “Nepal” (NHMUK). • **Pakistan**: 1 ex., “Pakistan 20.05.1998 Kashmir Himalaya Mts. 20 km S Muzaffarabad 73°29'E,34°01'N, Nara village Ronkay” (CHF); 1 ex., “Kawai, Khagan Valley 1450–1800 m, 15.6.1977, Wittmer & Brancucci” (NMB); 1 ex., ”NW Pakistan, Swat Prov., Madyan, 1400 m, VII.1971, Holzschuh leg.” (CGW); 1 ex., ”West Pakistan, Rawalpindi surr., 18.X.1971, E. Heiss leg.” (CGW); 1 ex., “West Pakistan, Rawalpindi surr., Basal, 16.-18.I.1956, Kala Chitta Range, C. Lindemann leg.” (ZSM); 1 ex., “Pakistan, Northern Frontier Province, Tathabaya, 34°36'48 N, 73°27'01 E, 2300 m, at light, No 4–6, 7.-9.VII.1998, G. Csorba & L. Ronkay leg.” (TDMB).

#### Doubtfull record and probably mislabelled.

1 ex., “Philippines Luzon Ch. Semper” (MNHN).

#### Locality unknown.

4 exs., “leg. Stolicka 1865” (NMW); 2 exs., “Megerley” [= coll. Megerle] (NMW).

#### Remarks.

*Sandracottusvijayakumari* recently described by Anand et al. (2021) from the Western Ghats in Kerala, India is proposed as a junior synonym of *S.dejeanii*. The illustrated and described dorsal colouration of the head, the pronotum, and the elytra is within the range of variation of *S.dejeanii* which can have both separated and contiguous testaceous markings on the elytra. Furthermore, all 143 specimens of *S.dejeanii* examined by the authors, including the ones having largely separated testaceous patches on the elytra, have a fine microreticulation with numerous larger punctures on the head. No information was given in the original publication on any differences in genital structure of both taxa.

#### Redescription.

Body oval, shiny, testaceous to ferrugineus brown with black markings (Fig. 50). Ventral side completely dark brown to black, legs testaceous to ferrugineus brown.

Head testaceous with posterior half broadly black: black band protruding forwards to frons, shiny (Figs 4, 50). Surface sculpture consisting of dense microreticulation and of larger, much sparser punctures, particularly numerous on frons. Clypeal grooves, punctures alongside eyes and a transverse depression beside eyes distinctly impressed, punctures large and coalescent. Antennae testaceous; antennomeres slender, antennomere V 4× as long as broad.

Pronotum testaceous with a median black marking reaching from posterior to anterior margins (Figs 4, 50); long and broad posteriorly, narrower and shorter anteriorly, and strongly constricted in middle. Surface shagreened with a dense punctation; punctures medium-sized. Anterior and lateral puncture lines with dense and coalescent punctures, becoming sparse towards middle and lacking in the very middle of anterior margin. Posterior puncture line distinctly visible only at sides, superficial medially and transformed in long and very superficial wrinkles.

Elytra black to dark brown with testaceous markings in form of a chessboard, the testaceous markings alternating with the black ones. Epipleura testaceous to ferrugineus brown (Figs 4, 50). Surface very slightly and superficially shagreened and covered with a double punctation, a smaller and denser one as well as a larger and much sparser one. Puncture lines with groups of medium-sized punctures.

Ventral side dark brown. Legs particularly fore and mid legs testaceous, hind legs ferrugineus brown to dark brown. Prosternal process very short and broad, 1,3× only longer than broad, flattened and superficially sculptured; posterior border broadly rounded. Whole surface very superficially shagreened and finely punctured. Metatibia with only a few small punctures on outer half. Ventrites II–VI shagreened, densely covered with very small punctures and a few larger sparser ones. Posterior margins rounded, bordered with a short row of coalescent punctures in middle.

Measurements: TL = 12.0–13.0 mm, TL-h = 11.1–12.1 mm, TW = 7.3–7.6 mm.

♂. Protarsomeres I–III strongly enlarged with three larger suckers and numerous smaller ones. Mesotarsomeres I–III with two rows of small suckers. Median lobe of aedeagus, in ventral view, narrow and elongate, slightly tapered towards apex (Fig. 15a). Parameres also narrow, same length as median lobe, only slightly tapered at apical part (Fig. 15b).

♀. Similar to male. Tarsi not enlarged.

#### Differential diagnosis.

The combination of size and dorsal colour pattern (Figs 4, 50) separates *S.dejeanii* from all other species of the genus. Furthermore, the species can be separated from all other species of the genus by the shape of the median lobe and parameres (Fig. 15a, b).

#### Distribution.

India: Andhra Pradesh, Arunachal Pradesh, Rajasthan, Assam, Himachal Pradesh, Jharkhand, Karnataka, Madhya Pradesh, Maharashtra, Orissa, Punjab, and Tamil Nadu (Ghosh and Nilsson 2012; Deb and Subramanian 2023; Sheth et al. 2024); Nepal, Pakistan, Myanmar, and Iranian Baluchistan (Hájek 2006; Shaverdo et al. 2024) (Fig. 26). Specimens were collected from near sea level to 2300 m.

#### Habitat.

In India widespread in different ponds, rest pools of intermittent streams and artificial water tanks, often rich in aquatic vegetation. The single specimen in Pachmarhi Wildlife Sanctuary was obtained from a forest stream (ca 1–2 m wide) with rocky bottom (Fig. 35). In Kerala, Anand et al. (2021) collected the species in a partly shaded, shallow, ditch-like forest pool which was rich in decaying leaves and twigs; lentic habitat. In Iran it was collected in remnant pools in a wadi, in a semidesert area (Hájek 2006) (Fig. 34). Together with the Indian *S.festivus* and the two subspecies of the Australian *S.bakewelli*, this species is not restricted to forested areas as are many other species of the genus, especially in Southeast Asia. *Sandracottusdejeanii* is capable of flight and was attracted to light. The larvae have been described by Vazirani (1971).

### ﻿ Sandracottus femoralis

Taxon classificationAnimaliaColeopteraDytiscidae

Heller, 1934

6B89ECAA-735B-50BA-9E6E-CAB068266314

**Figure 27.**
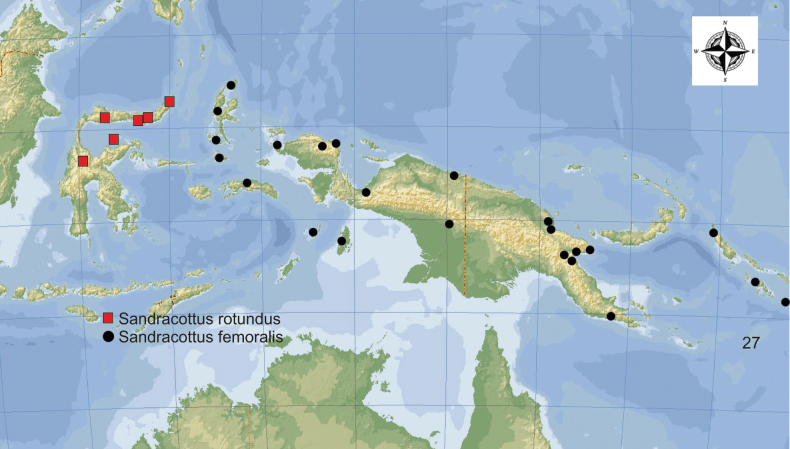
Distribution of *Sandracottusfemoralis* [black dots] in New Guinea and the Moluccas, and *S.rotundus* [red squares] on Sulawesi.

Figs 6 , 17 , 27 , 36 , 52

Dytiscus flavocinctus Guérin-Méneville, 1830: 61 (type locality Manokwari, West Papua, Indonesia); Boisduval, 1835: 49 (preoccupied by Hummel 1823 and objective synonym of Sandracottusguerini); Boisduval, 1835: 49 (descr.).
Sandracottus flavocinctus (Guérin-Méneville, 1830): Régimbart 1899: 339 (descr.); Zimmermann 1920: 234 (cat.).
Sandracottus femoralis Heller, 1934b: 3 (type locality: Buka, Solomon Islands); Alarie et al. 2023: 305 (larval descr.), idem: 320 (habitat); Hájek and Nilsson 2024: 91 (cat.).
Sandracottus guerini J. Balfour-Browne, 1939: 114, replacement name for Sandracottusflavocinctus; Hájek and Nilsson 2024: 91 (cat.) (syn. nov.).

#### Comments on classification.

*Sandracottusfemoralis* was described from the Solomon Islands. We have studied Heller’s type specimen of *S.femoralis*, Guérin-Meneville’s type of *D.flavocinctus*, as well as numerous specimens of *S.guerini* over its complete range. There is no doubt that both taxa belong to the same species. *Sandracottusguerini* Balfour-Browne, 1939 is a replacement name for *S.flavocinctus* Guérin-Méneville but is more recent than *Sandracottusfemoralis*, so the valid name for this species must be *S.femoralis*.

#### Type material

**of *Sandracottusfemoralis*. *Holotype***: Female, “Buka Salomonen Juli 1930”, “Coll. H. Hediger”, “1933 8” [blue label], “S.femoralis Typus” [red label], “Staatl. Museum für Tierkunde, Dresden”, “*Sandracottusfemoralis* Heller, 1934 Hendrich & Brancucci det. 2006”. Examined.

***Dytiscusflavocinctus*: *Lectotype*** (herewith designated): Female, “Mus. Paris Nouv. Guninée Dumont d´Urv. 1841”, “Dory [Manokwari] N. Guinee Duvelle Durville” [Dumont d´Urville], [round handwritten label], “Hidaticus [sic!] flavocinctus” [handwritten label], “Lectotypus Dytiscusflavocinctus Guérin-Men. des. M. Brancucci & L. Hendrich 2010” [red printed label], “Sandracottusguerini B.-Br. Det. M. Brancucci L. Hendrich 10” [white printed label] (MNHN). Examined.

#### Additional material.

**(118 specimens)**: • **Indonesia**: 1 ex., “Indonesia: Halmahera Isl., Khao Dist., Camp 34 34 km inland from Pan-Tunggal Lumber Co. base camp at Tg. Loleo”, “1-14 Feb. 1981 AC Messer & PM Taylor” (USNM); 1 ex., “Indonesia: Halmahera Isl., Jailolo Dist., Kampung Psir Putih 0°53'N 127°41'E”, “1-14 Aug. 1981 PM Taylor” (USNM); 13 exs., “Indonesia: Halmahera Isl., Jailolo Dist., Kampung Psir Putih 0°53'N 127°41'E”, “15–31 Jan. 1981 AC Messer & PM Taylor” (USNM); 18 exs., “Indonesia: Halmahera Isl., Jailolo Dist., Kampung Psir Putih 0°53'N 127°41'E”, “1-14 May 1981 AC Messer & PM Taylor” (USNM); 2 exs., “Indonesia: Halmahera Isl., Jailolo Dist., Kampung Psir Putih 0°53'N 127°41'E”, “14. Aug. 1981 PM Taylor” (USNM); 1 ex., “Batanta Island [Pulau Batanta], Arefi, 2004, A. Skale leg.” (CAS); 1 ex., “Bernstein Morotai”, “flavocinctus Aubé” (MNHN); 1 ex., “Aru Insel”, “Samml. A. Zimmermann” (ZSM); 1 ex., “Rosenberg, Ins. Aru” (RMNH); 1 ex., “Key Insel”, “Samml. A.Zimmermann” (ZSM); 2 exs., “Bernstein, Morotai” (RMNH); 1 ex., “N. Moluccas, Bacan Island, Mt. Sibela, 400 m, 14 km SE Labuha, primary forest, 2–13.II.1996, 0°38´ S Lat. 127°32´ E, leg. V. Siniaev & E. Afonin leg.” (CLH); 2 exs., “SE Moluccas Aru ISLS, WOKAM I. 17 km NE Wakua vill., 1–7-II.2022, St. Jakl leg.” (CLH, NMPC); 8 exs., “N Moluccas: Bacan, Wayaua, alt. m. 0050, 05.-16.vii.1985, J. Huijbregts” (RMNH); 1 ex., “Indonesia, Maluku, Obi Isl., South coast, 22 km N of Tapaya vill., Seribu Mts., 1200–1500 m, S. Jákl leg., 20.xi-10.xii.2008” (NMPC); 3 exs., “Indonesia, Papua Dekai, upper Brazza, 273 m, 2./3.vi.2015, -4.74108472466468 139.654211075976, Sumoked (Pap044)” (MZB, ZSM); 2 exs., “Indonesia Papua Barat, Kebar, shaded deep sandy irrigation roadside ditches, 584 m, 6.xi.2013, -0.80775253 133.05923529, UNIPA (BH031)” (ZSM); 4 exs., “Irian Jaya, Nabire, Nabire-Ilaga track, km 62, 250 m, 24.VII.1991, M.Balke & L.Hendrich leg. (IR 22)” (CLH, NMW); 1 ex., “Tami River Hollandia 1930 R.Voorhoeve leg.” (CLH); 1 ex., “Seram, E Wahai, 12.II.1989, M.Jäch” (NMW); 1 ex., “New Guinea, Wareo, 1933” (ZSM); 6 exs., “Dutch New Guinea: Humboldt Bay Dist. Pukusam Dist. West of Tami River vi.1937”, “B.M. Nat. Hist. London” (CLH, NHMUK). • **Papua New Guinea**: 1 ex., “Nouvelle Guinée” (MNHN); 1 ex., “New Guinea, NE Zenag-Lae, 200 m, 16.I.1979” (NMB); 10 exs., “N. Guinea mer. Rigo Lugio 1889 L. Loria” (MNHN, RMNH); 5 exs., “N. Guinea mer. Rigo VII.1889 L. Loria” (MNHN, NMW); 1 ex., “Morobe Prov. Wau, 1200 m 1.-15.V.1962 light trap J.Sedlacek leg.” (NMW); 1 ex., “Morobe Prov. Bulolo 700 m 9.XI.1962 J.&M.Sedlacek leg.” (NMW); 1 ex., “Morobe Kilolo Creek 7 km W Wau 1070 m 15.-21.I.1969 J.Sedlacek leg.”(NMW); 1 ex., “Morobe Prov. Garaina 700 m 21.III.1998 A.Riedel leg.” (NMW); 1 ex., “Morobe Prov. Wau 15.XII.1968 H.Ohlmus leg.” (ANIC); 6 exs., “Madang Prov. Batabag village 50 m XI.-XII.2000 5°08'S 145°46'E L.Cizek leg.” (NMB); 1 ex., “Madang Alexishafen, sago swamp, 22.V.1991, D.J.Larson leg.” (ANIC); 6 exs., “Madang, Nagada River near Nobanob, 12.VI.1991, D.J.Larson leg.” (ANIC, QM); 1 ex., “New Guinea Biro 96 Friedrich-Wilhelmshafen” (TDMB). • **Solomon Islands**: 1 ex., “Solomon Islands: New Georgia, 2 mls. W. Of Lamberte 1.IX.65. Roy. Soc. Exped. B.M. 1966-1”, “bomb crater pool, coast road” (NHMUK); 3 exs. (+ numerous larvae), “SOLOMON ISLANDS, GUADALCANAL, ca. 3.5 km SE of BARANA vill., (drying up stream in shaded gorge), 09°29.8'S, 159°59.5'E; 190 m, 24.xi.-14.xii.2013, Jiří Hájek leg.” (NMPC); 3 exs., “South Pacific, Solomon Is. GUADALCANAL I., 500–650 m Koso vill. Env., ca. 15–18 km SSE of Honiara, 1–18.XII.2016 St. Jakl leg.” (CLH).

#### Redescription.

Body broadly oval, submatt /slightly shiny, black with ferrugineus brown markings. Ventral side completely dark brown to black, legs testaceous, hind legs somewhat darker.

Head testaceous with posterior part broadly black leaving free two small testaceous spots on vertex (Figs 6, 52). Surface submatt, shagreened, consisting of dense and minute punctures not very uniform in size and of larger and much sparser ones, more numerous on frons. Clypeal grooves and punctures alongside eyes marked, punctures medium-sized but coalescent. Antennae testaceous; antennomeres slender, antennomere V 4× as long as broad.

Pronotum black with lateral margins ferrugineus brown, and narrow, testaceous, horizontal band, interrupted medially (Figs 6, 52). Surface submatt, distinctly shagreened, with very dense punctation; punctures medium-sized and distant of only 1–2× their own diameter. Anterior puncture line interrupted in middle; punctures relatively small but coalescent, forming wrinkles and becoming sparse medially and lacking in mid-length of anterior margin. Posterior puncture line with coarse and coalescent punctures in middle of each side, forming distinct wrinkles, distinctly smaller and spaced on disc.

Elytra black with small testaceous markings consisting of basal band sometimes reduced to different small spots at base, one postmedian spot, one preapical short and reduced band, and an apical spot (Figs 6, 52), submatt. Epipleura ferrugineus brown. Surface distinctly shagreened and covered with small and dense punctures as well as with larger and much sparser ones. Puncture lines with groups of medium-sized punctures mostly grouped in five or six punctures, groups closer together on discal line. Sutural puncture line marked only by few punctures on apical part.

Ventral side dark brown. Legs, particularly fore and mid legs testaceous, hind legs ferrugineus brown to dark brown. Prosternal process short and broad, 1.3× longer than broad, flattened, finely but distinctly sculptured; posterior margin broadly rounded. Metatibia with sparse medium-sized punctures on whole surface. Ventrites II–VI very superficially shagreened, slightly and longitudinally wrinkled on lateral parts, densely covered with very small punctures and larger sparser ones. Posterior margins rounded, deeply bordered with some large and coalescent punctures in middle of each side along margin.

Measurements: TL = 11.9–12.6 mm, TL-h = 11.0–12.8 mm, TW = 7.0–7.5 mm.

♂. Protarsomeres I–III strongly enlarged with three larger suckers and numerous smaller ones. Mesotarsomeres I–III with two rows of small suckers. Median lobe of aedeagus, in ventral view, broad, parallel-sided up to apex, here slightly broadened and broadly rounded (Fig. 17a). Parameres broad and pointed at apex (Fig. 17b).

♀. Similar to male, tarsi not enlarged. Microsculpture on ventrite VI as in male.

#### Differential diagnosis.

The combination of size and dorsal colour pattern (Figs 6, 52) separates *S.femoralis* from all other species of the genus. The very similarly coloured *S.maculatus* is always larger (TL = 14.0–16.1 mm) and more broadly oval than *S.femoralis* (TL = 11.9–12.6 mm). Furthermore, *S.femoralis* can be separated from all other species of the genus by the shape of the median lobe and parameres (Fig. 17a, b). All instar larvae were described by Alarie et al. (2023).

#### Distribution.

Indonesia: Moluccas (Bacan, Batanta, Halmahera, Obi, Seram, Morotai), Key and Aru Islands, and West Papua; Papua New Guinea up to the Solomon Islands (Fig. 27).

#### Habitat.

In West Papua specimens were collected in small but mainly exposed primary rainforest pools and puddles, always enriched with decaying leaves (Fig. 36). On Bacan Island also collected at light. On the Solomon Islands found in an old water-filled bomb crater near a coastal road (New Georgia), and a pool formed by the drying up of a stream on a clay bed, covered with decaying leaves and twigs (Guadalcanal; Alarie et al. 2023: 320). *Sandracottusfemoralis* is distributed from lowland forest up to hilly mountain rain forests at ~ 1200 m altitude.

**Figure 28.**
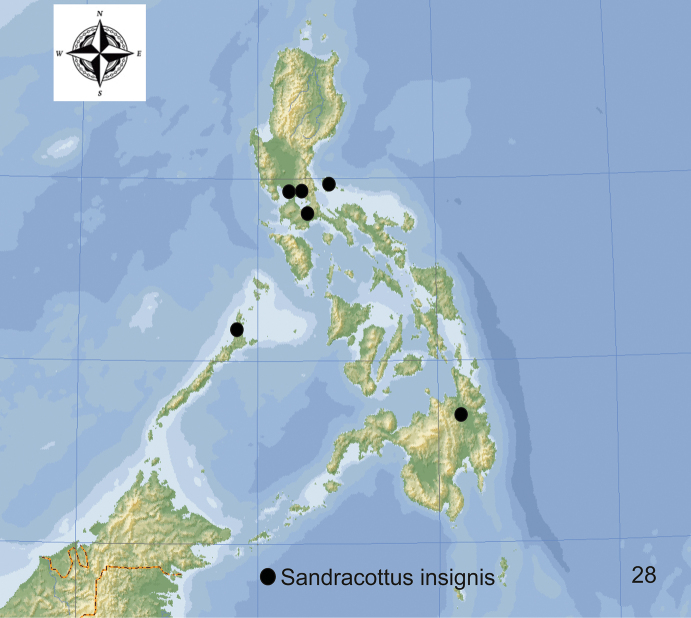
Distribution of *Sandracottusinsignis* in the Philippines.

### ﻿ Sandracottus festivus

Taxon classificationAnimaliaColeopteraDytiscidae

(Illiger, 1801)

525800FF-A282-543E-82EA-0B28DF53EBBA

**Figure 29.**
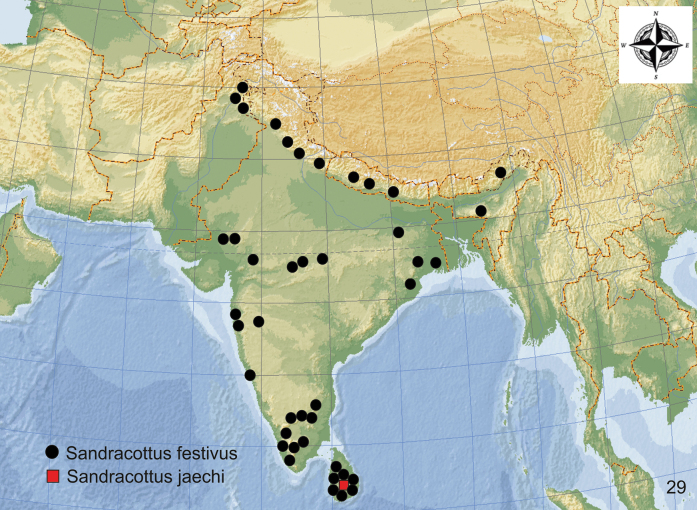
Distribution of *Sandracottusfestivus* [black dots] and *S.jaechi* [red square] in the Indian subcontinent.

Figs 5 , 16 , 29 , 35 , 51

Dytiscus festivus Illiger, 1802: 166. (type locality: “Ostindien [“East India” = India and the Malayan Archipelago]”.
Sandracottus festivus (Illiger, 1801): Sharp 1882: 686 (comb. nov.); Régimbart 1899: 334 (descr.); Zimmermann 1920: 234 (cat.); Vazirani 1969: 273 (descr., cat.); Ninje Gowda and Vijayan 1992: 29–32 (ecol.); Ghosh and Nilsson 2012: 18 (cat.); Ghosh and Hegde 2015: 73 (faun.); Sonali et al. 2022: 339 (faun.); Hájek and Nilsson 2024: 91; Sheth et al. 2024: 8, 20 (check list, faun., key).
Sandracottus mixtus (non Blanchard, 1843): Shankar et al. 2023: 455 (faun., misidentified, photo shows S.festivus); Deb and Subramanian 2023: 10–15 (faun., misidentified, photo shows S.festivus).

#### Type material.

Not examined.

#### Additional material.

**(202 specimens)**: • **China**: 1 ex., “Chine”, “Samml. A. Zimmermann” (ZSM).• **India**: 1 ex., “Panjab and U Provinces VI-X India”, “RL Woglum coll.” (USNM); 2 exs., “Mahableshnar W. Ghato [Ghatol, Rajastan], 5500ft India”, “Coll´n J.D. Sherman Jr. 1926” (USNM); 2 exs., “INDIA – Tamil Nadu Road Salem – Yercaud Pond at 8–9 km from Yercaud – 1000 m 4.I.1995 Mazzoldi P. leg.” (CPM); 9 exs., “INDIA - Kerala m 700 – Road Thekkadi – Kottayam at 13 km from Thekkadi – Muddy pool 2.I.1995 Mazzoldi P. leg.” (CPM); 6 exs., “Aug. 43 Dehra Dun” [Prov. Uttarakand, October 1943, Dehradun, Central Internment Camp for British India near Premnagar, 30°20 N 78°3 E] (ZSM); 6 exs., “Montagnes du Wynaad”, “Museum Paris ex Coll. R. Oberthur” (MNHN); 3 exs., “Solan près Simla Lakhat 1896” (NMB); 3 exs., “Pulney Hills R.P. Castets 1898” (MNHN); 4 exs., “Maissour, Shinoga, Mai 1897” (MNHN); 2 exs., “Himalaya Simla”, “Coll. Franklin Müller” (DEI); 1 ex., “Simla, IV-VII.96”, “Ex. Musaeo W. Rothschild 1899” (MNHN); 3 exs., “Bangalore, Silvepoore, G. Tabourel 1899” (MNHN); 3 exs., “Val de Kangra [Himachal Pradesh], Dharamsala, vers 1300 m, J. Berlioz, 1937” (MNHN); 1 ex., “Inde, Mont Abu, Tajputana, 1938, J. Berlioz Ex Musaeo Thorey” (MNHN); 1 ex., “Calcutta, Ex. Museo E. Steinheil” (MNHN); 4 exs., “Punjab, Simla, E.C. Ansorge B.M. 1922-455” (NHMUK); 1 ex., “Bangalore, Mysore 95-28” (NHMUK); 5 exs., “N.W. India 8438” (NHMUK); 1 ex., “Simla, Coll. Plason” (NMW); 1 ex., “South India, Salem District, IX.1943, P.S.Nathan leg.” (MNHN); 1 ex., “South India, Coimbatore, 24.VIII.1937, P.S.Nathan leg.” (MNHN); 1 ex., “India (M.P.) Jabalpur district Lamhetghat village 28.III.1962, R.G. Sharna leg.”, “Zoological Survey of India Central R.S. Lot No. 29/63 F.C. No. 362” (NMB); 1 ex., “Parasnath Hills& Panchi Survey”, “IV.1918 Sinha & Nath” (NMB); 1 ex., “India” (MNHN); 1 ex., “Coimbatore Inde merid. IX.1933” (MNHN); 3 exs., “Wagrar-Karur env. de Bellary, 1883, Chaper & de Morgan” (MNHN); 6 exs., “Mahé Malabar” (MNHN); 3 exs., “Côte de Malabar, T. Deschamps 1900” (NMB); 1 ex., “Orissa, Jaipur-Keonjahn Distr. Daitari, 29.XI.1967, collected from water, Gy Topal” (NMB); 1 ex., “Madhya Pradesh, Jablpur Dagmaga, 31.V.1965, H.P.Agywal leg.” (CGW); 1 ex., “India or.” (CGW); 1 ex., “Karnataka, Western Ghats Mts., 30 km SEE Bhatkal, Kollur env., 26.-28.V.2006, V.Ryjacek” (NMPC); 1 ex., “Tamil Nadu, Nilgiri Hills, Kunchappanai, 15 km SE Kotagiri, 900 m, 11°2'N 76°56'E, 13.-20.V.1994, Z.Kejval leg.” (NMPC); 2 exs., “Rajasthan, Mt. Abu env., 100 km W Udaipur, 1150 m, 24°35.35'N 72°42.72'E, 24.-27.VIII.2002, P.Sipek & M. Fikacek leg.” (NMPC); 1 ex., “Ostindien Coll. C. Felsche Geschenk 1907” (SMTD); 1 ex., “India or. post.”, “Coll. Maerkel” (SMTD); 1 ex., “Madras, Coimbatore, 1400 feet, X.1964, P.S. Nathan leg.” (ZSM); 2 exs., “India or.” (ZSM); 1 ex., “Indien, Tanquelar” (ZSM); 2 exs., “Sammlung Cl. Müller” (ZSM); 2 exs., “S-India, Kerala, Theimala nr. Shenkottah (70 km N Trivandrum) 150 m, 8°57'N 77°01'E, 5.IV.1997, Schintlmeister & Siniaev leg.” (CLH); 1 ex., “S India Rippon coll.” (CLH); 2 exs., “Khasi Hills” (NHMUK); 1 ex., “Ranikhet, Kumaon, H.G.C., H.G. Champion Coll. B.M. 1953-156” (NHMUK); 10 exs., “W.Almora Division, Kumaon, U.P. H.G.C., H.G. Champion Coll. B.M. 1953-156” (NHMUK); 2 exs., “W.Almora Division, Kumaon, U.P., I.1920”, “H.G. Champion Coll. B.M. 1953-156” (NHMUK); 2 exs., “W.Almora Division, Kumaon, U.P., IV.1917”, “H.G. Champion Coll. B.M. 1953-156” (NHMUK); 8 exs., “India, Orissa, Jajpur-Keonjahr, District Daitari, No. 975, netted from water, 29.XI.1967, leg. Gy Topál” (TDMB); 1 ex., “Rhimtal, 25.IX.1979, Smetacek” (NMB); 1 ex., “Rhimtal, 2.VIII.1973, Smetacek” (NMB); 1 ex., “S-India, Kerala, 15 km SW Munnar, Kallar Valley, 9.V.1997, 1250 m, 10°02'N 76°58'E, Dembicky & Pacholatko leg.” (NMB); 1 ex., “Tamil Nadu, Nilgiris, 15 km SE of Kotagiri, Kunchappanai, 900 m, 11° 22’ N 76° 56’ E, 7.–22.V.2000, leg. D. Hauck” (CJS); 1 ex., “Tamil Nadu, Nilgiri Hills, 11 km SE Kotagiri, 1100 m, E Kunchappanai, 11°24'N 76° 56'E, 3.-15.V.2002, P.Pacholatko leg.” (NMB); 1 ex., “Southern Madhya Pradesh Hoshangabad District Pagara – Panchmarhi road, ca. 5 km NNE Panchmarhi, Panar Pani [stream], 850 m a.s.l. 22°30'25"N/78°26'43"E, 26.+27.II.2008 M. Jäch leg. (Loc. MP 6)” (NMW); 2 exs., “Nilghiri”, “Coll. Kraatz Régimbart det.”, “Sandracottusfestivus Illiger”, “Zimmermann det.” (DEI); 1 ex., “Calcutta” (NHMUK). • **Nepal**: 1 ex., “Nepal 02.08.1981 Khare 1600 Beron” (CHF); 1 ex., “Dhading distr., Thorpu bis Kordunje, 1300–1400 m, 24.VII.1983, Martens & Schawaller leg.” (NMB); 2 exs., “W Nepal, Kali Gandaki Khola Bhakta B. Tatopani 1100–1400 m, 12.-14.V.1984” (NMB); 1 ex., “Himalaya” (RMNH); 3 exs., “S Dhaulagiri, W Beni Darbang, 1150 m, 1.XII.2000, G.Riepl leg.” (NMW). • **Bhutan**: 2 exs., “Bhakta B., Beguna, 2730 m, 24.VIII.1976” (NMB). • **Sri Lanka**: 3 exs., “Belihul-Oya Ceylon 2 de trim. 89 I.Z. Kannegieter”, “Muséum Paris, Coll. R. Oberthür” (MNHN, CLH); 1 ex., “Ceylon”, “W Robinson bequest 1929” (USNM); 2 exs., “Ceylon N.P. Madugoda 15.IX.1953, F. Keiser” (NMB); 1 ex., “Museum Paris Inde Bellary du Ceylan de Morgan 1896” (MNHN); 1 ex., “Kandy, Ceylon H. Rolle, Berlin, SW”, “coll. Gärtner” (DEI); 1 ex., “Süd Ceylon Mai 1889 H. Fruhstorfer”, “Coll. Kraatz Régimbart det.”, “Hydat. festivus”, “Zimmermann det.” (DEI); 15 exs., “Nalanda Ceylon W.Horn 1899”, “Zimmermann det.” (DEI); 3 exs., “Paradna W.Horn 1899”, “Sandracottusfestivus Illig, Zimmermann det.” (DEI); 11 exs., “Ceylon Kannegieter 1889” (MNHN); 3 exs., “Ceylon Nalanda 2e trim. 89 I.Z. Kannegieter” (MNHN); 1 ex., “Ceylon, Belihul-Oya, 2e trim. 89, I.Z. Kannegieter” (MNHN); 1 ex., “Ceylan, Deschamps, 1889” (MNHN); 4 exs., “Kandy, IX-XII.1897, E.E. Green, 1917-54” (NHMUK); 2 exs., “Kelani Valley nr. Colombo W. Braine 1910-283” (NHMUK); 4 exs., “Ceylon G. Lewis 1910-320” (NHMUK); 1 ex., “Ceylon, 1891 Heuser leg.” (NMW); 1 ex., “Kandy X.1907 H.E. Andrewes Bequest B.M. 1922-221” (NHMUK); 1 ex., “Kandy, 20.II.1902 Dr.Uzel leg.” (NMW); 6 exs., “Haputale env., Beragala, 9.XII.1980, M.Jäch leg.” (CGW, NMW); 2 exs., “Dambulla env. 300 m 19.IV.-9.V.1991 J.Kolibac leg.” (NMB); 1 ex., “Habarana 10.XI.1982 G.Duranton leg.” (NMB); 1 ex., “Sigiriya 10.XI.1982 G.Duranton leg.” (NMB); 1 ex., “Anuradkapura 3.XI.1982 G.Duranton” (NMB); 1 ex., “Paradina 1899 W. Horn” (ZSM); 1 ex. “Matale Ceylon 1899 leg. W. Horn” (ZSM); 1 ex., “Ceylon Madarász Madatugama 21.II.1896”, “Sandracottusfestivus Illiger Guignot det. 1956” (TDMB); 9 exs., “Sri Lanka Moneragala Kumaradola group X.1997 M.M. Bahir & S.V. Nanayakkara leg.” (CLH); 1 ex., “Sri Lanka, Ratnapura Sincharaja rain forest, 17.-19.II.1997, Udovichenko leg.” (CLH). • **Pakistan**: 1 ex., “Hazara, Balakot 900–1100 m, 3.-7.VI.1983, leg. Eckweiler” (NMB); 1 ex., “Islamabad, 1 km S Hotel Adventure Inn, 500 m, 3.VII.1998, leg. Gy. Fábian & B. Herczig” (TDMB); 1 ex., “Pakistan, Kashmir, Himalaya Mts., 20 km S Muzaffarabad, Nara village, 73°29'E, 34°01'N, 750 m, 12.IX.1997, leg. Gy. Fábian & G. Ronkay” (TDMB).

#### Country unknown.

1 ex., “Novara Reise, 1857-1859” (NMW).

#### Redescription.

Body broad oval, shiny, testaceous with black markings. Ventral side completely dark brown to black, legs testaceous, hind legs somewhat darker.

Head testaceous with posterior part and broadly so on posterior half alongside as well as two elongate spots on clypeus black, shiny (Figs 5, 51). Surface almost smooth consisting of dense and very numerous punctures of different size and of larger, much sparser ones, particularly numerous on frons. Clypeal grooves, punctures alongside eyes and transverse depression beside eyes distinctly impressed, punctures large and coalescent. Antennae testaceous; antennomeres slender, antennomere V 4.5× as long as broad.

Pronotum testaceous with median black marking reaching from posterior to anterior margins; long and broad on posterior, distinctly shorter and narrower on anterior margin and strongly constricted on middle (Figs 5, 51). Surface very superficially shagreened, almost not discernible, with dense punctation; punctures medium-sized mixed with smaller ones. Anterior and lateral puncture lines dense and coalescent, punctures becoming sparse towards middle and lacking in very middle of anterior margin. Posterior puncture line with coarse and coalescent punctures on middle of each side, distinctly smaller and spaced on disc.

Elytra testaceous to ferrugineus brown with black markings consisting of three transverse bands (Figs 5, 51); particularly characterised by presence of a longitudinal testaceous subsutural spot on anterior third. Epipleura testaceous to ferrugineus brown. Surface very slightly and superficially shagreened and covered with double punctation, a smaller and denser one as well as a larger one that is more sparsely distributed. Puncture lines with groups of medium-sized punctures mostly grouped by 5–6 punctures; groups closer together on discal row.

Ventral side dark brown. Legs, particularly fore and mid legs testaceous, hind legs ferrugineus brown to dark brown. Prosternal process short and broad, 1.5× longer than broad, flattened finely but distinctly sculptured; posterior margin broadly rounded. Whole surface very superficially shagreened and finely punctured. Metatibia with sparse medium-sized punctures on outer half. Ventrites II–VI very superficially shagreened, slightly and longitudinally wrinkled on lateral parts, whole surface densely covered with very small punctures and larger sparser ones. Posterior margins rounded, bordered with some large and coalescent punctures on middle of each side.

Measurements: TL = 14.7–15.5 mm, TL-h = 13.5–14.4 mm, TW = 8.0–8.8 mm.

♂. Protarsomeres I–III strongly enlarged with three larger suckers and numerous smaller one. Mesotarsomeres I–III with two rows of small suckers. Median lobe of aedeagus, in ventral view, broad, parallel-sided up to apex here slightly broadened and broadly rounded (Fig. 16a). Parameres broad and pointed at apex (Fig. 16b).

♀. Similar to male, tarsi not enlarged. Microsculpture on ventrite VI as in male.

#### Distribution.

India: Andhra Pradesh, Arunachal Pradesh, Assam, Himachal Pradesh, Jharkhand, Madhya Pradesh, Maharashtra, Orissa, Punjab, and Tamil Nadu (Ghosh and Nilsson 2012; Deb and Subramanian 2023; Sheth et al. 2024); Pakistan, Nepal, Bhutan, Sri Lanka (Fig. 29). Specimens were collected between 150 and 2700 m above sea level. Zeng (1989) in his doctoral thesis recorded *S.festivus* from numerous provinces in southern China (see also Nilsson 1995). However, revision of Zeng’s specimens revealed that he misidentified *S.festivus* and *S.mixtus* [= *hunteri*] (S. Zhao, in litt. 2020). The single historical specimen from China deposited in ZSM is probably mislabelled. For now, we consider *S.festivus* as not occurring in China.

#### Differential diagnosis.

The dorsal colour pattern of the Indian *S.festivus* is near to the Indonesian *S.chevrolati* but can be easily separated by its consistently larger size (TL = 14.7–15.5 mm, *S.chevrolati*: TL = 10.0–13.5 mm) (Figs 5, 51), its distributional range (Figs 25, 29), and shape of median lobe and parameres (Figs 14a, b, 16a, b).

#### Habitat.

In India, Pakistan, and Sri Lanka the species is collected from protected embayments of slow flowing forest streams, ponds, swamps, and artificial water tanks, often rich in aquatic vegetation. At Panar Pani [stream] in broad but slow flowing forest stream (ca 3–5 m wide), with its bottom consisting of gravel and rocks (Fig. 35). Together with the Indian *S.dejeanii* and the two subspecies of the Australian *S.bakewellii*, this species is not restricted to densely forested areas as many other species of the genus, especially in SE Asia. Ninje Gowda and Vijayan (1992) have published their research results on the predatory behaviour of the species on mosquito larvae in sewage tanks. The species is capable to flight and was attracted to light.

### ﻿ Sandracottus hunteri

Taxon classificationAnimaliaColeopteraDytiscidae

(Crotch, 1872) stat. rev.

1C09BEED-A7AC-5703-B71B-B97BDCCBA261

Figs 7 , 18 , 30 , 37 , 38 , 40 , 41 , 53

Dytiscus fasciatus Fabricius, 1775: 825 (type locality India).
Hydaticus hunteri Crotch, 1872: 205 (type locality China) (replacement name for Dytiscusfasciatus Fabricius).
Sandracottus fasciatus (Fabricius, 1775): Régimbart 1899: 333 (descr.); Zimmermann 1920: 234 (cat.); Hendrich and Balke 1995: 47 (ecol., cat.).
Sandracottus mixtus (non Blanchard, 1843): Hendrich 1995: 48 (ecol.); Park et al. 2009: 47 (check list); Ghosh and Nilsson 2012: 19 (cat.); Lee et al. 2014: 36. (faun.); Suprayitno et al. 2022: 135 (check list, faun.); Atthakor et al. 2018: 93 (faun.); Ghosh and Gupta 2012: 110 (faun.); Alarie et al. 2023: 307 (larval descrip.); Hájek and Nilsson 2024: 91 (cat.).
Sandracottus fasciatus var. crucialis Régimbart, 1899: 333 (type locality Vietnam, Myanmar); Vazirani 1977: 85 (syn.).
Sandracottus manipurensis Vazirani, 1969: 277 (type locality Manipur, Assam, India); Mukhopadhyay and Ghosh 2004: 368 (cat.); Ghosh and Nilsson 2012: 19 (cat.); Hájek and Nilsson 2024: 91 (cat.) (syn. nov.).
Sandracottus hunteri (Crotch, 1872): Sharp 1884: 447 (faun., check list); Jäch et al. 2012 (faun.); Suprayitno et al. 2017: 35 (faun., ecology).

#### Type material of *Dytiscusfasciatus*.

***Holotype***, 1 male: “Dyt. Fasciatus Fabr. Pag. [page] 293 No 7” [handwritten label], “Compared with modern examples in Brit. Mus. R.A.G. March 1926” [printed label with red letters], “Hunter Coll. ZIHU 2199” [printed label, museum no.], “Type” [printed label with red frame], “139881” [catalogue number GLAHM 139881], “*Dytiscusfasciatus* Fab. *Sandracottusfasciatus* Fab.” [printed label with red frame] (HMUG). Examined.

#### Type material of *Sandracottusmanipurensis*.

***Holotype*** not examined (ZSI). ***Paratype***, 1 male: “India Assam, Manipur 5000 ft. Phaiphenigaum 30.VIII.60 F. Schmid” [handwritten label], “Z.S.I. Lot No 61 1960” [printed label], “PARATYPE” [printed label], “*Sandracottusmanipurensis* n.sp. T.G. Vazirani, det.” [handwritten and printed label], “5147 H4A”, “*Sandracottushunteri* (Crotch) det.M.Brancucci & L.Hendrich 10” (ZSI). Examined.

#### Additional material.

**(473 specimens)**: • **Nepal**: 7 exs., “E-Nepal, Dhankuta-Hills, Thamur Valley, 1150–2000 m, 23.-25V.1983, M.Brancucci leg.” (NMB). • **India**: 2 exs., “NE India, Assam Bhalukpong, 150 m, 27°02'N 92°36'E, 26.V.- 3.VI.2005, Dembicky leg.” (NMB); 2 exs., “NE India, Arunachal Prov., 8 km S Jamiri Sessa vicinity, 27°07'N 92°34'E, 26.V.-4.VI.2005, 350 m, P. Pacholatko leg.” (NMB); 1 ex., “Darjeeling Distr., Alghera, 2900 m, 25.IV.1982, Ch.J.Rai leg.” (NMB); 3 exs., “Darjeeling Distr., Kalimpong env., III.-IV.1989, Ch. J. Rai leg.” (NMB). • **China**: 1 ex., “China Kouy-Tcheou [= Guizhou] Kouy-Tcheou, Reg. de Pin-Fa, 1909 Cavalerie” (CHF); 1 ex., “China Yunnan-See, 1898 Excoiffier” (CHF); 1 ex., “Chengtu 1933” [Chengdu], “Szechwan China DC Graham XI-27-1700ft” (USNM); 1 ex., “Mu Dong Szechwan China DC Graham 6-15-33 Alt 1100ft” (USNM); 7 exs., “Spirit Valley”, “Nanking Kiangsu Province”, “China H.F. Loomis Oct. 25, 1919” (USNM); 2 exs., “China 1942 Yun Hsien March WL Jellison” (USNM); 17 exs., “Suifu Sz. China”, “June 1928”, “DCGraham Collector” (USNM); 1 ex., “Suifu Szchuen China 1923”, “DCGraham Collector” (USNM); 1 ex., “Suifu 1000-2000ft Szchuen China 23´ DCGraham Collector” (USNM); 8 exs., “Suifu Szchuen”, “DCGraham Collector” (USNM); 1 ex., “China, Hayan Isl. Wuzhi Shan Mt. [Hainan, Wuzhi Shan Nature Reserve], 1500 m 18°53'N 109°43'E 20.02.-10.04.2001 local collector”, “coll. H. Hebauer” (CHH); 2 exs., “Chine A. David” (MNHN); 1 ex., “Su Tschuen Siao Lou 1897“ (MNHN); 1 ex., “Guanxi, 350 m a.s.l. 10 km S Yangshuo, muddy pools, fields, pasture 3.XI.1999 leg. J. Šťastný” (CJS); 5 exs., “Pingshiang [Pingxiang, Jiangxi] Süd-China Dr. Kreyenberg, Coll. Kraatz”, “Zimmermann det.” (DEI); 3 exs., “Kiukiang [Jiujiang] June 1887 AE Pratt”, “Coll. Kraatz Régimbart det.” (DEI); 2 exs., “Kiukiang July 1887 AE Pratt”, “Coll. Kraatz Régimbart det.” (DEI); 2 exs., “Kiangsi [Jiangxi] China”, “Coll. Kraatz Régimbart det.”, “Zimmermann det.” (DEI); 1 ex., “China, coll. Gärtner” (DEI); 2 exs., “China Prov. Hupeh [Hubei] Mts. Wu-schan” (DEI); 2 exs., “Su Tschuen, Siàn-Lou 1897” (MNHN); 1 ex., “China, Kouang-si, P. Barrière 1909 ex. Coll. Oberthür” (MNHN); 33 exs., “Su Tschuen Chasseurs Indigènes 1903” (MNHN); 20 exs., “Sian-Lou Chasseurs du P. Dejean 1904” (MNHN); 1 ex., “China Kouangsi Rég. de Nanning 1931” (MNHN); 1 ex., “Foochau [Fuzhou, Fujian] April 1886 Leech” (MNHN); 4 exs., “Kiukiang [=Jiujiang] June 1887 A.E Pratt” (MNHN); 4 exs., “Su-Tchuen, Chasseurs Indigènes 1907” (MNHN); 2 exs., “Su-Tchuen, Siàn-Lou 1897” (MNHN); 1 ex., “Bas-Yunnan A. Salvat 1904” (MNHN); 2 exs., “Chine, A. David“ (MNHN); 2 exs., “Kiang-Si A. David” (MNHN); 3 exs., “Kouy Tchéou Abbé Largeteau” (MNHN); 6 exs., “Kouy Tchéou Rég. De Pin-Fa Père Cavalerie 1908” (NMB, MNHN); 1 ex., “Chine Shanghai” (MNHN); 1 ex., “China bor. Ex Musaeo Thorey” (MNHN); 4 exs., “China“ (NHMUK); 1 ex., “Danes Island [Changzhou Island, Guangdong] 6756” (NHMUK); 4 exs., “Canton [Guangzhou, Guangdong], C.W. Howard, B.M. 1922-22” (NHMUK); 5 exs., “Da-Iaen-saen near Nong-po [village near Ningpo, Zhejang] Walker Coll. 93.-18.” (NHMUK); 3 exs., “Hong Kong, J.J.Walker”, “G.C.Champion Coll. B.M.1927-409” (NHMUK); 19 ex., “China, Da-Iaen-saen [village near Ningbo, Zhejang], J.J.W.”, “G.C.Champion Coll. B.M.1927-409” (NHMUK); 16 exs., “Western China Suiling W.A. Maw. 1909-38” (NHMUK); 1 ex., “Prov. Hunan, Huitong, 550 m, 9.-16.VII.1992, Ji Lanzhou leg.” (NMW); 1 ex., “Yunnansen”, “From R. Oberthür”, “Coll´n J.D. Sherman Jr. 1926” (USNM); 1 ex., “Yünnan, Xishuangbanna ca. 10 km NW Menglun, ca. 700–800 m, 7.XI.1999, leg. Jäch et al. (CWBS360)” (NMW); 1 ex., “SE-Guangxi, District Yulin, Liuwan Mts. SW Yulin, 350–400 m, 16.XI.1993, Schillhammer leg. (20)” (NMW); 1 ex., “Kiu Kiang [=Jiujiang] 1889”, “Coll. Kraatz” (NMW); 2 exs., “Nanking, Dr.Jettmar” (NMW); 1 ex., “China, Dr.Stoltz BH” (NMW); 1 ex., “Kiukiang [=Jiujiang] VII.1887 A.E.Pratt” (NMW); 1 ex., “Chekiang, Chusan, 17.VII.1931, O.Piel leg.” (MNHN); 2 exs., “China” (MNHN); 2 exs., “Prov. Hupeh” (MNHN); 1 ex., “Kiangsi, Staudinger & Bang Haas” (MNHN); 1 ex., “Szechuan, Chungking, E.Reitter” (MNHN); 1 ex., “Kuong Si” (MNHN); 2 exs., “China Yunnan” (MNHN); 1 ex., “S-China Canton S.W.Howard”, “BM 1922-22” (CGW); 3 exs., “Central China”, “Nonfried Coll.” (CGW); 5 exs., “W-Guizhou Prov., Leigongshan, Xijiang, 1200–1900 m, 29.V.-2.VI.1997, Bolm leg.” (NMB); 2 exs., “Hainan (217) 6 km W Dongxing, 50 m, 25.I.1996, Jäch leg.” (NMW); 37 exs., “Chine Tchékiang [Ningpo] Coll. David” (MNHN); 1 ex., “Ou Hou“, “Gebrüder W. Müller, Vermächt. 1909” (SMTD); 2 exs., “Kiangsi [Jiangxi]” (SMTD); 2 exs., “Kiautschau” (SMTD); 3 exs., “Shanghai“, “Coll. C. Felsche Geschenk 1907” (SMTD); 3 exs., “Kiukiang [Jiujang] June 1887 A.E. Pratt”, “Samml. A. Zimmermann” (ZSM); 3 exs., “China, “Samml. A. Zimmermann” (ZSM); 4 exs., “China, Kiautschou”, “Samml. A. Zimmermann” (ZSM); 2 exs., “China, Pingshiang”, “Samml. A. Zimmermann” (ZSM); 2 exs., “China, Kiaugsi”, “Samml. A. Zimmermann” (ZSM); 1 ex., “China Su-Tchuen chasseurs indigènes 1903” (CLH); 2 exs., “China, Central Sichuan Baoguo Emet Co, 27.VII.-3.VIII.1994, Benes leg.” (CLH); 4 exs., “Sechuan, Pingwu, 32°15'N 104°16'E, 3.- 9.VI.1997, E. Kucera leg.” (CLH); 1 ex., “China, Shanghai”, “Coll. E. Csiki”, “Sandracottus fasciatus v. hunteri Crotch” (TDMB). • **Myanmar**: 6 exs., “Taunggyi, Burma 2'27 Miss Northup” (USNM); 1 ex., “Myanmar Catcin Cauri Birranta Fea Ag. Nov. 1886” (MNHN); 1 ex., “Myanmar Sagaing Division, Alaungdaw Katthapa NP, Khaung Din stream, ca. 450 m, 11.V.2003, Boukal et al. leg. (119)” (NMW); 1 ex., “Myanmar Sagaing Division, Alaungdaw Katthapa NP, Khaung Din stream, ca. 360 m, 7.V.2003, Boukal et al. leg. (119)” (NMW); 4 exs., “Myanmar, Sagaing Division, Chatthin Wildlife Sanctuary, 23°31.481 N 95°38.804 E, ca. 260 m, 9.X.1998, Schillhammer leg. (9)”; 4 exs., “Myanmar Shan State, NE Mintaingbin Forest Camp [ca. 35 km N Aungban] above (150), pool, 22.VIII.2004, Shaverdo leg. (152)” (NMW); 6 exs., “Myanmar Shan State, NE Mintaingbin Forest Camp above (150), ca. 1200 m, puddles, 14.-20.VIII.2004, Shaverdo leg.” (NMW); 6 exs., “Myanmar Shan State, NE Mintaingbin Forest Camp above (150A), ca. 1200 m, pond, 14.-20.VIII.2004, Shaverdo leg.” (NMW); 1 ex., “Myanmar Shan State, Shwedaung Wildlife Sanctuary, 23°05.129 N 96°13.527 S, 360 m, 19.VII.2002, M.Hlaing & A.Moa leg.” (NMW); 9 exs., “Myanmar SW Shan-State, 70 km W Taungguyi, 10.-11.VI.1997, J. Rejsek leg.” (CJS, NMPC); 1 ex., “SW Shan state, Inle lake Nyaungshwe, 7.-16.VI.1997, J. Rejsek” (CJS); 1 ex., “Myanmar Shan State Kalaw env. 1356 m N 20.63200” E 96.56197” 8.-20.6.2015 Walter Grosser lgt.” (CLH); 10 exs., “Myanmar, Shan-State, near Kalaw, 20°36'48´´N 96°34'46´´E, 1400 m, 15.XI.2003, M. Hornburg leg.” (CLH). • **Cambodia**: 1 ex., “Cambodscha Schmidt” (MNHN); 5 exs., “Pnom Penh V.de Salvara” (MNHN); 1 ex., “Cambodia sept. or., Stung Treng 18.-22.IV.1998, 13°32'N 105°58'E, J. Mlíkovský leg.” (NMPC). • **Laos**: 1 ex., “NE Laos Huaphanne Prov. Mt. Phu Pane, 1220–1900 m Ban Saluei v.env., 18.V.-2.VI.2012 20°12'N 103°59'E, St. Jakl + Lao collector leg. Coll. Hendrich” (CLH);1 ex., “Khammouan Prov., Nakai env., 500–600 m, 22.V.-8.VI.2001, 17°43'N 105°09'E, E. Jendek & O. Šauša leg.” (CHH); 8 exs., “Laos Attapeu prov., Ban Vang Tat Noi env. 900 m, 15°03-04'N / 107°24'E, 10.-25.V.2011”, “NHMB Basel 2011 Expedition, M. Brancucci, M. Geiser, D. Hauck, Z. Kraus, A. Phantala & E. Vongphachan” (CLH, NMB); 2 exs., “Laos Svannakhet prov., Phou Xang He NBCA, ca. 5 km SW Ban Pa Phaknau, 250–400 m 17°00'N / 105°38'E, 31.V.-6.VI.2011”, “NHMB Basel 2011 Expedition, M. Brancucci, M. Geiser, D. Hauck, Z. Kraus, A. Phantala & E. Vongphachan” (NMB); 2 exs., “Laos Bolikhamsay prov., Nam Kading NPA research training center near Ban Phon Kham, 18°20'N / 104°08'E, 250 m, 23.-29.V.2011”, “NHMB Basel 2011 Expedition, M. Brancucci, M. Geiser, D. Hauck, Z. Kraus, A. Phantala & E. Vongphachan” (NMB); 2 exs., “Xayaboury Prov., Xayaboury env., 19°13'N, 101°42E, 300 m, 27.-30.VI.2010, leg. D. Hauck” (NMB); 1 ex., “Laos Umg. Vientiane III.-VI.1963” (NMB); 20 exs., “ATTAPEU prov., Annam Highlands Mts., Dong Amphan NBCA, ca. 1160 m, NONG FA [crater lake] env., 15°05.9'N, 107°25.6'E, 30.iv-6.V.2010, J. Hájek leg.” (NMPC); 1 ex., “Pak Lag, 13.VIII.1918 V.de Salvara leg.” (MNHN); 1 ex., “Luang Prabang I.1917 V.de Salvara” (MNHN); 3 exs., “Ban Mong 15.XII.1917 V.de Salvara” (MNHN); 2 exs., “Phongsaly Prov., Phongsaly env., 1500 m, 21°41'N 102°06'E, 28.V.-20.VI.2003, M. Brancucci leg.” (NMB); 4 exs., “Phongsaly Prov. Ban Hatsa, 550 m, 21°44'N 102°12'E, 9.V.+17.VI.2004, M. Brancucci leg.” (NMB); 8 exs., “Phongsaly Prov., Ban Sano Mai, 1150 m, 21°44'N 102°12'E, 19.V.-26.V.2004, M. Brancucci leg.” (NMB); 3 exs., “NE Laos, Houa Phan prov., 20°13'N 104°00'E, Phou Pane Mt., 1.-16.VI.2009, 1350–1500 m, M. Brancucci leg.” (NMB); 2 exs., “Xieng Khouang prov., 19°03'N 103°24'E, Ban Thaviang env., muddy puddle, 500–600 m, 19.V.2010, M. Geiser leg.” (NMB); 1 ex., “N-Laos, Louangphrabang Prov., 19°53'N 102°09'E, Khan river, 300 m, V.Kuban leg.” (NMB); 1 ex., “N-Laos, Louangphrabang Prov., 21°09'N 101°19'E, Namtha, 900–1200 m, 5.-31.V.1997, V.Kuban leg.” (NMB); 1 ex., “Boloikhamxai Prov., 70 km NEE Vientiane, 150 m, 18°16'N 103°11'E, 27.-30.IV.1997, V.Kuban leg.” (NMB); 1 ex., “Vientiane Prov., Lao Pako env. 55 km NE Vientiane, 1.-4.V.2004, J. Bezděek leg.” (NMPC); 1 ex., “C-Laos, Bolikhamsai Province, Ban Nape – Kaew Nua Pass, 18.IV-1.V.1998, alt. 600 m, 18°22.3 N 105°09.1 E, M. Strba & R. Hergovits leg.” (CLH); 1 ex., “N Laos, Vientiane Prov.,Vang Vieng, 300 m, 18°55'23'N 102°26'55'E, 1.-15.V. & 1.-6.VII.2001, J.Koubac leg.” (NMB); 1 ex., “CE Laos, Boli Khan Xai prov., 18°21'N 105°08'E, 8 km NE Ban Nape, 600 m, 1.-18.V.2001, V. Kuban leg.” (NMB); 20 exs., “Annam Laos”, “Sandracottusmixtusab.crucialis Rég. Dr. Guignot det. 1956” (TDMB). • **Thailand**: 2 exs., “Siam Kra Ding 12 III 56 RE Elbel” (USNM); 7 exs., “Siam 17 V 53 RE Elbel”, “Pek-chong Sikiu-kerat Nong Min” (USNM); 7 exs., “Thailand 27 VI 54 REElbel”, “Sakon Nakhon, Muang Sakon Nakhon, Phu Phan” (USNM); 24 exs., “Nakhon Ratchasima Province, Sakaerat Biosphere Reserve, King Cobra Cave, Thailand Coords: 14° 30.536’ N, 101° 55.921’ E, 362 m a.s.l., 27 Oct 2013, W. Atthakor leg., Sakaerat Expedition, lead by Professor Somsak Panha of Chulalongkorn University” (CSUT); 6 exs., “THAILAND Kanchanaburi Prov. Sai Yok N.P. – Pools on road, 23.VII.1996 leg. P. Mazzoldi“ (CPM); 17 exs., “Thailand, Mukdahan LL 2000, Phu Pha Thoep N.P. Small pools on dry stream bed, Mazzoldi P. leg. (23)” (CPM); 1 ex., “Chiang Mai prov. 35 km NW of Muang Ngai, 18° 40’ N 98° 42’ E, 10.I.2006, S. Bečvář S. & R. Fouque leg.” (CJS); 2 exs., “NE Thain Nan district, Ban Pha Khap, 15.-20.V.1992, Pacholatko leg.” (NMW); 2 exs., “NW-Thailand, Chiang Mai, Soppong-Pai, 1800 m, 1.-8.V.1993, Pacholatko & Dembicky leg.” (NMW); 4 exs., “NW-Thailand, Chiang Mai (Zoo), 9.-16.V.1988, at light, Malicky leg.” (NMW); 1 ex., “NW-Thailand, Chiang Mai, 10.-17.V.1989, Malicky leg.” (NMW); 2 exs., “Pang, 300 m, 19°55'N 99°12'E, D.Král leg.” (NMB); 4 exs., “NW-Thailand, Mae Hong Son, Ban Huai Po, 1500 m, 8.-17.V.1992, S.Bily leg.” (NMB); 16 exs., “NW-Thailand, Mae Hong Son, Ban Huai Po, 1600–2000 m, 9.-16.V.1991, J.Horák leg.” (NMB, NMW); 1 ex., “NW-Thailand, Mae Hong Son, Ban Huai Po, 1700 m, 24.-30.VI.1993, Schneider leg.” (NMW); 1 ex., “NW-Thailand, Mae Hong Son, Ban Huai Po, 1600–2000 m, 9.-16.V.1991, Pacholatko leg.” (NMB); 1 ex., “Lansang n.g., Thanon Thong Chai, 500 m, 18.-24.IV.1991, 16°48'N 98°57'E, D.Kral & V.Kuban leg.” (NMB); 1 ex., “Thanon Thong Chai, Chiangdao, 19°24'N 98°55'E, 600 m, 10.-16.V.1991, D.Kral & V.Kuban leg.” (NMB); 1 ex., “Lansang NP, 18.-24.IV.1991, 500 m, 16°48'N 98°57'E, D. Král leg.” (NMPC); 1 ex., “Siam”, “Gebrüder W. Müller Vermächt. 1909” (SMTD). • **Malaysia**: 1 ex., “West Malaysia, Kuala Lipis, small forest pool in rubber plantation, 15.IV.1997, Balke & Hendrich leg.” (CLH); 3 exs., “Kedah Peak, 3200 feet, XII.1915”, “ex. F.M.S. Museum B.M. 1955-354” (NHMUK); 2 exs., “West Malaysia, Penang, Botanical Garden, 27.I.1992, M.Jäch leg.” (9) (NMW). • **Vietnam**: 1 ex., “Vietnam 19.05.2007 Quang Tri Da Krong NP near headquarter lux Csorba leg.” (CHF); 2 exs., “Vietnam N, Quang Binh prov. 1 km N of Cha Lo, 400 m Vitenam-Laos border area 17°41'22´´N 105° 45'45´´E, L. Dembicky leg., 11.-24.iv.2010” (NMB); 4 exs., “Pa-kha 4914”, “Indochine Coll. Dussault” (NMB); 5 exs., “N Vietnam, Sa Pa, 1530 m, 25.-9.VI.1991, J. Strnad leg.” (NMB); 1 ex., “S Vietnam, Saigon, jardin botanique [botanical garden], octobre 1870, A. Krempf leg.” (MNHN); 3 exs., “Cuc Phuong (170 m) Ninh Bin Prov. (N-Vietnam) 10. vii.1997, S. Nomura leg.” (NMST); 1 ex., “Tonkin Occ. Rég. De Hoa Binh R.P. A de Cooman 1919” (MNHN); 1 ex., “Sikkim, Kurseong, R.P. Bretaudeau 1894” (MNHN); 1 ex., “Chasseurs indigènes de ta-Tsién-Loù R.P. Dejean, 1901” (MNHN); 4 exs., “Tonkin Backan P. Lemée 1907-08” (MNHN); 8 exs., “Tonkin Backan P. Lemée, 1908” (MNHN); 1 ex., “Tonkin Occ., Env. de Hoa Binh, R.P. A. de Coomann, 1919” (MNHN); 5 exs., “S-Vietnam, 12 km N Dalat, Lang Bian, 28.-30.IV.1994, Pacholatko & Dembicky leg.” (NMW); 1 ex., “S Vietnam, 16 km N Dalat-Ankroat, 1400 m, 12°05 N 108°24 E, 15.IV.1995, Pacholatko & Dembicky leg.” (NMW); 1 ex., “Tonkin, Chaba, 27.VII.1917, Jeanvoine leg.” (MNHN); 8 exs., “Hoa Binh Tonkin A.deCooman leg.” (MNHN); 10 exs., “Lac Tho Tonkin, A.deCooman leg.” (MNHN); 1 ex., “Hanoi 29.III.1917 V.de Salvara” (MNHN); 1 ex., “Hoa Binh I.1917 V.deSalvara” (MNHN); 1 ex., “N-Vietnam, Shonla [Son La], 9.X.1991, Murzin leg.” (NMW); 1 ex., “N-Vietnam, Ma Da, 27.XII.1990, Murzin leg.” (NMW); 1 ex., “Hoang Lien Son Prov., Sa Pa, 11.-19.VI.1990, M. Dvorák leg.” (NMPC); 2 exs., “S Vietnam, Lao Cai Prov., Sa Pa district, 22°20'48.1´´N, 103°47'45.2´´E, 1690 m, at light, 24.-25.VIII.1998, leg. A. Kun” (TDMB); 1 ex., “S Vietnam, Sa-Pa, Hoang Lien Son, 11.-19. VI.1990, Mir. Dvořák leg.” (CJS); 1 ex., “N-Vietnam, Tam Dao, V.-VI.1990, Pich Richard leg.” (NMB); 1 ex., “N-Vietnam, Tam Dao, 20.-28.VI.1990, S.Brantlova leg.” (NMB); 1 ex., “Tam Dao, 8.V.1990, M.Dudycha leg.” (CJS). • **Indonesia**: 1 ex., “Java” (NMB); 1 ex., “Java Cote Sud Salatri” (MNHN); 1 ex., “Java”, “Zimmermann det.” (DEI); 9 exs., “Java”, “Prov. Pasuruan, Kalipari, 300–500 met., W. Doherty, 1891” (MNHN); 3 exs., “Java occidental, Mons. Tjikorai, 4000 1852”, “H. Fruhstorfer” (MNHN); 2 exs., “Java occ. Toegoë 1902” (MNHN); 1 ex., “Jav. Occ. G. Lamboreth, 1902” (MNHN); 1 ex., “Java Occ., Preanger [Parahyangan or Priangan] Tac Prau Ex. Musaeo van Lansberge” (MNHN); 1 ex., “Java Mts Kawie J.B. Lediou 1898” (MNHN); 2 exs., “Java Ex Museo Thorey” (MNHN); 1 ex., “Java Occ, G. Lambreth, 1908“ (MNHN); 2 exs., “Java Schaum” (MNHN); 1 ex., “Java Coll. Le Moult” (MNHN); 6 exs., “E Java 50 km S Surabaya, Tretes, Kekek Bodo WF, 20.IX.1995, Schillhammer leg. (1)” (NMW); 1 ex., “Java Japara I.1933” (MNHN); 1 ex., “Karanggandoel Banjoemas K. Benner 1922 Muzeum Buitenzorg” (DEI); 1 ex., “Java” (NMW); 1 ex., “E Java, Bajoekidoel b. Banjoawangi 1930 Lucht leg.” (MNHN); 1 ex., “Java Coll. Boucard” (MNHN); 2 exs., “NE Java Baluran NP 600 m 16.-19.IV.1996 R.Zajicek leg.” (NMB); 2 exs., “Java Gebrüder W. Müller, Vermächt. 1909“ (SMTD); 1 ex., “East Java Pare 10.III.1949 W.J.M.Vestjens leg.” (ANIC); 1 ex., “Bali, Bedugul 3 km NE Candi Kuning, 1320 m, 11.VII.1991, Balke & Hendrich leg. (BA 8)” (CLH); 1 ex., “Holländisch Indien”, “Zimmermann det.” (DEI); 1 ex., “Sumatra”, “Sandracottushunteri Zimmermann det.” (ZSM); 1 ex., “Sumatra, Balighe, X.90-III.91, E. Modiglioni” (MNHN); 1 ex., “Sumatra, Fort de Kock II.1925, native collector” (MNHN); 1 ex., “Sumatra 1873” (MNHN); 1 ex., “Sumatra Medan” (MNHN); 4 exs., “Sumatra, Dolog Merangir, 8.V.-10.VI.1983, E.W.Diehl leg.” (NMB); 1 ex., “Sumatra, Dolog Merangir, 10.-12.V.1981, E.W.Diehl leg.” (NMB); 3 exs., “NE Sumatra, Medan, Dr. Marx leg.” (ZSM); 4 exs., “Sumatra O.K Medan 19.9.1921 J.B. Corporaal”, “Museum Paris 1902 J.B. Corporaal” (NMB); 3 exs., “Sumatra Medan J.B. Corporaal”, “Coll. A. Zimmermann” (ZSM); 1 ex., “Sumatra Palembang” (ZSM); 1 ex., “Sumatra, coll. Martin” (ZSM); 3 exs., “Sumatra Padang Fandjang, 800 m, 1.trim. 1896, Kannegieter“ (ZSM); 2 exs., “Höllandisch Indien” [Indonesia] (ZSM); 4 exs., “Sumatra Palembang, Knappert leg.” (NMW); 1 ex., “Sumatra Monte Battak [Bukit Batak] ex. coll. Fruhstorfer” (MNHN).

#### Remarks.

Specimens with an absent subapical transverse band were described as var.crucialis Régimbart, 1899. Vazirani (1969) described his *S.manipurensis* from Assam based on specimens with more extensive black markings of the elytra. When studying the only male paratype of *S.manipurensis* available to us, we were not able to detect any morphological differences in the structure of the median lobes of *S.manipurensis* and *S.hunteri*. The colour pattern of the elytra of the paratype can also be found within the range of variation in *S.hunteri*. We therefore consider *S.manipurensis* synonymous with *S.hunteri*.

#### Redescription.

Body broad oval, shiny, testaceous with black markings. Ventral side completely dark brown to black, legs ferrugineus brown to testaceous.

Head testaceous with posterior part and eye margin black, shiny (Fig. 7). Surface sculpture consisting of very small dense punctures and of larger, much sparser ones, particularly numerous on frons. Clypeal grooves, punctures alongside eyes and transverse depression beside eyes distinctly impressed, punctures large and coalescent. Antennae testaceous; antennomeres slender, antennomere V 3.5× as long as broad.

Pronotum testaceous with median black marking reaching from posterior to anterior margins (Figs 7, 53); broad on posterior, distinctly narrower on anterior margin. Surface shagreened with dense punctation; punctures medium-sized. Anterior and lateral puncture lines with dense coalescent punctures, becoming sparse towards middle, and lacking in middle of anterior margin. Posterior puncture line distinctly visible only at sides, towards middle punctures become superficial and transformed into long wrinkles.

Elytra testaceous to ferrugineus brown with black markings consisting of three transverse bands: broad antemedian, subapical, and apical; suture with narrow black frame, and puncture lines marked by black spots (Figs 9, 53). Epipleura testaceous to ferrugineus brown. Surface very slightly and superficially shagreened and covered with double punctation; denser smaller punctures and much sparser larger ones. Puncture lines with isolated groups of five or six medium-sized punctures; discal row almost complete and strongly impressed. Sutural puncture line marked only by few punctures along suture.

Ventral side dark brown. Legs, particularly fore and mid legs testaceous, hind legs ferrugineus brown to dark brown (Figs 9, 53). Prosternal process short and broad, 1.7× longer than broad, rounded apically, and finely but distinctly sculptured. Posterior border triangular, narrowly ended. Whole surface very superficially shagreened and finely punctured. Metatibia with numerous and dense small punctures on basal half. Ventrites II–VI shagreened, longitudinally wrinkled on lateral parts, densely covered with very small punctures and larger sparser ones. Posterior margins rounded, bordered with some large and coalescent punctures along margin.

Measurements: TL = 12.5–15.0 mm, TL-h = 11.4–14.0 mm, TW = 8.3–8.8 mm.

♂. Protarsomeres I–III strongly enlarged with three larger suckers and numerous smaller one. Mesotarsomeres I–III with two rows of small suckers. Median lobe of aedeagus, in ventral view, broad, nearly parallel-sided on whole length, lobes narrowly rounded at apex (Fig. 18a). Parameres slightly longer than median lobe, broad and pointed at apex (Fig. 18b).

♀. Similar to male, tarsi not enlarged. Microsculpture of ventrite VI as in male.

#### Differential diagnosis.

The species is clearly distinguished from most other Indomalayan species of the genus by its characteristic colouration. From the dorsal colouration *S.hunteri* is near to *S.chevrolati* from the Lesser Sunda Islands which is generally smaller and more elongated. Furthermore, both species can be separated by the shapes of their median lobes.

#### Distribution.

**Figure 30.**
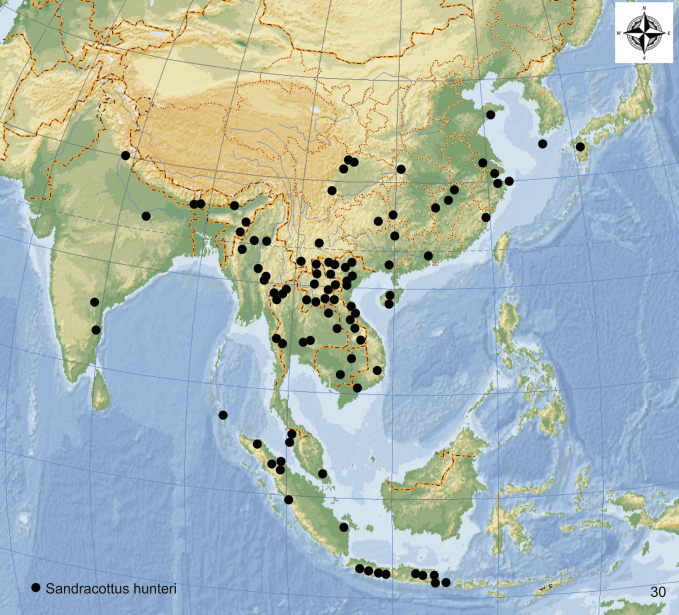
Distribution of *Sandracottushunteri*, the most widespread species of the genus.

The most widespread and common species of the genus. India: Andhra Pradesh, Nagaland, Tamil Nadu, Uttarakhand, Uttar Pradesh, West Bengal, and Andaman and Nicobar Islands (Ghosh and Nilsson 2012; Ghosh and Gupta 2012); Nepal, China (Jäch et al. 2012), South Korea (Jeju-do Island) (Park et al. 2009; Lee et al. 2014), Japan (old record from Higo Province, Sharp 1884), Myanmar, Cambodia, Laos, Vietnam, West Malaysia, Indonesia (Sumatra, Java, Bali) (Fig. 30). The recent Indian records from the states Kerala, Karnataka, Maharashtra, and Tamil Nadu seems doubtful as the photograph in Deb and Subramanian (2023) clearly shows *S.festivus* and its median lobe, not *S.hunteri*. Specimens were collected from near sea level to 2900 m a.s.l.

#### Habitat.

*Sandracottushunteri* inhabits a broad variety of pools, puddles, ditches, and swamps of different sizes, shaded and exposed (Alarie et al. 2023). In Laos numerous specimens were collected by local collectors in pools in the littoral zone of a lake (Figs 37, 38). In Thailand, near Salawin NP, *S.hunteri* was found in open and muddy puddles made by elephants (Fig. 40) and near the King Cobra Cave at Sakaerat Biosphere Reserve, in a large rest pool of a rocky stream bed enriched with decaying leaves and twigs (Fig. 41). In Myanmar *S.hunteri* was collected from roadside pools on a muddy limestone bed with decomposing leaves (Alarie et al. 2023). *Sandracottushunteri* also occurs in cultivated areas and is not restricted to forested sites. On Bali the species was collected several times in smaller pools of intermittent streams with decomposing leaves (Suprayitno et al. 2017). The species was attracted to light. All instar larvae were recently described by Alarie et al. (2023).

**Figures 31–36.**
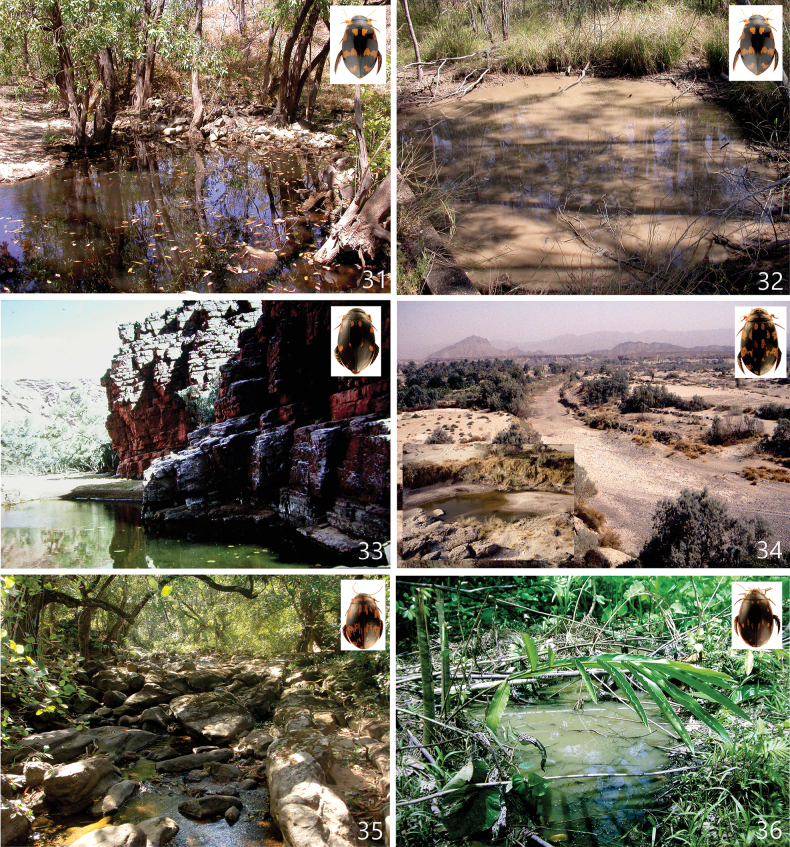
Habitats of *Sandracottus***31** Australia, Northern Territory, 50 km S Adelaide River, creek at Scenic Route (NT 9) and **32** South Queensland, Winfield, Winfield Road, rest pool (QLD 54), habitats of *S.bakewelliibakewellii* (photos: L. Hendrich) **33** Northern Territory, Ormiston Gorge, habitat of *S.bakewelliiguttatus* (photo: M. Baehr) **34** Pakistan, Sistan va Baluchestan Prov., Pir Sohrab env., rest pool in wadi, habitat of *S.dejeanii* (photo: J. Hájek) **35** India, southern Madhya Pradesh, Hoshangabad District, Pagara–Pachmarhi road, ca 5 km NNE Panchmarhi, Panar Pani, habitat of *S.dejeanii* and *S.festivus* (photo: M. Jäch) **36** Indonesia, Irian Jaya, Nabire, Nabire-Ilaga track, km 62, habitat of *S.femoralis* (photo: L. Hendrich).

**Figures 37–42.**
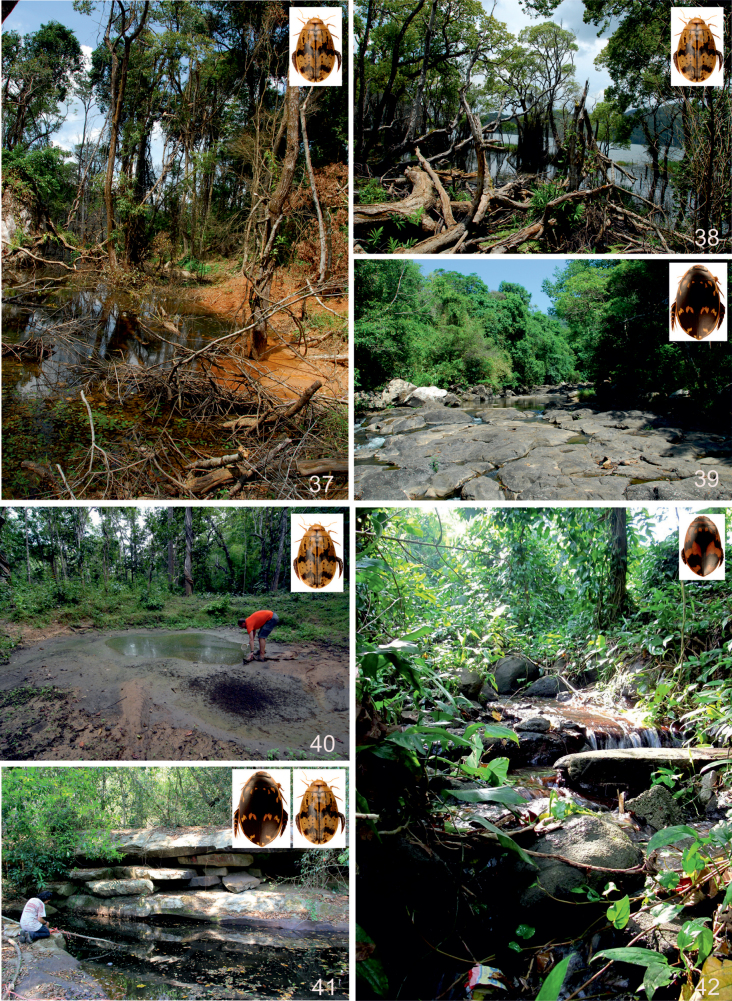
Habitats of *Sandracottus***37, 38** Laos, Attapeu prov., Annam Highlands Mts., Dong Amphan NBCA, Nong Fa [puddles near lake shore] and lake, habitat of *S.hunteri***39** Laos, Katamtok, rest pools along a rocky river bed, habitat of *S.maculatus* (photos: J. Hájek) **40** Thailand, near Salawin NP, open and muddy puddle made by elephants (photo: J. Šťastný), and **41** Sakaerat Biosphere Reserve, rocky pool in an otherwise dry stream bed, habitats of *S.hunteri* and *S.maculatus* (photo: W. Atthakor) **42** Philippines, Luzon, Pampanga Prov., Bano San Juan, Mt. Arayat NP, spring creek, habitat of *S.insignis* (photo: H. Freitag).

### ﻿ Sandracottus insignis

Taxon classificationAnimaliaColeopteraDytiscidae

(Wehncke, 1876)

1113CD62-91B6-5D00-858C-62DFB6C9FB47

Figs 8 , 19 , 28 , 42 , 55

Hydaticus insignis Wehncke, 1876: 194 (type locality Philippines, “Insel Luzon”); Sharp 1882: 687 (descr.).
Sandracottus insignis (Wehncke, 1876); Régimbart 1899: 338 (tax., descr.); Freitag et al. 2016: 8 (check list); Hájek and Nilsson 2024: 91 (cat.).
Hydaticus baeri Régimbart, 1877: 99 (type locality Philippines, “Manille”); Zimmermann 1920: 234 (syn., cat.).
Sandracottus ornatus Sharp, 1882: 689 (type locality Borneo); Vazirani 1977: 84 (stat. nov.).
Sandracottus insignis ssp. ornatus Sharp, 1882; Vazirani 1977: 84 (cat.); Hájek and Nilsson 2024: 91 (cat.) (syn. nov.).

#### Type material of *Sandracottusinsignis*.

***Lectotype*** (herewith designated): Female: “Semper” [white handwritten label with black fram]; “Luzon” [handwritten yellowish label], Ex. Coll. E. WEHNCKE Acq. 1884” [printed white label], “Lectotype Hydaticusinsignis Wehncke Hendrich & Brancucci des. 2010” [red printed label] (MNHN). Examined.

#### Type material of *Sandracottusbaeri*.

***Lectotype*** (herewith designated): Male: “Manille Baer”, “Typus”, “Type”, “Sandracottusbaeri Rég.”, “Lectotypus Hydaticusbaeri Brancucci & Hendrich07” [red printed label], “Sandracottusinsignis (Wehncke) det. M.Brancucci & L.Hendrich07” [white printed label] (MNHN). ***Paralectotypes***: 2 males, “Manille Baer”, “Typus”, “Type”, “Sandracottusbaeri Rég.”, “Paralectotypus Hydaticusbaeri Brancucci & Hendrich07” [red printed label], “Sandracottusinsignis (Wehncke) det. M.Brancucci & L.Hendrich07” [white printed label] (MNHN). Examined.

#### Type material of *Sandracottusornatus*.

***Holotype***: Female: “Borneo 986 ornatus”, “Type H.T.”, “Sharp coll. 1905-313”, “Sandracottusinsignis (Wehncke) Hendrich det. 2008” (NHMUK). ***Paratype***: Female: “Borneo 986 1905-313”, “Sandracottusinsignis (Wehncke) Hendrich det. 2008” (NHMUK). Examined.

#### Remarks.

The type locality of *S.ornatus*, “Borneo”, needs to be confirmed. *Sandracottusinsignis*/*ornatus* has never been reported from Borneo again. However, it cannot be ruled out that the species occurs there, as some dytiscids that occur in the south of the Philippines have also been reported from the north of Borneo. From what is known so far, *S.insignis* appears to be endemic to the Philippines.

#### Additional material.

**(10 specimens)**: • **Philippines**: 1 ex., “Balabac” [handwritten label], “ornatus Shrp” [handwritten label] (MNHN); 1 ex., “Montalban” [Luzon], “Nr. 6 Fld.-H.” [printed label], “baeri Régb. Insignis Sharp” [handwritten label by Régimbart] (MNHN), 2 exs., “Mindanao” (MNHN); 2 exs., “Luzon, Los Banos, Mt. Makiling [above the Botanical Garden, 180 m] (3), 13.XI.1992, H. Schillhammer leg.” (NMW); 1 ex., “Luzon, Pampanga Province, Bano San Juan, Mt. Arayat NP, spring creek, bolders, root packs, riffle, 37 m, 15°10'21.62"N, 120°45'47.78"E, 31.X.2009, H. Freitag leg.” (ZSM); 1 ex., “Polillo [Polillo Islands, Luzon], Taylor” (ZSM); 1 ex., “Palawan, Taytay [10°50'N 119°30'E], W. Schultze leg.” (SMTD).

#### Redescription.

Body broadly oval, shiny, testaceous with black markings. Ventral side completely dark brown to black, fore and mid legs testaceous, hind legs ferrugineus brown.

Head testaceous with posterior part dark brown as well as two dark brown, oblique, elongated patches on frons (Figs 8, 55). Surface finely microreticulate, particularly on anterior half, just shagreened on rest of surface. Punctation consisting of dense and very small punctures and of larger, much sparser ones, the latter particularly numerous on frons. Clypeal grooves, punctures alongside eyes and transverse depression beside eyes distinctly impressed, punctures large and coalescent. Antennae testaceous; antennomeres slender, antennomere V 4× as long as broad.

Pronotum testaceous with long and broad dark brown band posteriorly and narrow dark brown band anteriorly (Figs 8, 55). Surface sculpture shagreened with dense punctation; punctures medium-sized. Anterior rows of punctures with dense, strong, and coalescent punctures, shortly interrupted in middle. Lateral puncture lines broad; punctures medium-sized and coalescent. Posterior row limited to few punctures at latero-basal quarter.

Elytra testaceous with black markings leaving four angulous basal testaceous spots, two large postmedian as well as a preapical and an apical testaceous patch (Figs 8, 55). Epipleura testaceous. Surface microreticulate, meshes very small but strongly impressed. Punctation consisting of numerous minute punctures mostly in intersection of meshes and of large sparse punctures regularly spread on whole surface. Puncture lines with groups of medium-sized punctures, discal row almost complete. Sutural puncture line with few small punctures along suture.

Ventral side dark brown. Legs particularly fore and mid legs testaceous, hind legs ferrugineus brown to dark brown. Prosternal process very short and broad, only 1.4× longer than broad, flattened and superficially sculptured; posterior border broadly rounded. Metacoxa very superficially shagreened and finely punctured; punctures small and sparse; Metatibia with numerous medium-sized punctures on whole outer half, somewhat larger proximally. Ventrites II–VI shagreened, densely covered with very small punctures and larger sparser ones. Posterior margins rounded, bordered with short row of coalescent punctures each side of middle.

Measurements: TL = 12.5–13.1 mm, TL-h = 11.4–12.2 mm, TW = 7.8–8.1 mm.

♂. Protarsomeres I–III strongly enlarged with three larger suckers and numerous smaller ones. Mesotarsomeres I–III with two rows of small suckers. Median lobe of aedeagus, in ventral view, broad, apical half more or less parallel-sided, each part narrowly rounded apically (Fig. 19a). Parameres also narrow, slightly longer than median lobe, and gently tapered on apical half (Fig. 19b).

♀. Similar to male. Tarsi not enlarged. Microsculpture on ventrite VI as in male.

#### Differential diagnosis.

The species is clearly distinguished from most other Oriental species of the genus by its characteristic colouration. From the dorsal colouration *S.insignis* is near to the Indian *S.festivus* which is generally larger and more elongated. Furthermore, both species can be separated by the shapes of their median lobes.

#### Distribution.

Philippines: Palawan, Mindanao, Luzon. The record from Borneo seems to be doubtful (Fig. 28). Specimens were collected between 40 and 200 m above sea level.

#### Habitat.

The three specimens at Mt. Makiling were collected in an exposed roadside ditch surrounded by mature second growth lowland rainforest (H. Schillhammer pers. comm. 2005). The single specimen from Bano San Juan at Mt. Arayat NP was collected among root packs in a shallow bay of a slow flowing creek, partly shaded by second growth vegetation (Freitag pers. comm. 2012) (Fig. 42). The larvae are still unknown.

#### Conservation.

A rare species, probably associated with the declining primary lowland rainforests in the Philippines (see Indomalayan species *S.bizonatus*, *S.femoralis*, *S.maculatus*, and *S.rotundus*). According to the present knowledge it is an endangered species (Freitag et al. 2016). It is recommended to be listed in the next IUCN red list.

### ﻿ Sandracottus jaechi

Taxon classificationAnimaliaColeopteraDytiscidae

Wewalka & Vazirani, 1975

3D613366-4A6D-5C14-BE18-3D3EB205158C

Figs 9 , 20 , 29 , 54

Sandracottus jaechi Wewalka & Vazirani, 1975: 114 (Ceylon [Sri Lanka], Nuwara Eliya); Ghosh and Nilsson 2012: 18 (cat.); Hájek and Nilsson 2024: 91(cat.).

#### Type material.

***Holotype***: Male: “Nuwara Eliya, 1800 m, leg. Kruse [ca 1930, Wewalka and Vazirani 1975] Ceylon (Sri Lanka)”, “TYPUS Sandracottusjaechi n. sp. Wewalka & Vazirani 82” [red label] (CGW, later in NMW). ***Paratypes***: 1 female: “Nuwara Eliya, leg. Kruse 1800 m Ceylon [Sri Lanka]”, “Paratypus Sandracottusjaechi n.sp. Wewalka & Vazirani 82” [red label] (NMW); 1 female: “Nuwara Eliya, leg. Kruse 1800 m Ceylon [Sri Lanka]”, “Paratypus Sandracottusjaechi n. sp. Wewalka & Vazirani 82” [red label] (NHMUK). Examined.

#### Redescription.

Body oval, somewhat broadened posteriorly, completely black and shiny. Ventral side and legs completely black (Figs 9, 54).

Head black. Surface shiny, very superficially shagreened, covered with small and very dense punctures and of larger much sparser ones, the latter more numerous on frons. Clypeal grooves and punctures alongside eyes marked, punctures medium-size and coalescent. Both antennae lacking in holotype.

Pronotum black, sides not margined. Surface very slightly but distinctly shagreened, with very dense punctation; punctures small, less impressed than on head. Anterior puncture line broadly interrupted in middle, punctures large and strongly coalescent. Posterior puncture line with large and coarsely impressed punctures on middle of each side, building distinct wrinkles.

Elytra black, shiny. Epipleura black. Surface distinctly shagreened and covered with double punctation; smaller punctures with very small and dense punctures, larger one with much more sparser ones. Puncture lines with groups of medium-sized punctures mostly grouped by five or six punctures; discal row almost complete and strongly impressed. Sutural puncture line incomplete, marked only by few punctures along suture.

Ventral side black. Legs black. Prosternal process almost flat, short and broad, lanceolate, 1.4× longer than broad, flattened and finely but distinctly sculptured; posterior margin broadly rounded. Metatibial spurs bifid. Metatibia with sparse medium-sized punctures on whole surface. Setae along posterior margin of middle femora sparse and ~ 2/3 of the width of mesofemora at the base. Ventrites II–VI very superficially shagreened, distinctly longitudinally wrinkled on whole lateral parts, densely covered with very small punctures and with very large sparser ones. Posterior margins rounded, deeply bordered with a row of large and coalescent punctures on the middle of each side along the margin. Outer margin of metaventral wings curved. Metacoxal lines short, not reaching apices of metacoxal processes.

Measurements: TL = 14.4–15.0 mm, TL-h = 13.5–13.6 mm, TW = 8.35–8.8 mm.

♂. Protarsomeres I–III strongly enlarged with three larger suckers and ten numerous smaller ones. Mesotarsomeres I–III with two rows of small suckers. Median lobe of aedeagus, in ventral view, broad, flattened parallel-sided on whole length, lobes broadly rounded at apex (Fig. 20a). Parameres slightly longer than median lobe, broad and pointed at apex (Fig. 20b).

♀. Similar to male. Microsculpture on ventrite VI as in male.

#### Differential diagnosis.

This species can be easily separated from all other species by its completely black dorsal surface and the shape of the median lobe.

#### Distribution.

Sri Lanka, only known from the type locality (Fig. 29).

#### Habitat.

The only three specimens were collected at Nuwara Eliya, a hill resort in the mountains of central Sri Lanka. From German and British botanists of the last century the area was well-known for tropical peatland habitats with many unique and endemic plants (e.g., Keilhack 1915a). Impressive black and white photographs of such peatland pools in central Sri Lanka can be seen in Keilhack (1915b). Today the area is mostly cultivated and drained. The completely black dorsal surface and venter of *S.jaechi* may be an adaptation for woodland or peatland ponds and puddles with dark bottoms, decaying leaves, or sedges but no vegetation.

#### Conservation.

This is a highly endangered if not extinct species. It is by far the rarest species of the genus with a very limited distribution. It is recommended to be listed in the next IUCN red list.

### ﻿ Sandracottus maculatus

Taxon classificationAnimaliaColeopteraDytiscidae

(Wehncke, 1876)

B0D7A09D-232B-508A-842F-E1359BE49671

Figs 10 , 21 , 24 , 39 , 41 , 43 , 44 , 56

Hydaticus maculatus Wehncke, 1876: 196 (type locality “Siam” [Thailand]).
Sandracottus wehnckei J. Balfour-Browne, 1944: 355, replacement name for Hydaticusmaculatus Wehncke, 1876 (objective synonym of Hydaticusmaculatus); Vazirani 1969: 277 (descr., syst.); 1977: 86 (cat.); Nilsson 2001: 84 (cat.).
Sandracottus maculatus (Wehncke, 1876): Sharp 1882: 686 (comb. nov.); Régimbart 1899: 338 (descr.); Zimmermann 1920: 235 (cat.); Ghosh and Nilsson 2012: 18 (cat.); Balke et al. 2017: 146 (faun., habitat); Atthakor et al. 2018: 93 (faunistics, habitat); Hájek and Nilsson 2024: 91 (cat.).
Sandracottus angulifer Heller, 1934a: 280 (type locality Davao, Mindanao, Philippines); Hájek and Nilsson 2024: 91 (cat.) (syn. nov.).
Sandracottus palawanensis Satô, 1978: 41 (type locality Sabang, Palawan, Philippines); Hájek and Nilsson 2024: 91 (cat.) (syn. nov.).
Sandracottus nauticus Sharp, 1882: 690 (type locality Borneo); Régimbart 1899: 339; Zimmermann 1920: 235 (cat.); Hájek and Nilsson 2024: 91 (cat.) (syn. nov.).

#### Type material of *Sandracottusmaculatus*.

***Neotype*** (herein designated): Male, “Schoenich” [?], “Cambodscha” [yellowish handwritten label], “Ex. Coll. E. WEHNCKE Acq 1884” [white printed label], “NEOTYPUS Hydaticusmaculatus L. Hendrich & M. Brancucci des. 2010” [red printed label] (MNHN). Examined.

#### Comments on classification.

It was impossible to find any type material labelled “Siam” among the historical specimens in the NHMUK, MNHN, and ZHMB, so we decided to designate a neotype from Cambodia found in Wehncke´s collection and deposited in MNHN. The neotype is designated to support taxonomic stability, as there are several morphological similar *Sandracottus* species described from the Indomalayan region, and it is paramount for present and future investigations to possess unambiguously characterised name-bearing specimens for all Indomalayan species of the genus.

#### Type material of *Sandracottusangulifer*.

***Holotype***: Male, “Davao Mindanao Baker”, “7251”, “S.angulifer Typus” [red label], “1932 12”, “Staatl. Museum für Tierkunde, Dresden”, “*Sandracottusmaculatus* (Wehncke, 1876) Hendrich & Brancucci det. 2006” (SMTD). Examined.

#### Type material of *Sandracottuspalawanensis*.

***Holotype***: Male, “Sabang, Palawan Philippines July 13, 1977 M. Sâto leg.” [handwritten], “HOLOTYPE Sandracottuspalawanensis M. Satô DET. M. SATÔ 1978” [red label], “*Sandracottusmaculatus* (Wehncke, 1876) Hendrich & Brancucci det. 2010” (NMST). Examined.

#### Type material of *Sandracottusnauticus*.

***Holotype***: Male, “Borneo 987 nauticus”, “Type H.T.”, “Sharp Coll. 1905-313” (NHMUK). “*Sandracottusmaculatus* (Wehncke, 1876) Hendrich & Brancucci det. 2006”. Examined.

#### Additional material.

**(150 specimens)**: • **Cambodia**: 1 ex., “Cambodia Pailin 200 m 11.-16.VI.2008 S. Murzin leg. Coll. Hendrich" (CLH). • **Laos**: 7 exs., “LAOS centr., Kham Mouan Prov. Nakai vill. env. ca 70 km NNE Muang Khammouan, 500–600 m 17°43'N, 105°09'E, 7.-25.V.2002 M. Strba leg./Coll. HENDRICH” (CLH); 1 ex., “LAOS south, Attapeu prov. Bolaven Plateau 18.-30.IV.1999, 15 km SE of Ban Houaykong, NONG LOM (lake) env., N 15°02´ E 106°35´, alt. 800 m, E. Jendek & O. Sausa leg.” (CLH); 1 ex., “Champasak Prov., Ban Nam Touad env. (near Xe Katamtok), village, agricultural lands, old secundary and degraded primary forest, light trapping, 500–800 m, 15°06'N, 106°35'E, 8.-10.VI.2010, leg. M. Geiser & D. Hauck” (NHB); 1 ex., “Northern Vientiane Prov. Vang-Vieng, 300 m, N 18°55.23 E 102°26.55, 10.-15.V. & 1.-6.VI.2001, J. Kolibac leg.” (NMB); 1 ex., “Umg. Vientiane, III.-IV.1963, coll. M. Brancucci” (NMB); 2 exs., “Khammouan Prov., Ban Khoun Ngeun, 200 m, 18°07'N 104°29'E, 24.-29.IV.2001, V. Kuban leg.” (NMB); 8 exs., “Khammouan Prov., Nakai env., 500–600 m, 22.V.-8.VI.2001, 17°43'N 105°09'E, E.Jendek & O.Sausa leg.” (CHH, NMPC); 1 ex., “CHAMPASAK prov., Bolavens Plateau, waterfall ca., 2 km E TAD KATAMTOK, 15°08.1'N, 106°38.8'E, 415 m, 10–12.V.2010, J. Hájek leg.” (NMPC). • **Thailand**: 1 ex., “N-Thailand, Khon Kaen Prov., Si Chom Phu, 220 m, 16°8531N, 102°2526E R. Ohnesorge, IX.2012”, “coll. A. Skale Hof/Germany” (CAS); 1 ex., “Thailand 25 V 52 REElbel”, “Kanchanaburi” (USNM), 14 exs., “Nakhon Ratchasima Province, Sakaerat Biosphere Reserve, King Cobra Cave, Thailand Coords: 14°30.536’ N, 101°55.921’ E, 362 m a.s.l., 27 Oct 2013, W. Atthakor leg., Sakaerat Expedition, led by Professor Somsak Panha of Chulalongkorn University” (CSUT); 1 ex., “N Thailand, Doi Pho Ka N.P. Road from Pua 1 km after Park Hq. m 1400, small pools on rock (24) 2.I.1999 P. Mazzoldi leg.” (CPM); 2 exs., “Siam: MeSong Forest IV.1919 E.J.Godfrey 1920-244” (NHMUK); 2 exs., “E Thailand, Ko Chang White Sands Beach, 10.XII.1990, M.Jäch leg. (12)” (NMW); 1 ex., “Prov. Rayong, Khao Chamao NP, 12. &13.XII.1990, M.Jäch leg. (14)” (NMW); 1 ex., “Tham Sakoen NP, No. 21, 29.-30.XI.2003, 19°23'N 100°38'E, Peregovits, Foldvari, Korosi, Szappanos & Maklari-Kis leg.” (TDMB); 2 exs., “Pak Lag, 14.VIII.1918, Jeanvoine” (MNHN); 17 exs., “Pak Lag 13.VIII.1918 V. de Salvaza” (MNHN); 6 exs., “Pak Lag, 13.VIII.1918 V. De S.”, “Samml. A. Zimmermann” (ZSM); 3 exs., “Pak Lag 18.VII. 1918 no. 2119 V. de S.” (MNHN, ZSM). 2 exs., “Thailand, E Bangkok, Nakhon Nayok: Khlong Maduea, 14°21'17"N, 101°16'22"E, 16.03.2017, leg. H. Shaverdo” (NMW); 3 exs., “Thailand, Phetchabun Province, near Nam Nao National Park, Route 2216 then small road off, in village Khlong Choen, 16°40'29"N, 101°44'01"E 21.03.2017, leg. H. Shaverdo” (NMW). • **Vietnam**: 1 ex., “VietnamN, Quang Binh prov. 1 km N of Cha Lo, 400 m Vietnam-Laos border area 17°41'22´´N 105° 45'45´´E, L. Dembicky leg., 11.-24.iv.2010” (NMB); 1 ex., “Tonkin Env. De Hoa Binh J. Laisi 1902“, “S.maculatus Wke”, “Dr. Régimbart vidit 1905” (MNHN); 1 ex., “Museum Paris Cochinchine” (MNHN); 3 exs., “Hoa Binh Tonkin” (MNHN); 3 exs., “Lactho Tonkin A.de Cooman” (MNHN); 1 ex., “S Vietnam, Saigon, jardin botanique [botanical garden], octobre 1870, A. Krempf leg.” (MNHN); 1 ex., “Hoa Binh (Tonkin) A. de Cooman” (MNHN); 3 exs., "Hoa Binh (Tonkin) A. de Cooman” (MNHN); 2 exs., “Tonkin occ. env. de Hoa Binh 1919 A. de Cooman” (MNHN); 4 exs., “S-Vietnam, Nam Cat Tien NP, 1.-15.V.1994, Pacholatko & Dembicky leg.” (NMW); 1 ex., “Vietnam, Dong NAI, Nam Cat Tien NP, 120 m, 18-IX-1998, leg. L. J. Wang” (CLJW). • **Malaysia**: "E-Malaysia, Sabah Borneo, Mt. Trus [= Trusmadi or Trus Madi] March-April 2010 Local leg.”, “coll. A. Skale Hof/Germany” (CAS); 1 ex., “NE Borneo Sandakan, Coll. Guignot” (MNHN); 1 ex., “Hydaticus sp.? Pinang" [West Malaysia, Penang], “maculatus Wehncke” [handwritten label by Régimbart] (MNHN); 1 ex., “Borneo Sandakan Windrath”, “nauticus Shp” (MNHN), 1 ex., “Borneo, Kinabalu [Sabah], Whitehead, Fry Coll. 1905-100” (NHMUK); 1 ex., “Borneo 987, Sharp Coll. 1905-313” (NHMUK); 3 exs., “Borneo, Sabah, Kampung Pisang, Pisang env., tributary of Kuamut river, 29.VI.1998, J.Kodada & F.Ciampor leg.” (NMW); 1 ex., “Borneo Sabah, Hot Springs, 26.V.1999, Bacovský leg.” (NMPC); 20 exs., “Borneo, Sabah, Sungai Kinabatangan, stream near Danau Blandung Besar, 11.IV.1994, H.K. Lua leg.” (CLH, ZRC); 2 exs., “Sabah, Kinabatangan riv. 8.-15.VI.2003, Uncle Tan´s camp, J.Šťastný leg.” (CJS); 1 ex., “Dindings 96-85” [Malaysia, Perak, Manjung District], “Determined by Dr Régimbart Sandracottusmaculatus v. nauticus Sharp”; 1 ex., “Ex. Coll. E.Wehncke Acq 1864” (MNHN); 3 exs., “West Malaysia, Penang 988, Sharp Coll. 1905-313” (NHMUK); 5 exs., “West Malaysia, Penang, Pascoe Coll. 93-60” (NHMUK). • **Indonesia**: 1 ex., “Ned. Indie Leg. A. Koller”, “Coll. F.C. Drescher” (RMNH); 5 exs., “Sumatra Palembang M. Knappert” (RMNH); 7 exs., “East Kalimantan, Distr. Damai 12.VII.1995 Ngelung Stream Mazzoldi P. leg.” (CPM); 1 ex., “East Kalimantan 12.VII.1995, Distr. Tingai stream near Sembuan, opening in forest – leg. P Mazzoldi” (CPM); 1 ex., “Java Occ. Toeroe 1902”, “Museum Paris ex. Coll. Oberthür 1952” (MNHN); 1 ex., “Java ex. J. Waterstradt 1904” (NMHN); 1 ex., “Java”, “maculatus Wehncke (MNHN); 1 ex., “Malang Java” (MNHN); 1 ex., "Java, Prov. Pasuruan Kalipari, 300–500 m, 1891, W.Doherty leg." (MNHN); 1 ex., "Java, Blume leg." (RMNH); 1 ex., "Java, Muller leg." (RMNH); 4 exs., “Z.-Sumatra, Ranau, 500–700 m, Juli 35” (RMNH); 1 ex., “SW Java, Gautang Bay, III.1937, M.A.Lieftinck leg.” (MNHN); 3 exs., “Sumatra, Palembang” (MNHN); 1 ex., “Sumatra, Dolok Merangir, 8.V.-10.VI.1983, E.W.Diehl leg.” (NMB); 1 ex., “Mentawai Islands, Siberut, 5–50 m, 3.IV.2005, S.Jákl leg.” (NMPC); 2 exs., “Sumatra, Palembang Coll. Peschet” (MNHN); 2 exs., “Sumatra Palembang”, “Samml. A. Zimmermann” (ZSM); 1 ex., “Sumatra Medan Hayek leg. (ZSM). • **Philippines**: 1 ex., “Mindanao Davao XII.1932, Baker leg.” (SMTD); 1 ex., “Mindanao” (MNHN); 1 ex., “Philippines: Luzon, Bicol, Camarines Sur, Lagonoy ca. 13°44'15.7"N, 123°31'16.1"E 09.2019 leg. local collector” (CLW).

#### Remarks.

Specimens from Borneo and Sumatra with reduced yellow basal markings on elytra (habitus nearfemoralis) were described as *S.nauticus* Sharp. In the shape of the median lobe of aedeagus, some specimens from Borneo lies between *S.femoralis* and *S.maculatus* from mainland Asia. Furthermore, we do not have any molecular data for either form, so we here list *S.nauticus* as an objective junior synonym of *S.maculatus*.

#### Redescription.

Body large, broad oval, submatt, only slightly shiny, black with ferrugineus brown markings. Ventral side completely dark brown to black, legs testaceous, hind legs somewhat darker.

Head testaceous with posterior part broadly black leaving free two small testaceous spots on vertex (Figs 10, 56). Surface submatt, shagreened, consisting of dense and minute punctures but not very uniform in size and of larger and much sparser ones, more numerous on frons. Clypeal grooves and punctures alongside eyes marked, punctures medium-sized but coalescent. Antennae testaceous; antennomeres slender, antennomere V 4× as long as broad.

Pronotum black with margins broadly testaceous, submatt (Figs 10, 56). Surface distinctly shagreened, with very dense punctation; punctures medium-sized and distant of only 1–2× their own diameter. Anterior puncture line interrupted in middle; punctures relatively small but coalescent, building wrinkles and becoming sparse towards middle and lacking in very middle of anterior margin. Posterior puncture line with coarse and coalescent punctures in middle of each sides building distinct wrinkles, distinctly smaller and spaced on disc.

Elytra black with testaceous markings consisting of one basal band sometimes reduced to different spots at base, one postmedian band, one preapical short and reduced band, and an apical spot (Figs 10, 56), submatt. Epipleura ferrugineus brown. Surface distinctly shagreened and covered with small and dense punctures as well as with larger and much more sparser ones. Puncture lines with groups of medium-sized punctures mostly grouped by five or six punctures; groups closer together on discal row. Sutural puncture line marked only by few punctures on apical part.

Ventral side dark brown. Legs particularly fore and mid legs testaceous, hind legs ferrugineus brown to dark brown. Prosternal process short and broad, 1.4× longer than broad, flattened finely but distinctly sculptured; posterior margin broadly rounded. Metatibia with sparse medium-sized punctures on whole surface. Ventrites II–VI very superficially shagreened, slightly and longitudinally wrinkled on lateral parts, densely covered with very small punctures and larger sparser ones. Posterior margins rounded, deeply bordered with some large coalescent punctures on middle of each side along margin.

Measurements: TL = 13.4–16.6 mm, TL-h = 13.1–15.2 mm, TW = 8.5–10 mm.

♂. Protarsomeres I–III strongly enlarged with three larger suckers and numerous smaller one. Mesotarsomeres I–III with two rows of small suckers. Median lobe of aedeagus, in ventral view, elongate, parallel-sided on complete length, lobes narrowly rounded at apex (Fig. 21a). Parameres narrow and elongate, and slightly longer than median lobe (Fig. 21b).

♀. Similar to male. Pro- and mesotarsi not enlarged. Microsculpture on ventrite VI as in male.

#### Differential diagnosis.

The species is clearly distinguished from all other Indomalayan species of the genus by its colouration and larger size. It is the largest species of the genus. From the dorsal colouration *S.maculatus* is near to *S.femoralis* which is generally smaller in size (see *S.femoralis*). Furthermore, both species can be separated by the shapes of their median lobes.

#### Distribution.

Cambodia, Laos, Thailand, Vietnam, Malaysia (Sabah), Indonesia (Kalimantan, Sumatra, Java, Siberut) and Philippines (Luzon, Mindanao, Palawan) (Fig. 24). Specimens were collected from approximately sea level to 800 m a.s.l.

#### Habitat.

**Figures 43–45.**
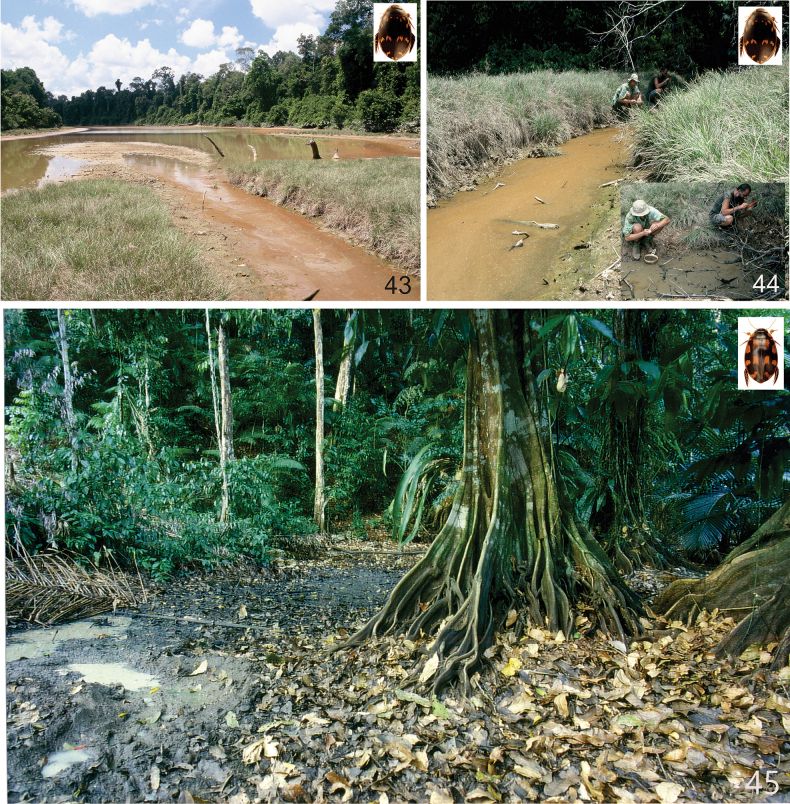
Habitats of *Sandracottus***43, 44** Malaysia, Sabah, Kinabatangan riv. 8.-15.VI.2003, Uncle Tan´s camp, habitat of *S.maculatus* (photos: J. Šťastný) **45** Indonesia, Sulawesi, Togian Islands, Kaldidiri Island near Paradise Island Resort, Babirusa puddles (on the left), habitat of *S.rotundus* (photo: J. Haft).

A widespread but rare species. According to various collectors all recent records are from well vegetated pools, forest pools, or rest pools of intermittent streams and rivers, mainly located in primary rainforest (Fig. 43). At least most habitats are partly shaded and enriched with decaying leaves and twigs (Fig. 44). In Thailand (Sakaerat Biosphere Reserve) the species was collected at the end of the dry season in a rest pond of a rocky stream, with very dark water and accumulated leaf litter. The pond was more or less permanent but with a high fluctuation of water level (Fig. 41). The species was syntopic with *S.hunteri* (Atthakor et al. 2018). In Vietnam, the species was collected in shallow water made by a passing jeep on a path. Syntopically occurring dytiscid species include *Leiodyteskualalipis* Balke, Wang, Bergsten & Hendrich, 2017, *L.nicobaricus* (Redtenbacher, 1867), and *Hydroglyphusorientalis* (Clark, 1863) (Balke et al. 2017). In Laos, *S.maculatus* was collected in a residual pool near the river (Fig. 39). The pool had a diameter of nearly 1 m, depth was ~ 40 cm, bottom with a thick layer of decaying leaves (Hájek pers. comm. 2023).

### ﻿ Sandracottus rotundus

Taxon classificationAnimaliaColeopteraDytiscidae

Sharp, 1882

2E4D6E5D-0A52-58F2-B913-75FFF5D41D1E

Figs 11 , 22 , 27 , 45 , 57

Sandracottus rotundus Sharp, 1882: 688 (type locality “Celebes” [Indonesia, Sulawesi]); Régimbart 1899: 337 (descr.); Zimmermann 1920: 235 (cat.); Hájek and Nilsson 2024: 91 (cat.).

#### Type material.

***Holotype***: Male, “Type H.T.”, “Celebes 985 rotundus”, “Sharp Coll. 1905-313” (NHMUK). Examined.

#### Additional material.

**(80 specimens)**: • **Indonesia**: 1 ex., “INDONESIA, N-Sulawesi vic. Raja Basar b. Moutong, 15 m N 0°29'78” E 121°12'99”, 28.II.2009, river valley (*016*), A. Skale leg.” (CAS); 2 exs., “Minahassa, Celebes” [Minahasa, Sulawesi] (RMNH); 5 exs., “Rosenberg, Toelabollo, Celebes” [Tulabalo, Sulawesi] (MNHN, RMNH); 1 ex., “Indonesia, Celebes” [Sulawesi] (NHMUK); 1 ex., “Indonesia, C-Sulawesi, 45 km SE Palu, 1994, 01°11'S 120°08'E, J. Haft leg. (5)” (NMW); 1 ex., “Indonesia, Sulawesi Utara, Dumoga Bone N.P., XI.1985, Rothamsted light trap, site 1, 200 m, H.Barlov leg.” (NHMUK); 1 ex., “Indonesia, Sulawesi Utara, Dumoga Bone N.P., 17.I.1985, lowland forest 200–300 m”, “R. Ent. Soc. London Project Walace B.M. 1985-10” (NHMUK); 1 ex., “Indonesia, Sulawesi Utara, Dumoga Bone N.P., 6.II.1985, site 5, Tumpah transect, 300 m, J.D. Holloway leg.” (NHMUK); 1 ex., “Indonesia, Sulawesi Togian Islands, Pulau Togian, river in forest south of Wakai, 5.-17.VIII.1987, D.T. Bilton leg.” (CLH); 16 exs., “Sulawesi Togean Islands, Kadidiri Island interior, 30 m, 28.viii.2011, 00 21.531S 121 50.959E (SUL005)” (MZB, ZSM); 50 exs., “Indonesia, C-Sulawesi, Togian Islands, Kaldidiri Island near Paradise Island Resort, 50 m, S 00°21 E 121°50, 12.-15.II.1997, J. Haft leg.” (CLH, CJS, NMB, NMPC).

#### Redescription.

Body broad oval, shiny, testaceous with black markings. Ventral side completely dark brown to black, legs testaceous, hind legs somewhat darker.

Head testaceous with posterior part and broadly so on posterior half alongside as well as two elongate spots on clypeus black, shiny (Figs 11, 57). Surface almost smooth consisting of dense and very numerous punctures of different size and of larger, much sparser ones, particularly numerous on frons. Clypeal grooves, punctures alongside eyes and transverse depression beside eyes distinctly impressed, punctures large and coalescent. Antennae testaceous; antennomeres slender, antennomere V 4.5× as long as broad.

**Figures 46–49.**
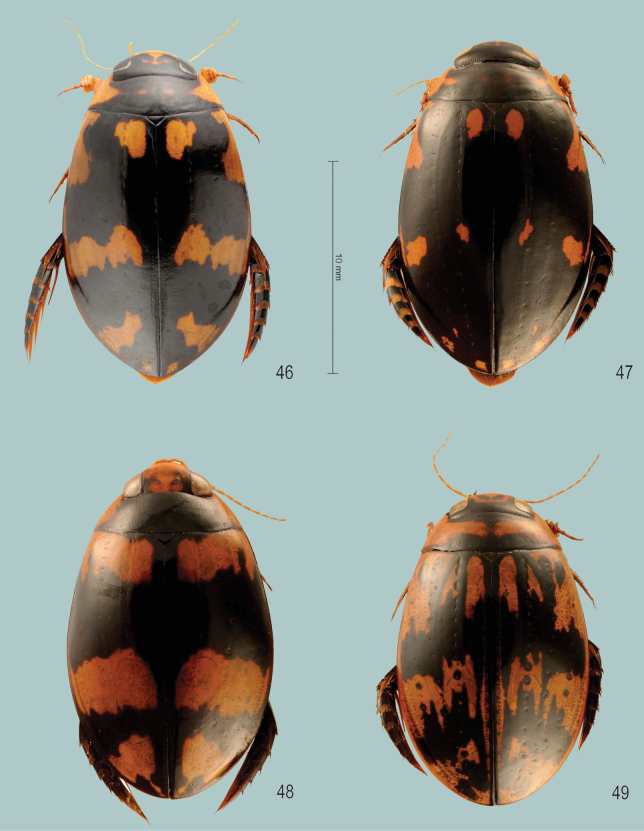
Colouration and habitus of **46***Sandracottusbakewelliibakewellii* (Australia, Northern Queensland, Atherton Tableland, Mareeba) **47***S.bakewelliiguttatus* (Australia, Northern Territory, Ormiston Gorge) **48***S.bizonatus* (Borneo, Sabah, Keningau) **49***S.chevrolati* (Indonesia, Sumba Island, Waingapu).

Pronotum black with broad lateral testaceous markings (Figs 11, 57). Surface very superficially shagreened, almost not visible, with dense punctation; punctures medium-sized mixed with smaller ones. Anterior and lateral puncture lines dense and coalescent, punctures becoming sparse towards middle and lacking in very middle of anterior margin. Posterior puncture line with coarse and coalescent punctures on middle of each side, distinctly smaller and spaced out on disc.

**Figures 50–53.**
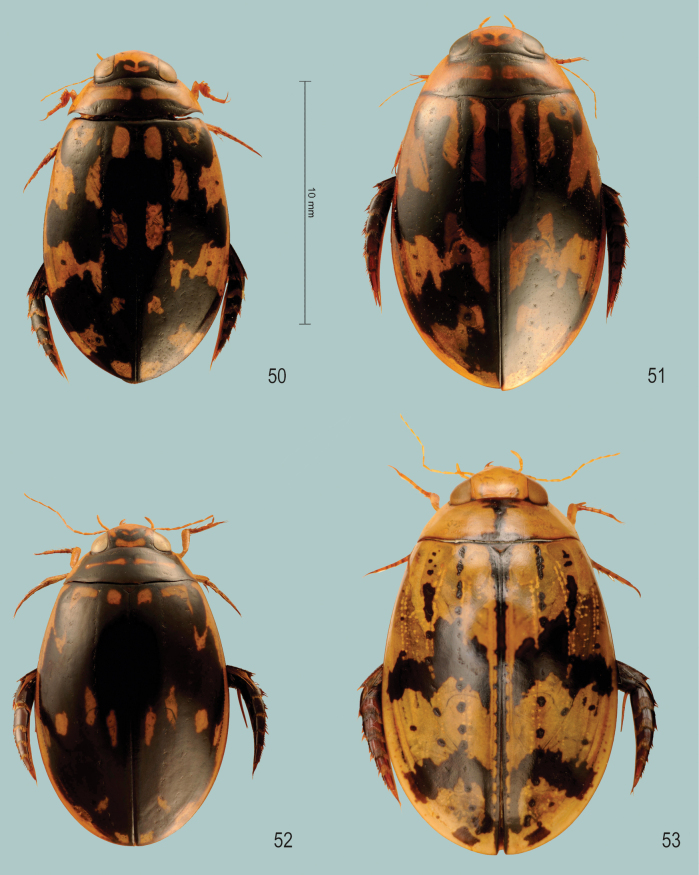
Habitus of **50***Sandracottusdejeanii* (India, Himachal Pradesh, Kangra Valley, Dharamsala) **51***S.festivus* (Sri Lanka, Ratnapura, Sincharaja **52***S.femoralis* (Indonesia, West Papua, Kapupaten Paniai, Nabire) **53***S.hunteri* (China, Central Sichuan).

Elytra black with five testaceous markings, consisting of one basal, two lateral, one just behind middle, and one posterior apical one (Figs 11, 57). Epipleura testaceous to ferrugineus brown. Surface very slightly and superficially shagreened and covered with double punctation, smaller and denser ones as well as larger much sparser ones. Puncture lines with groups of medium-sized punctures mostly grouped by five or six punctures; groups closer together on discal line.

**Figures 54–57.**
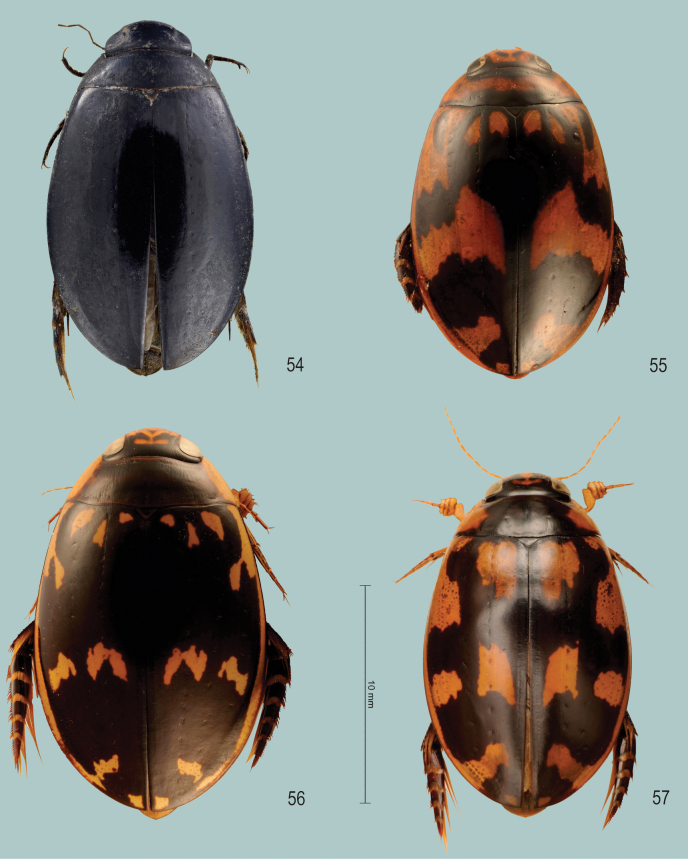
Habitus of **54***Sandracottusjaechi* (holotype, Sri Lanka, Nuwara Eliya) **55***Sandracottusinsignis* (Philippines; Luzon, Los Banos) **56***S.maculatus* (Borneo, Sabah, Sungai Kinabatangan) **57***S.rotundus* (Indonesia, Sulawesi, Togian Islands, Kaldidiri Island).

Ventral side dark brown. Legs particularly fore and mid legs testaceous, hind legs ferrugineus brown to dark brown. Prosternal process short and broad, 1.3× longer than broad, flattened finely but distinctly sculptured. Posterior margin broadly rounded. Whole surface very superficially shagreened and finely punctured. Metatibia with sparse medium-sized punctures on outer half. Ventrites II–VI very superficially shagreened, slightly and longitudinally wrinkled on lateral parts, complete surface densely covered with very small punctures and larger sparser ones. Posterior margins rounded, bordered with some large and coalescent punctures on middle of each side.

Measurements: TL = 12.4–12.8 mm, TL-h = 11.4–11.9 mm, TW = 7.6–7.9 mm.

♂. Protarsomeres I–III strongly enlarged with three larger suckers and numerous smaller ones. Mesotarsomeres I–III with two rows of smaller suckers. Median lobe of aedeagus in ventral view broad, constricted medially, parallel-sided in apical part up to apex, here slightly broadened and broadly rounded (Fig. 22a). Parameres broad and strongly pointed at apex, slightly longer than median lobe (Fig. 22b).

♀. Similar to male, tarsi not enlarged. Microsculpture on ventrite VI as in male.

#### Differential diagnosis.

The species is well distinguished from all other Oriental species of the genus by its colouration and roundish oval body. From the dorsal colouration *S.rotundus* is near to the Australian *S.bakewelliibakewellii* (Figs 1, 46) which is generally more elongated. Furthermore, both species can be separated by the shapes of their median lobes (Figs 12a, b, 22a, b).

#### Distribution.

Indonesia: northern and central Sulawesi including Togian Islands (Fig. 27). Specimens were collected between 30 and 300 m a.s.l.

#### Habitat.

*Sandracottusrotundus* seems to be restricted to stagnant water bodies in primary lowland forests of northern and central Sulawesi and their adjacent islands. All specimens on Kaldiri Island were obtained from muddy forest pools (depths up to 30 cm) and from shallow water of a forest lake not far from the sea. According to Jan Haft (pers. comm. 1998) those pools were frequently used and probably created by Babirusas [*Babyrousatogeanensis* (Sody, 1949)] (Fig. 45). Co-occurring species include the rare *Cybisteraterrimus* Régimbart, 1899, *Hydaticus* species of the *pacificus* group, and some unidentified *Copelatus*.

#### Conservation.

A rare species recorded from a very restricted area in Indonesia. Most probably the species is associated with the declining primary lowland rainforests on the island Sulawesi. It is recommended to be listed in the next IUCN red list.

### ﻿Key to all species of genus *Sandracottus*

**Table:** 

1	Dorsal and ventral side completely black, without any yellowish markings on pronotum or elytra (Fig. 54). A single species endemic to the highlands of Sri Lanka	***S.jaechi* Wewalka & Vazirani, 1975**
–	Dorsal surface with yellowish markings, ventral side black to reddish-brown, and appendages dark brown to reddish brown	**2**
2	Pronotum mainly black, laterally testaceous	**3**
–	Pronotum mainly yellowish, with black margins anteriorly and posteriorly	**6**
3	Pronotum with narrow, testaceous, lateral margins, and/or testaceous, horizontal band, interrupted medially. Elytron black, with several smaller testaceous markings and spots	**4**
–	Pronotum with broad, testaceous, lateral margins. Elytron black, with large testaceous markings	**5**
4	Larger species, TL = 13.4–16.6 mm. Horizontal testaceous band on pronotum vague and almost invisible (Figs 10, 56). Thailand, Laos, Vietnam, Cambodia, Malaysia, Indonesia to the Philippines	***S.maculatus* (Wehncke, 1876)**
–	Smaller species, TL = 11.9–12.6 mm. Horizontal testaceous band on pronotum well developed (Figs 6, 52). Moluccas, New Guinea, Solomon Islands	***S.femoralis* Heller, 1934**
5	Elytron with three large testaceous markings (Figs 2, 48). Endemic to Borneo	***S.bizonatus* Régimbart, 1899**
–	Elytron with five larger testaceous markings (Figs 11, 57). Endemic to Sulawesi	***S.rotundus* Sharp, 1882**
6	Elytron mainly testaceous with black markings consisting of three transverse bands: a broad antemedian, a subapical and an apical one (Figs 7, 53). Larger species, TL = 12.5–15 mm. Widespread in India, China, and Southeast Asia (Fig. 53)	***S.hunteri* (Crotch, 1872)**
–	Elytron mainly black with testaceous markings	**7**
7	Species distributed in Australia	**8**
–	Species distributed in India and Southeast Asia	**9**
8	Elytron black, with large basal, subbasal and apical band testaceous (Figs 1, 46). Northern and eastern coastal Australia	***S.bakewelliibakewellii* (Clark, 1864)**
–	Elytron black, with small basal, subbasal and apical dots testaceous (Fig. 47). Central Australia	***S.bakewelliiguttatus* (Sharp, 1882)**
9	Larger species (Figs 5, 51), TL = 14.7–15.5 mm. Median lobe and parameres as in Fig. 16a, b. India, Sri Lanka	***S.festivus* (Illiger, 1802)**
–	Smaller species, TL = 12.0–13.5 mm	**10**
10	Anterior and posterior black band of pronotum not connected medially. Species with one connected (Fig. 55) or two separated broad testaceous subbasal markings on elytron (Fig. 8), TL = 12.5–13.1 mm. Philippines	***S.insignis* (Wehncke, 1876)**
–	Anterior and posterior black band of pronotum connected medially	**11**
11	Elytron particularly characterised by the presence of several longitudinal testaceous subsutural spots (Figs 3, 49). TL = 13.0–13.5 mm. Indonesia, Lesser Sunda Islands east of Wallace Line, Tanimbar, Timor, SE Sulawesi	***S.chevrolati* (Aubé, 1838)**
–	Elytron black to dark brown with testaceous markings in form of a chessboard, the testaceous markings alternating with the black ones (Figs 4, 50). TL = 12.0–13.0 mm. India, Pakistan, Nepal, SE Iran	***S.dejeanii* (Aubé, 1838)**

## ﻿Discussion

### ﻿Habitats

Just 30 years ago, almost nothing was known about the habitat requirements of these attractive and large diving beetles. Most of the Oriental species were only known from old dusty museum specimens without any habitat information. From time to time, single specimens of *S.hunteri*, *S.chevrolati*, and *S.dejeanii* were offered for sale at insect fairs, collected by anonymous local collectors most probably with light traps. Occasionally, living specimens of *S.hunteri* were offered in aquarist and pet shops via the internet.

The Oriental *Sandracottus*, except *S.hunteri* and *S.chevrolati*, are typical inhabitants of primary or very old secondary growth rain forests which are not, or only slightly, disturbed by human influences, whereas the two subspecies of the Australian *S.bakewellii* and the Indian *S.dejeanii* and *S.festivus* can be found mainly in side-pools or shaded rest pools of intermittent creeks and streams. On the Indian subcontinent single specimens of *S.dejeanii* and *S.festivus* are recorded from all kinds of lentic habitats. The bottom of the water bodies may be stony if it is a residual pool on a rocky riverbed, or sandy or gravelly if it is a rest pool or a sheltered inlet in a small stream, or muddy if it is a forest or woodland pool. In general, the bottom is enriched with a fine detritus of rotten, decaying leaves, twigs, or smaller logs.

It is supposed that the completely black-coloured *S.jaechi* from the highlands of Sri Lanka is restricted to high altitude peatland ponds and mires with dark bottom. The most eurytopic species is *S.hunteri*, inhabiting woodland pools, puddles, and ditches, often rich in emerged and submerged vegetation (Myanmar, west Malaysia, and Thailand), forested and swampy lake margins (Laos), rest pools of intermittent streams (India, Indonesia), and irrigation channels of paddy fields and open flooded meadows (Thailand, Vietnam, China).

For the Southeast Asian species (*S.bizonatus*, *S.insignis*, and *S.maculatus*) the population density seems to be quite low as only small numbers of beetles have been found at one spot. Only in *S.chevrolati*, *S.hunteri*, and *S.rotundus* are aggregations of a dozen or more specimens known (Balke, Hornburg, and Hájek pers. comm. 2015). It is noteworthy that at many localities immature specimens were observed but no larvae. The larvae of most species, except *S.dejeanii* (Vazirani 1971), *S.hunteri*, and *S.femoralis* (Alarie et al. 2023), remain unknown. Some species may be sympatric and sometimes syntopic: In India *S.dejeanii* and *S.festivus* often co-occur, and in Laos, Thailand, and Indonesia *S.hunteri* and *S.maculatus* were collected in the same pools. Co-occurring dytiscid species mainly include various *Hydaticus* sp. of the *pacificus* group and the *fabricii* group, *Copelatus* sp., *Hyphydrus* sp., *Laccophilus* sp., different Bidessini, and occasionally *Cybister* species.

All species are capable to flight. Referring to the label data specimens of *Sandracottusbakewelliibakewellii*, *S.festivus*, *S.femoralis*, *S.hunteri*, and *S.rotundus* were obtained by operating light traps.

### ﻿Zoogeographic conclusions

A total of 1,649 specimens were studied for this revision, including all the available material from relevant museums in Europe and Australia. Thanks to this large amount of old and freshly collected material, it is now possible to draw some zoogeographic conclusions. The genus *Sandracottus* is distributed throughout the Oriental and Australasian realms, and occurs in the north from east Nepal, throughout central and southern China, South Korea, the Philippines to Borneo, Sumatra, Java, and Australia in the south. In the east, the distribution of two probably sister species *S.hunteri* and *S.chevrolati* are strictly defined by the Wallace line as *S.hunteri* is replaced on Sulawesi, from the Sunda Islands east of Bali, Timor, and Tanimbar by *S.chevrolati*. In the west *S.dejeanii* can be found in some wadis of Baluchistan, south-eastern Iran. Only one species, *S.femoralis*, is widespread in New Guinea and on the Moluccas. *Sandracottusbakewellii* is endemic to Australia with one subspecies in the north and along the east coast south to Brisbane (*S.b.bakewellii*), and a separated subspecies with a limited distribution in the red center of the continent (*S.b.guttatus*). *Sandracottushunteri* is the most widespread species of the genus and is distributed from India, central and southern China, to southern Korea and the whole Southeast Asian region, excluding Borneo, west of the Wallace line. The four remaining species have very restricted distributions. One species is restricted to the Philippines (*S.insignis*), one to Borneo (*S.bizonatus*), and another to Sulawesi and some adjacent islands (*S.rotundus*). The species with the most restricted distribution is *S.jaechi*, known only from Nuwara Eliya, a mountain region (1300 m a.s.l.) in central Sri Lanka. In Malaysia, Indonesia, Thailand, and Vietnam the genus can be found at altitudes from around sea level (*S.hunteri*, *S.chevrolati*, *S.insignis*, *S.rotundus*, and *S.maculatus*) to 700 and 800 m a.s.l. (*S.femoralis*, *S.maculatus*). In India, China, and Pakistan *S.dejeanii*, *S.festivus*, and *S.hunteri* can be found from 100 to more than 2000 m. The altitudinal record is held by a single *S.hunteri* collected at 2900 m in Darjeeling District in northeastern India. The maps (Figs 23–30) show that more than half of the species occur in the central part of the Oriental region: in Myanmar, Thailand, Laos, Vietnam, Malaysia, Indonesia, and Philippines. This region can certainly be considered as a center of speciation of the genus *Sandracottus*.

### ﻿Conservation aspects

The Southeast Asian species *S.bizonatus*, *S.insignis*, and *S.rotundus* are associated with lentic or slow flowing habitats (pools, puddles, smaller lakes, and ditches) situated in the declining primary lowland rainforests on the islands Sulawesi, Borneo, and the Philippines. Based on the number of old specimens of *S.bizonatus* and *S.insignis* found in museum collections, it seems that both species were more common at the end of the 19^th^ and the beginning of the 20^th^ century than they are today. The highly endangered if not extinct *S.jaechi*, only known from three historical specimens collected at least more than one hundred years ago, is associated with the almost destroyed peatland habitats in the mountains of Sri Lanka. All four mentioned species are strongly recommended to be listed in the next IUCN red data book. Further investigations, especially on Sri Lanka, Borneo, Sulawesi, and the Philippines are needed to clarify their actual conservation status.

## Supplementary Material

XML Treatment for Sandracottus

XML Treatment for Sandracottus bakewellii bakewellii

XML Treatment for Sandracottus bakewellii guttatus

XML Treatment for Sandracottus bizonatus

XML Treatment for Sandracottus chevrolati

XML Treatment for Sandracottus dejeanii

XML Treatment for Sandracottus femoralis

XML Treatment for Sandracottus festivus

XML Treatment for Sandracottus hunteri

XML Treatment for Sandracottus insignis

XML Treatment for Sandracottus jaechi

XML Treatment for Sandracottus maculatus

XML Treatment for Sandracottus rotundus

## ﻿Additional information

### Author ORCIDs

Lars Hendrich https://orcid.org/0000-0001-8366-0749

### Data availability

All of the data that support the findings of this study are available in the main text.
